# Evaluation of Genetics in the Association Between Cardiorespiratory Fitness and Health: Insights from a Genome‐Wide Polygenic Score for Cardiorespiratory Fitness in the FinnGen and HUNT cohorts

**DOI:** 10.1111/sms.70291

**Published:** 2026-05-05

**Authors:** L. Joensuu, V. Lukander, P. Herranen, N. P. Tynkkynen, U. Kujala, R. Lopéz‐Bueno, A. N. Nordeidet, M. Klevjer, K. Øvretveit, U. Wisløff, A. Bye, U. Ekelund, M. Ollikainen, FinnGen, Aarno Palotie, Mark Daly, Bridget Riley‐Gills, Howard Jacob, Coralie Viollet, Slavé Petrovski, Alix Berton, Santha Ramakrishnan, Ellen Tsai, Zhihao Ding, Emily Holzinger, Robert Plenge, Joseph Maranville, Mark McCarthy, Rion Pendergrass, Jonathan Davitte, Simonne Longerich, Anders Mälarstig, Anna Vlahiotis, Katherine Klinger, Clement Chatelain, Jorg Blankenstein, Karol Estrada, Robert Graham, Dawn Waterworth, Chris O´Donnell, Nicole Renaud, Tomi P. Mäkelä, Jaakko Kaprio, Minna Ruddock, Petri Virolainen, Antti Hakanen, Terhi Kilpi, Markus Perola, Jukka Partanen, Taneli Raivio, Raisa Serpi, Teija Kekonen, Kati Kristiansson, Veli‐Matti Kosma, Jari Laukkanen, Tom Southerington, Outi Tuovila, Jeffrey Waring, Bridget Riley‐Gillis, Fedik Rahimov, Ioanna Tachmazidou, Alix Berton, Santha Ramakrishnan, Ellen Tsai, Zhihao Ding, Marc Jung, Hanati Tuoken, Shameek Biswas, Benjamin Sun, Rion Pendergrass, Jonathan Davitte, Neha Raghavan, Adriana Huertas‐Vazquez, Jae‐Hoon Sul, Anders Mälarstig, Xinli Hu, Åsa Hedman, Katherine Klinger, Robert Graham, Dawn Waterworth, Nicole Renaud, Ma´en Obeidat, Jonathan Chung, Jonas Zierer, Mari Niemi, Samuli Ripatti, Johanna Schleutker, Markus Perola, Mikko Arvas, Olli Carpén, Reetta Hinttala, Johannes Kettunen, Arto Mannermaa, Katriina Aalto‐Setälä, Mika Kähönen, Jari Laukkanen, Johanna Mäkelä, Lila Kallio, Tiina Wahlfors, Jukka Partanen, Eero Punkka, Raisa Serpi, Sanna Siltanen, Veli‐Matti Kosma, Tiina Jokela, Anu Jalanko, Auli Toivola, Denise Öller, Helen Cooper, Mervi Aavikko, Risto Kajanne, Rodos Rodosthenous, Sofia Kuitunen, Tarja Laitinen, Arto Lehisto, Hafiz Sikandar, Juha Karjalainen, Juha Mehtonen, Masahiro Kanai, Mitja Kurki, Mutaamba Maasha, Pietro Della Briotta Parolo, Samuel Jones, Sanni Ruotsalainen, Susanna Lemmelä, Wei Zhou, Aki Havulinna, L. Elisa Lahtela, Mari Kaunisto, Awaisa Ghazal, Elina Kilpeläinen, Jaska Uimonen, Oluwaseun Alexander Dada, Rigbe Weldatsadik, Sanni Ruotsalainen, Tianduanyi Wang, Timo P. Sipilä, Kati Donner, Anu Loukola, Päivi Ingalsuo, Arto Pietilä, Sami Koskelainen, Susanna Lemmelä, Teemu Paajanen, Tero Hiekkalinna, Priit Palta, Dawit A. Yohannes, Harri Siirtola, Javier Gracia‐Tabuenca, Marika Kaakinen, Mary Pat Reeve, Shanmukha Sampath Padmanabhuni, Shuang Luo, Vincent Llorens, Iina Laak, Jaakko Tyrmi, Janne Isojärvi, Tero Sievänen, Timo Pohjonen, Vidal Fey, Johanna Mäkelä, Pauli Wihuri, Tom Southerington, Meri Lähteenmäki, Reetta Kälviäinen, Valtteri Julkunen, Hilkka Soininen, Anne Remes, Mikko Hiltunen, Jukka Peltola, Minna Raivio, Pentti Tienari, Juha Rinne, Roosa Kallionpää, Juulia Partanen, Adam Ziemann, Nizar Smaoui, Anne Lehtonen, Susan Eaton, Shameek Biswas, Natalie Bowers, Edmond Teng, Rion Pendergrass, Fanli Xu, Laura Addis, John Eicher, Qingqin S Li, Karen He, Ekaterina Khramtsova, Neha Raghavan, Martti Färkkilä, Jukka Koskela, Sampsa Pikkarainen, Airi Jussila, Katri Kaukinen, Timo Blomster, Mikko Kiviniemi, Markku Voutilainen, Mark Daly, Jeffrey Waring, Nizar Smaoui, Fedik Rahimov, Anne Lehtonen, Tim Lu, Natalie Bowers, Rion Pendergrass, Linda McCarthy, Amy Hart, Meijian Guan, Jason Miller, Kirsi Kalpala, Melissa Miller, Xinli Hu, Kari Eklund, Antti Palomäki, Pia Isomäki, Laura Pirilä, Oili Kaipiainen‐Seppänen, Johanna Huhtakangas, Nina Mars, Jeffrey Waring, Fedik Rahimov, Apinya Lertratanakul, Nizar Smaoui, Anne Lehtonen, Coralie Viollet, Marla Hochfeld, Natalie Bowers, Rion Pendergrass, Jorge Esparza Gordillo, Dawn Waterworth, Fabiana Farias, Kirsi Kalpala, Nan Bing, Xinli Hu, Tarja Laitinen, Margit Pelkonen, Paula Kauppi, Hannu Kankaanranta, Terttu Harju, Riitta Lahesmaa, Nizar Smaoui, Coralie Viollet, Susan Eaton, Hubert Chen, Rion Pendergrass, Natalie Bowers, Joanna Betts, Kirsi Auro, Rajashree Mishra, Majd Mouded, Debby Ngo, Teemu Niiranen, Felix Vaura, Veikko Salomaa, Kaj Metsärinne, Jenni Aittokallio, Mika Kähönen, Jussi Hernesniemi, Daniel Gordin, Juha Sinisalo, Marja‐Riitta Taskinen, Tiinamaija Tuomi, Timo Hiltunen, Jari Laukkanen, Amanda Elliott, Mary Pat Reeve, Sanni Ruotsalainen, Dirk Paul, Natalie Bowers, Rion Pendergrass, Audrey Chu, Dermot Reilly, Mike Mendelson, Jaakko Parkkinen, Melissa Miller, Tuomo Meretoja, Heikki Joensuu, Olli Carpén, Johanna Mattson, Eveliina Salminen, Annika Auranen, Peeter Karihtala, Päivi Auvinen, Klaus Elenius, Johanna Schleutker, Esa Pitkänen, Nina Mars, Mark Daly, Relja Popovic, Jeffrey Waring, Bridget Riley‐Gillis, Anne Lehtonen, Margarete Fabre, Jennifer Schutzman, Natalie Bowers, Rion Pendergrass, Diptee Kulkarni, Alessandro Porello, Andrey Loboda, Stefan McDonough, Kai Kaarniranta, Joni A Turunen, Terhi Ollila, Hannu Uusitalo, Juha Karjalainen, Esa Pitkänen, Mengzhen Liu, Erich Strauss, Natalie Bowers, Hao Chen, Rion Pendergrass, Kaisa Tasanen, Laura Huilaja, Katariina Hannula‐Jouppi, Teea Salmi, Sirkku Peltonen, Leena Koulu, Nizar Smaoui, Fedik Rahimov, Anne Lehtonen, David Choy, Rion Pendergrass, Dawn Waterworth, Kirsi Kalpala, Ying Wu, Pirkko Pussinen, Aino Salminen, Tuula Salo, David Rice, Pekka Nieminen, Ulla Palotie, Maria Siponen, Liisa Suominen, Päivi Mäntylä, Ulvi Gursoy, Vuokko Anttonen, Kirsi Sipilä, Rion Pendergrass, Hannele Laivuori, Venla Kurra, Laura Kotaniemi‐Talonen, Oskari Heikinheimo, Ilkka Kalliala, Lauri Aaltonen, Varpu Jokimaa, Johannes Kettunen, Marja Vääräsmäki, Outi Uimari, Laure Morin‐Papunen, Maarit Niinimäki, Terhi Piltonen, Katja Kivinen, Elisabeth Widen, Taru Tukiainen, Mary Pat Reeve, Mark Daly, Niko Välimäki, Eija Laakkonen, Jaakko Tyrmi, Heidi Silven, Eeva Sliz, Riikka Arffman, Susanna Savukoski, Triin Laisk, Natalia Pujol, Mengzhen Liu, Bridget Riley‐Gillis, Rion Pendergrass, Janet Kumar, Iiris Hovatta, Erkki Isometsä, Hanna Ollila, Jaana Suvisaari, Antti Mäkitie, Argyro Bizaki‐Vallaskangas, Sanna Toppila‐Salmi, Tytti Willberg, Elmo Saarentaus, Antti Aarnisalo, Eveliina Salminen, Elisa Rahikkala, Johannes Kettunen, Kristiina Aittomäki, Fredrik Åberg, Joel Rämö, Mark Daly, Mary Pat Reeve, Muhammad Adnan Khan, Johanna Mäkelä, Ilkka Immonen, Kai Kaarniranta, Joni A Turunen, Anneke Den Hollander, Bridget Riley‐Gillis, Mengzhen Liu, Nizar Smaoui, Fabio Baschiera, Hans van Leeuwen, Elke Markert, Brian Yaspan, Charli Harlow, Lea Sarow‐Blat, Dermont Reilly, P. Dunnmon, Sara Gale, Fabiana Farias, Jorge Del‐aguila, Catherine O’Riordan, Samuel Lessard, Suzanne Jacobs, Satu Koskela, Anne Kerola, Elisa Lahtela, Helen Cooper, Johanna Paltta, Jukka Koskela, Mark Daly, Mary Pat Reeve, Vincent Llorens, Martti Färkkilä, Johannes Kettunen, Kaisa Tasanen‐Maatta, Laura Huilaja, Minna Ruddock, Aki Havulinna, Antti Palomäki, Laura Kuusalo, Laura Pirilä, Fedik Rahimov, Jan Freudenberg, Nizar Smaoui, Bram Prins, Coralie Viollet, Eleanor Wheeler, Kousik Kundu, Santosh Atanur, Hans van Leeuwen, Himanshu Manchanda, Karl Heilbron, Martin Rao, Nicole Schmidt, Samu Kurki, Ellen Tsai, Ketian Yu, Stephanie Loomis, Benjamin Sun, Cara Carty, Emily Holzinger, Michael Turchin, Neelakshi Jog, Frank Li, Zhihao Ding, Cameron Adams, Mark McCarthy, Michael Rothenberg, Rion Pendergrass, Diana L.Cousminer, Jagtar Nijjar, Jessica Chao, Joanna C.Betts, Jonathan M.Davitte, Linda McGarthy, Michal Magid, Shashank Jariwala, Dawn Waterworth, Amy Hart, Brice Keyes, John Kwon, Jonathan Sherlock, Matt Loza, Elisabeth Vollmann, Jozsef Karman, Julie Fiore, Rajesh Kamath, Enrico Ferrero, Jonas Zierer, Nikos Patsopoulos, Erin Macdonald‐Dunlop, Jessica Chung, Michael McLean, Hamid Mattoo, Aarno Palotie, Elisa Lahtela, Helen Cooper, Jukka Koskela, Mark Daly, Mary Pat Reeve, Raymond Walters, Rodos Rodosthenous, Jouni Lauronen, Adrian Banerji, Matthew Sampson, Michelle McNulty, Daniel Gordin, Patrik Finne, Mika Kähönen, Tapio Hellman, Teemu Niiranen, Dirk Paul, Ioanna Tachmazidou, Hans van Leeuwen, Johanna Mielke, Juho Immonen, Thomas Battram, Tobias Hogrebe, Ketian Yu, Benjamin Sun, Janie Shelton, Yao Hu, Zhihao Ding, Rion Pendergrass, Sergio Dellepiane, Audrey Chu, Chris Floyd, Dan Swerdlow, Erding Hu, Jonathan Davitte, Prerak Desai, Stephen Haddad, Dermot Reilly, P. Dunnmon, Karol Estrada, Rob Graham, Sahar Mozzafari, Nancy Finkel, Sabina Pfister, Shola Richards, Joshua Chiou, Ying Wu, Katherine Klinger, Matti Vuori, Teemu Niiranen, Bridget Riley‐Gillis, Nizar Smaoui, Alix Berton, Hans van Leeuwen, Chen Li, Emily Holzinger, Anubha Mahajan, Mark Mccarthy, Christopher Deboever, Karol Estrada, Robert Graham, Hye In Kim, Sivakumar Pitchumani, Sumedha Jassal, Åsa Hedman, Aarno Palotie, Austin Argentieri, Aoxing Liu, Eero Vuoksimaa, Elisa Lahtela, Joni Lindbohm, Mark Daly, Mary Pat Reeve, Paavo Häppölä, Zhiyu Yang, Eino Solje, Mikko Hiltunen, Valtteri Julkunen, Ville Leinonen, Hanna Kujala, Aki Havulinna, Roosa Kallionpää, Minttu Marttila, Britney Milkovich, Jan Freudenberg, Andrew Lowe, Ioanna Tachmazidou, Thomas Spargo, Kritika Singh, Peng Jiang, Stephanie Loomis, Anubha Mahajan, Rion Pendergrass, Damien Croteau‐Chonka, John Eicher, Prerak Desai, Chris Whelan, Karen He, Qingqin Li, W Galpern, Yanfei Zhang, Andrei Popescu, Delphine Fagegaltier, Mari Niemi, Nikos Patsopoulos, Katherine Klinger, Aarno Palotie, Elisa Lahtela, Jukka Koskela, Mark Daly, Sanni Ruotsalainen, Susanna Lemmelä, Tarja Laitinen, Salla Ranta, Paavo Häppölä, Paula Kauppi, Tiinamaija Tuomi, Raisa Serpi, Riitta Kaarteenaho, Hannu Kankaanranta, Coralie Viollet, Eleanor Wheeler, Oliver Burren, Christoph Ogris, Eric Simon, LI Frank, Julio Cesar Bolivar Lopez, Yao Hu, Zhihao Ding, Elena Sanchez, Emily Holzinger, Joe Maranville, Lilith Moss, Michael Turchin, Zijie Zhao, Diana Chang, Audrey Chu, Billy Fahy, Jessica Chao, Joanna Betts, Jonathan Davitte, Paola Bronson, Prerak Desai, Dermot Reilly, Mona Selej, P Dunnmon, Jorge Del‐aguila, Jozsef Karman, Travis Barr, Katherine Mccauley, Xiaobo Xia, Madhurima Saxena, Pitchumani Sivakumar, Sumedha Jassal, David Habiel, Guanling Huan, Marika Kaakinen, Mary Pat Reeve, Filip Scheperjans, Andrew Blumenfeld, Britney Milkovich, Jan Freudenberg, Tushar Kumar, Hans van Leeuwen, Juho Immonen, Samu Kurki, Coro Paisan‐Ruiz, Anna Podgornaia, Benjamin Sun, Janie Shelton, Peng Jiang, Stephanie Loomis, Tushar Bhangale, John Eicher, Abolfazl Doostparast Torshizi, Aristide Merola, Oliver Freeman, Simonne Longerich, Mari Niemi, Katherine Klinger, E. Sillanpää

**Affiliations:** ^1^ Gerontology Research Center, Faculty of Sport and Health Sciences University of Jyväskylä Jyväskylä Finland; ^2^ Institute for Molecular Medicine Finland HiLIFE Helsinki Finland; ^3^ Minerva Foundation Institute for Medical Research Helsinki Finland; ^4^ Folkhälsan Research Center Helsinki Finland; ^5^ Faculty of Sport and Health Sciences University of Jyväskylä Jyväskylä Finland; ^6^ Faculty of Health Sciences Public University of Navarra Pamplona Spain; ^7^ Department of Circulation and Medical Imaging, Faculty of Medicine and Health Sciences Norwegian University of Science and Technology Trondheim Norway; ^8^ Department of Public Health and Nursing Norwegian University of Science and Technology Trondheim Norway; ^9^ Department of Cardiology, St. Olavs Hospital Trondheim University Hospital Trondheim Norway; ^10^ Norwegian School of Sport Sciences Oslo Norway; ^11^ Department of Chronic Diseases Norwegian Institute of Public Health Oslo Norway; ^12^ Wellbeing Services County of Central Finland Jyväskylä Finland

## Abstract

We evaluated how much cardiorespiratory fitness (CRF)‐related genetics contribute to the risk of common noncommunicable diseases (NCDs) and mortality, and whether individuals with different levels of CRF and genetic predispositions differ in health characteristics. We used a validated SBayesR‐based genome‐wide polygenic score, leveraging information from 905 707 single‐nucleotide polymorphisms, to measure CRF genetics (PGS CRF). Associations with register‐based incident NCDs and mortality were analyzed using Cox proportional hazards models in the FinnGen cohort (*N* = 262 137; 53.5‐years at baseline, 52.0% women) and replicated in the HUNT3 cohort (*N* = 26 115; 59.0‐years, 52.4% women). In HUNT3, we also compared the health characteristics of individuals having age‐ and sex‐specific high or low CRF (V̇O_2peak_), and high or low PGS CRF (*N* = 1316). PGS CRF was negatively associated with any CVD (hazard ratio [HR] 0.99, 95% confidence interval 0.98–1.00), ischemic heart diseases (HR 0.98, 0.97–0.99), hypertensive diseases (HR 0.99, 0.97–1.00), stroke (HR 0.98, 0.97–1.00), lung cancer (HR 0.95, 0.92–0.97), chronic lower respiratory disease (HR 0.98, 0.97–0.99), chronic obstructive pulmonary disease (HR 0.97, 0.95–0.99), type 2 diabetes (T2D) (HR 0.96, 0.95–0.97), and all‐cause mortality (HR 0.98, 0.97–0.99), per each standard deviation increase in PGS. Replication analyses supported the association with T2D. No differences in health characteristics were observed by genetic predisposition to CRF, while those with high V̇O_2peak_ had a healthier profile in comparison to those with low V̇O_2peak_. The genome‐wide PGS explains only a fraction of the CRF phenotype, yet some small associations were observed, particularly for T2D incidence.

## 1 Introduction

Physical fitness is a set of attributes that people have or achieve and which relate to their ability to perform physical activity [1]. Cardiorespiratory fitness (CRF) is the ‘integrated ability to transport oxygen from the atmosphere to the mitochondria to perform physical work’ [2] and among the most studied aspects of physical fitness. CRF is associated with non‐communicable diseases (NCDs) throughout the lifespan [2, 3, 4]. The world's leading NCDs include heart disease, cancer, chronic respiratory disease, and diabetes [5]. A recent umbrella review with 20.9 million individual observations showed that higher CRF is systematically associated with a lower risk of incident hypertension, heart failure, stroke, atrial fibrillation, type 2 diabetes (T2D), and cardiovascular, sudden cardiac, all‐cancer, and lung cancer mortality [6]. In quantitative estimates, one metabolic equivalent of task (1‐MET) increase in CRF was associated with a lower risk of cardiometabolic diseases (disease‐specific hazard ratios [HRs] ranging from 0.82 to 0.97) and with lower risk of all‐cause mortality (HRs from 0.83 to 0.89) [6].

CRF is, however, known to be strongly influenced by genetics [7]. Meta‐analyzed heritability estimates derived from twin and family studies range from 44% to 68%, depending on the study and measure of CRF [8]. Genetic correlation studies indicate shared genetic material between CRF and several NCD risk factors [9]. Furthermore, some of the genetic variants related to CRF are expression quantitative trait loci that may influence gene expression in the heart, artery, lung, and adipose tissue [9, 10]. Hence, a favorable genotype in the context of CRF may act as the basis for better structure and function of the lungs, heart, vasculature, and cellular metabolism and influence systemic inflammation and immune function—all aspects highly involved in both CRF and common NCDs [4, 9]. However, more evidence is needed to evaluate whether genetics meaningfully explain the beneficial associations of high CRF.

It also remains unclear whether individuals with similar genotypes but differing CRF levels differ in their health. Studies have found mixed evidence showing that twin pairs discordant for CRF did not differ in baseline characteristics or in the incidence of later cardiovascular disease (CVD) [11]. However, former elite endurance athletes lived 2.4 years longer than their brothers [12].

Genome‐wide polygenic scores (PGSs) can be used to estimate an individual's genetic makeup more accurately than was previously possible [13]. A recent pioneering study using genome‐wide PGS reported small effect sizes between CRF genetics, CVDs, and mortality [14]. In the current study, we developed and applied a PGS to evaluate how much CRF‐related genetics contribute to the risk of all major NCDs and all‐cause mortality. We furthermore evaluated differences in health characteristics among individuals with different levels of measured CRF and genetic predisposition to CRF. We hypothesized that (1) PGS CRF is negatively associated with the risk of incident CVD, cancer, chronic respiratory disease, T2D, and all‐cause mortality with small to medium effect sizes and conservatively that (2) Higher CRF is associated with better health regardless of genetic predisposition.

## 2 Methods

**FIGURE 1:**
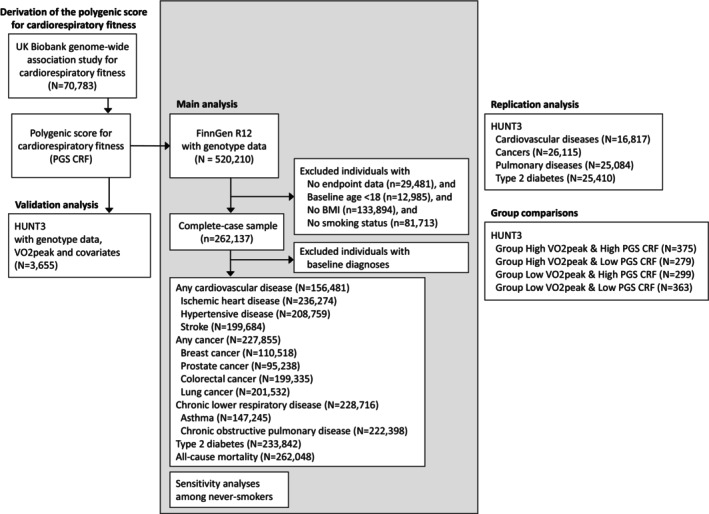
Study design and flowchart.

The study design is illustrated in Figure 1. We utilized the prospective FinnGen cohort for the main analysis and HUNT3 for validation, replication, and group comparisons (Figure 1). We also conducted additional sensitivity analyses in FinnGen and exploratory analyses in HUNT3. This study is reported in accordance with the STROBE statement [15].

### 2.1 Study Cohorts

In short, FinnGen is an ongoing Finnish public–private partnership research project linking genotype and digital health registry data using personal identification numbers [16]. The recruitment is operated mainly via healthcare contacts. FinnGen data release 12 comprises 520 210 participants with genotype data (~9.4% of the Finnish population) (https://www.finngen.fi/en). The complete case sample (*N* = 262 137) was derived as follows: 490 729 had endpoint data, 477 744 were at least 18 years old, 343 850 had BMI, and 262 137 additionally smoking data. Notably, BMI and smoking had a considerable number of missing data, 28% and 41%, respectively. We used outcome‐specific subsamples in our analyses (ranging from *n* = 95 238 to *n* = 262 048) including only participants without a diagnosis of the endpoint of interest at baseline.

HUNT (the Trøndelag Health Study) is a large, population‐based cohort study conducted in the Trøndelag region of Norway integrating health survey data, biological samples, and national health registries [17, 18]. The study includes one of the largest available datasets for CRF measured by indirect calorimetry. Since its inception in 1984, phenotypic and genetic data have been collected in several waves (HUNT1–HUNT4, https://www.ntnu.edu/hunt) [19]. A total of 50 800 individuals aged 20 years and older participated to HUNT3 in 2006–2008. Study sample selection followed similar principles as in FinnGen.

### 2.2 Outcomes

The selection of NCDs was based on the World Health Organization's criteria for the most common NCDs [5]. We also used all‐cause mortality as an outcome. FinnGen data originate from the Care Register for Health Care (available from 1968), Finnish Cancer Registry (1953), Causes of Death (1969), and Finnish Social Insurance Institution Drug Purchase registers (1995). Any CVD, with subcategories of ischaemic heart diseases, hypertension, and stroke; any cancer (subcategories: breast, prostate, colorectal, and lung); chronic lower respiratory disease (subcategories: asthma and chronic obstructive pulmonary disease [COPD]), and T2D were used as outcomes. The selection of the subcategories was based on the most prevalent endpoints in the FinnGen R12 cohort. Relevant ICD‐10 codes and FinnGen labels for data browsing are available in Table S1. We utilized the same ICD codes in the HUNT replication analyses. The HUNT data are from the Nord‐Trøndelag Health Trust discharge register (available from 1987).

### 2.3 Exposure

We chose the largest genome‐wide association study (GWAS) for CRF available at the time of the analysis as the base data for PGS CRF after careful evaluation [9]. Specifically, the PGS was based on submaximal fitness test, not measured V̇O_2max_. The publicly available summary statistics indicate each single‐nucleotide polymorphism's (SNP's) effect size on CRF. The effect sizes are based on information from 70 783 genotyped UK Biobank participants who had undergone a submaximal cycle ergometer test. In the GWAS, extrapolated estimated maximal workload relative to body weight in kilograms (watts/kg) represents CRF. Workload and heart rate data were collected during a 6‐min exercise period where participants screened to have ‘minimal’ or ‘small’ risk categories had their workload increased, and a ‘medium’ risk group kept their workload constant during the test. Maximal workload was thereafter extrapolated using age‐estimated maximum heart rate. The risk category was considered a covariate in GWAS analyses [9]. The full test protocol is published elsewhere [20]. The GWAS identified 12 statistically significant SNPs and multiple functionally relevant genes [9]. The SNP heritability was 10.5% based on Complex‐Traits Genetics Virtual Lab [21], indicating its suitability as base data for constructing a PGS [13].

We used the SBayesR method to construct the PGS CRF for each participant [22]. First, we retrieved the reported weights for 8 918 920 SNPs. Then, we restricted this genetic information to 1 074 896 HapMap3 SNPs to ensure computational efficiency while maintaining the genome‐wide representation, focusing on common variants (minor allele frequency, MAF > 5%) and good imputation coverage [23]. To correct for weight inflation caused by linkage disequilibrium (LD), we subsequently applied an LD‐reference panel consisting of 50 000 randomly selected UK Biobank participants. These procedures resulted in 916 113 SNPs and their relevant weights, which were used to calculate a PGS CRF for each participant in FinnGen and HUNT3. The full pipeline is described elsewhere [24]. The final number of processed variants was 905 707 in FinnGen and 915 971 in HUNT3. We used a standardized PGS score in the analyses (mean of 0, standard deviation [SD] of 1).

The constructed PGS CRF showed small but statistically significant associations against the gold standard measure of CRF, measured by indirect calorimetry during a maximal graded treadmill test (utilizing an evaluation of reaching a plateau in oxygen consumption despite increased workload, and respiratory exchange ratio of > 1.05) (Cortex MetaMax II, Cortex Biophysik GmbH, Leipzig, Germany) [25]. The PGS CRF explained 0.20% of variation in V̇O_2peak_ mL/kg/min and 0.21% in V̇O_2peak_ mL/kg^0.75^/min. The observed associations were comparable in magnitude to those of previously validated exercise‐related polygenic scores associated with incident NCDs [26, 27, 28, 29]. We also tested two alternative PGSs in which (1) the SNP weights were based on a GWAS for V̇O_2max_ scaled to fat‐free mass [30], and scoring as previously described (SBayesR method) (PGS CRF FFM), and (2) based on favorable SNPs [30] and their summarized effect sizes (GRS CRF). These alternative scores did not, however, improve the variation explained in measured CRF and were therefore excluded from further analyses (see Table S2 for validation cohort descriptives, Table S3 for detailed results, Figure S1 for density plots, and Figure S2 for a correlation heatmap between genetic scores and selected markers of CRF).

#### 2.3.1 Genotyping, Quality Control, and Imputation

The FinnGen samples were genotyped with Illumina and Affymetrix chip arrays (Illumina Inc., San Diego, and Thermo Fisher Scientific, Santa Clara, CA, USA). Detailed genotyping, quality control, and imputation information are available elsewhere: https://finngen.gitbook.io/documentation/. In HUNT3, standard and customized HumanCoreExome arrays from Illumina were used. Full details of genotyping, imputation, and quality control can be found in other sources [19].

### 2.4 Covariates

Covariates were selected based on prior literature to account for potential biases arising from genotyping procedures and individual characteristics. The first 10 principal components of ancestry adjust for genetic stratification [31]. Genotyping batch (batch no.) was included to correct for potential systematic biases introduced by the genotyping process. Body mass index (BMI, kg/m^2^), smoking status (never, former, current), and sex (male, female) were obtained from the Finnish Biobanks and HUNT and included both self‐reported and measured data [16, 19].

### 2.5 Statistical Analysis

#### 2.5.1 Survival Analyses

We used Cox proportional hazard models and R package survival [32] to estimate the HRs and 95% confidence intervals (CI) between PGS CRF and incident NCDs and mortality in the FinnGen cohort. Visual inspection of Schoenfeld residuals and log‐minus‐log plots indicated that the fully adjusted models satisfy proportional hazard assumptions [33]. Previous studies have found sex interactions between fitness‐related PGSs and health outcomes [34]. PGS × SEX interaction was tested, but no replicable sex differences were observed (all *p*_interaction_ > 0.370); thus, analyses were conducted with men and women combined, except for breast and prostate cancer which were analyzed sex‐specifically. Analyses were restricted to participants with complete‐case data. We used age as the timescale. Start of follow‐up was set at each participant's baseline age (age at blood sampling) to ensure that the study mimicked the prospective designs of previous CRF literature. To capture incident events, we excluded individuals with a diagnosed endpoint of interest prior to baseline. Follow‐up ended with whichever came first among the first record of the endpoint of interest, death, or the end of follow‐up on 31 December 2021. Underaged individuals (< 18‐years) were excluded to maintain consistency between the FinnGen and HUNT cohorts. Model 1 included sex (except for breast and prostate cancer), the first 10 principal components of ancestry, and genotyping batch. Model 2 was Model 1 + BMI, Model 3 was Model 1 + smoking, and Model 4 was Model 1 + BMI and smoking.

We report results from Model 1 as our main results, as we observed that higher PGS CRF was associated with lower likelihood of smoking and lower BMI (odds ratio for being a current smoker referenced to never smokers: 0.95, *p* < 0.001; and *β* −0.22, *p* < 0.001 for BMI kg/m^2^, per one standard deviation increase in PGS CRF), thus smoking and BMI may not act as simple covariates. We additionally report the associations of PGS CRF and health outcomes adjusted for BMI and smoking in different adjustment sets. We also performed additional analyses using a categorical PGS CRF divided into quintiles. Utilizing the weights of the European standard population [35], age‐standardized incidence was calculated when relevant. The statistical significance level was set to < 0.05. Due multiple number of tests, we applied false discovery rate (FDR) correction for *p*‐values.

#### 2.5.2 Sensitivity Analyses

As a sensitivity analysis, we restricted the Cox proportional hazards model to never‐smokers in the FinnGen study. The model specifications were otherwise identical to those used in the main survival analyses.

#### 2.5.3 Replication Analyses

The replication analyses in HUNT3 were conducted similarly to the main analyses in FinnGen, except that in the absence of mortality data, the follow‐up was defined as the first record of the endpoint of interest or last verified contact with healthcare until the end of year 2024. To ensure sufficient sample size, outcomes were restricted to main disease categories: CVDs, cancer, pulmonary diseases, and T2D.

#### 2.5.4 Group Comparisons

A priori power calculations were used to determine sufficient sample size and group cut‐offs (expected small effect size, power at 0.80, *N* > 400). Groups were formed based on individuals in the ≥ 70th and ≤ 30th percentile in age‐ and sex‐specific V̇O_2peak_ and PGS CRF. Age was handled in 5‐year bins separately for both sexes. Individuals with high V̇O_2peak_ and high PGS CRF (both ≥ 70th percentile) were group 1 (Table S4). Individuals with high V̇O_2peak_ but low PGS CRF (≤ 30th percentile) were group 2. Individuals with low V̇O_2peak_ and high PGS CRF were group 3, and individuals with low V̇O_2peak_ and low PGS CRF (both ≤ 30th percentile) were group 4. The differences between the groups in health‐related characteristics were evaluated for continuous variables with ANOVA. Assumptions of normality were inspected visually and/or with Shapiro–Wilk's test, homogeneity with Levene's Test, and statistical differences with Tukey's HSD test, or if Levene's test failed, with Welch‐ANOVA and Games‐Howall post hoc test. For categorical variables, the differences were tested with the chi‐squared test. Variables which considerably deviated from normal distribution (such as MET h/week and triglycerides) were square‐root and log‐transformed respectively. As the observed statistical associations did not differ between original and transformed values, only original values are shown. We also used Cox proportional hazards models only as exploratory analyses to investigate disease incidence between the groups (due limited number of cases).

## 3 Results

**FIGURE 2:**
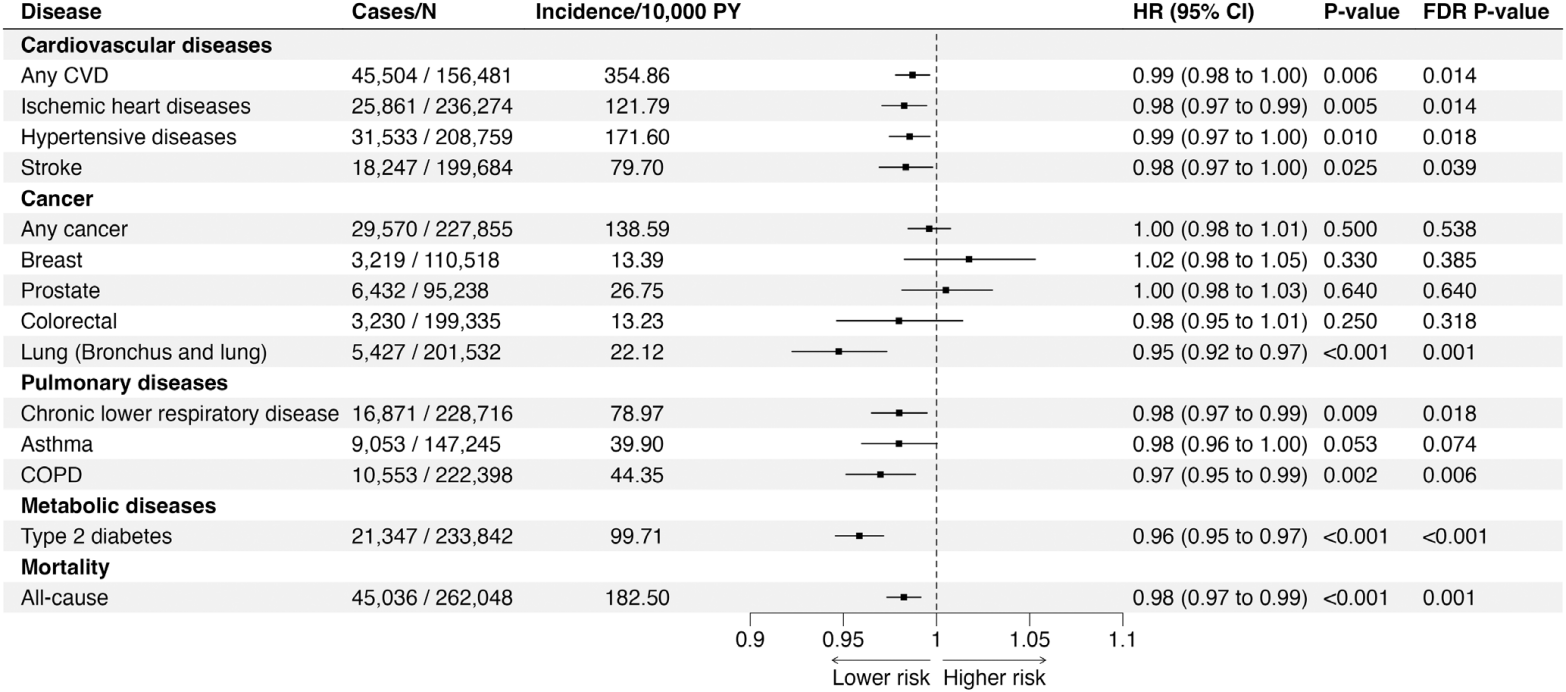
Associations of polygenic score for cardiorespiratory fitness with common non‐communicable diseases and all‐cause mortality in the minimally adjusted model. The model was adjusted for sex (except for breast and prostate cancer), the first 10 principal components of ancestry, and genotyping batch. For each endpoint, the analytical sample includes only participants at risk for incident disease at baseline (i.e., prevalent cases excluded), resulting in endpoint‐specific numbers for included controls and cases.

Descriptive data for the complete‐case sample (*N* = 262 137) in FinnGen is shown in Table 1. The incidence and follow‐up times were disease‐specific. For example, 17.4% participants were diagnosed with any CVD (45 504 cases) with an age‐standardized incidence of 354.86 per 10 000 person‐years (Figure 2). The average follow‐up for any CVD was 9.23 years and a total of 419 935 person‐years.

**TABLE 1:** Baseline characteristics of the complete‐case sample in FinnGen.

Characteristic	All (*n* = 262 137)	Women (*n* = 136 331)	Men (*n* = 125 806)
Age (years)	53.5 (16.0)	51.4 (16.6)	55.8 (15.0)
BMI (kg/m^2^)	27.4 (5.3)	27.4 (5.8)	27.5 (4.6)
Height (cm)	170.4 (9.1)	164.4 (6.4)	176.9 (6.9)
Weight (kg)	79.8 (17.3)	74.01 (16.4)	86.0 (16.1)
Smoking status, *n* (%)			
Never	130 211 (49.7)	82 797 (60.7)	47 414 (37.7)
Former	61 788 (23.6)	28 499 (20.9)	33 289 (26.5)
Current	70 138 (26.8)	25 035 (18.4)	45 103 (35.9)

*Note:* Values are means (standard deviations) for continuous variables and *n* (%) for categorical/binary.

Abbreviation: BMI, body mass index.

### 3.1 Associations Between PGS CRF, Morbidity, and Mortality

Each SD increase in PGS CRF was associated with reduced risk of all evaluated CVDs: any CVD (HR 0.99, 0.98–1.00, FDR *p*‐value = 0.014; Figure 2), ischemic heart disease (HR 0.98, 0.97–0.99, *p* = 0.014), hypertensive disease (HR 0.99, 0.97–1.00, *p* = 0.018), and stroke (HR 0.98, 0.97–1.00, *p* = 0.039). From cancers, the PGS CRF was associated only with reduced risk of lung cancer (HR 0.95, 0.92–0.97, *p* = 0.001). From pulmonary diseases, PGS CRF was associated with reduced risk of chronic lower respiratory disease (HR 0.98, 0.97–0.99, *p* = 0.018), and COPD (HR 0.97, 0.95–0.99, *p* = 0.006), but not with asthma (HR 0.98, 0.96–1.00, *p* = 0.074). PGS CRF was additionally associated with T2D (HR 0.96, 0.95–0.97, *p* < 0.001) and all‐cause mortality (HR 0.98, 0.97–0.99, *p* = 0.001).

The associations for different adjustment sets are available in the Supplement (Figures S3−S5). Following adjustment with BMI, the associations with CVDs attenuated, while other observed associations remained statistically significant (Figure S3). Adjusting with smoking altered the associations only marginally (Figure S4). In the model adjusted with both BMI and smoking, we observed statistically significant associations only for lung cancer (HR 0.95, 0.93–0.98, *p* = 0.003), T2D (HR 0.98, 0.96–0.99, *p* = 0.003) and all‐cause mortality (HR 0.98, 0.98–0.99, *p* = 0.005; Figure S5). We inspected the associations adjusted for BMI and smoking also with cumulative incidence plots among high PGS CRF (> 90th percentile) and low PGS CRF (< 10th percentile) individuals. The data indicated high PGS CRF to be associated with lower all‐cause mortality throughout the lifespan, while the association for T2D and lung cancer were more prevalent after midlife or in old age (Figure S6).

There were no statistically significant association in sensitivity analyses among never‐smokers, indicating that smoking may have a substantial role in the observed associations (Figure S7). Further analyses using PGS quintiles indicated that breast cancer, colorectal cancer, and asthma in particular showed considerable variation in their associations, potentially reflecting either non‐linear associations or limited number of cases (Figure S8). In replication analyses (see Table S5 for sample characteristics), the association for T2D replicated in HUNT3 although attenuated after adjustment for BMI (Model 1: HR 0.95, 0.92–0.99; Model 2: HR 0.99, 0.95–1.03; Model 3: HR 0.96, 0.92–0.99; Model 4: HR 0.99, 0.95–1.03; Table S6). The associations between PGS CRF and any CVD, any cancer, and pulmonary diseases were non‐significant in HUNT3 (HR 1.01, 0.99–1.03; HR 1.02, 0.99–1.07; and HR 0.97, 0.94–1.00, respectively, in Model 1, Table S6).

### 3.2 Health Differences Between V̇O_2peak_ and PGS CRF Groups

The groups with different levels of measured CRF and genetic predisposition for CRF had a systematic pattern in their health characteristics (Table 2). We observed that groups with higher V̇O_2peak_ differed by their health profile from those with lower V̇O_2peak_ irrespective of the PGS CRF; those with high V̇O_2peak_ had lower resting heart rate and BMI, were less likely current smokers, were physically more active, had better self‐rated health, had lower systolic blood pressure, had higher HDL cholesterol and lower LDL cholesterol, lower triglycerides and lower FINDRISC diabetes score than those with low V̇O_2peak_. Only exceptions were observed with diastolic blood pressure and total cholesterol, where not all low V̇O_2peak_ groups statistically differed from high V̇O_2peak_ groups (Table 2).

**TABLE 2:** Characteristics of individuals with high or low cardiorespiratory fitness and high or low PGS CRF.

Characteristic	*N*	(1) High V̇O_2peak_ High PGS CRF	*N*	(2) High V̇O_2peak_ Low PGS CRF	*N*	(3) Low V̇O_2peak_ High PGS CRF	*N*	(4) Low V̇O_2peak_ Low PGS CRF	*p*_difference_	Differences between groups
PGS CRF (*Z*‐score)	375	1.16 (0.5)	279	−1.16 (0.5)	299	1.13 (0.5)	363	−1.17 (0.6)	**< 0.001**	1 vs. 2 & 4; 3 vs. 2 & 4
V̇O_2peak_ (mL/kg/min)	375	48.5 (8.6)	279	48.9 (8.6)	299	33.0 (6.7)	363	32.3 (6.5)	**< 0.001**	1 vs. 3 & 4; 2 vs. 3 & 4
V̇O_2peak_ (mL/kg^0.75^/min)	375	141.0 (27.1)	279	142.0 (27.3)	299	99.5 (21.7)	363	97.3 (20.6)	**< 0.001**	1 vs. 3 & 4; 2 vs. 3 & 4
Resting heart rate	374	65.0 (11.2)	279	63.5 (9.9)	299	71.1 (10.7)	363	70.6 (10.2)	**< 0.001**	1 vs. 3 & 4; 2 vs. 3 & 4
Age (years)	375	48.7 (12.4)	279	47.7 (13.3)	299	49.2 (13.9)	363	49.9 (13.4)	0.193	
Women, *n* (%)	375	208 (55.5%)	279	145 (52.0%)	299	157 (52.5%)	363	195 (53.7%)	0.809	
SES, *n* (%)	375		279		299		363		**< 0.001**	1 vs. 3 & 4; 2 vs. 3 & 4
High	191	50.9%	153	54.8%	121	40.5%	132	36.4%		
Medium	168	44.8%	115	41.2%	162	54.2%	209	57.6%		
Low	16	4.3%	11	3.9%	16	5.4%	22	6.1%		
BMI (kg/m^2^)	375	24.2 (2.7)	279	24.0 (2.45)	299	27.7 (3.8)	363	27.9 (3.3)	**< 0.001**	1 vs. 3 & 4; 2 vs. 3 & 4
Smoking status, *n* (%)	375		279		299		363		**< 0.001**	1 vs. 3 & 4; 2 vs. 3 & 4
Never	222	59.2%	163	58.3%	126	42.1%	154	42.4%		
Former	113	30.1%	75	26.9%	110	36.8%	118	32.5%		
Current	40	10.7%	41	14.7%	63	21.1%	91	25.1%		
MET (h/week)	375	15.9 (8.6)	279	16.5 (9.3)	299	12.0 (8.8)	363	12.9 (10.0)	**< 0.001**	1 vs. 3 & 4; 2 vs. 3 & 4
Self‐reported health, *n* (%)	375		279		299		363		**< 0.001**	1 vs. 3 & 4; 2 vs. 3 & 4
Poor	0	0.0%	1	0.4%	2	0.7%	1	0.3%		
Not so good	16	4.3%	15	5.4%	43	14.4%	51	14.1%		
Good	208	55.5%	139	49.8%	198	66.2%	248	68.3%		
Very good	151	40.3%	124	44.4%	56	18.7%	63	17.4%		
Systolic blood pressure (mm/Hg)	364	126 (14.9)	267	126 (14.5)	294	130 (16.2)	355	130 (16.0)	**< 0.001**	1 vs. 3 & 4; 2 vs. 3 & 4
Diastolic blood pressure (mm/Hg)	364	72.0 (10.7)	267	72.0 (10.2)	294	74.4 (11.6)	355	74.2 (10.6)	**< 0.001**	1 vs. 3 & 4; 2 vs. 3
Total cholesterol (mmol/L)	364	5.4 (1.0)	267	5.3 (1.0)	294	5.6 (1.1)	355	5.7 (1.1)	**< 0.001**	1 vs. 4; 2 vs. 3 & 4
HDL cholesterol (mmol/L)	364	1.5 (0.4)	267	1.5 (0.4)	294	1.4 (0.3)	355	1.4 (0.3)	**< 0.001**	1 vs. 3 & 4; 2 vs. 3 & 4
LDL cholesterol (mmol/L)	364	3.6 (0.9)	267	3.5 (0.9)	294	3.9 (1.0)	355	3.8 (1.0)	**< 0.001**	1 vs. 3 & 4; 2 vs. 3 & 4
Triglycerides (mmol/L)	364	1.2 (0.6)	267	1.2 (0.6)	294	1.6 (0.9)	355	1.7 (1.0)	**< 0.001**	1 vs. 3 & 4; 2 vs. 3 & 4
Diabetes risk score (FINDRISC)	375	5.7 (3.6)	279	5.2 (3.5)	299	8.9 (4.2)	363	9.0 (4.1)	**< 0.001**	1 vs. 3 & 4; 2 vs. 3 & 4

*Note:* Values are means (standard deviations) for continuous variables and *n* (%) for categorical/binary. Analyses were conducted with ANOVA and chi‐squared test. FINDRISC diabetes score, The Finnish Diabetes Risk Score is an eight‐item scale used to predict the probability of developing type 2 diabetes within 10 years and is based on age, BMI, waist circumference, blood pressure medication, hyperglycaemia, family members with diabetes, diet and physical activity. Lower than 7 points; Low risk: estimated 1 in 100 will develop disease, 7–11 points; Slightly elevated risk: estimated 1 in 25 will develop disease. Bold values indicate statistical significance at *p* < 0.001.

Abbreviations: BMI, body mass index; HDL, high‐density lipoprotein; LDL low‐density lipoprotein; MET, metabolic equivalent of task; PGS CRF, polygenic score for cardiorespiratory fitness; SES, socioeconomic status; V̇O_2peak_, peak oxygen consumption.

In further exploratory analyses in a subsample with health register data (*N* = 452–541), both groups with low V̇O_2peak_ showed systematically higher risk estimates for T2D compared to those with high V̇O_2peak_ and high PGS CRF (HR 5.59, 1.23–25.25 for low V̇O_2peak_ and high PGS CRF group, and HR 6.20, 1.41–27.16 for low V̇O_2peak_ and low PGS CRF group) (Table S7). However, the analyses were limited by statistical power and should be interpreted with caution.

## 4 Discussion

We observed that the genome‐wide PGS CRF had a limited association with measured CRF, and the associations between PGS CRF, common NCDs and all‐cause mortality were overall small. Similarly, in group comparisons, the associations were mostly explained by the CRF phenotype instead of the CRF genotype. However, some favorable associations were observed between PGS CRF and NCDs, particularly with incident T2D.

This study was one of the first to use genome‐wide methods to estimate genetic liability to CRF. We found that each standard deviation increase in the polygenic score for CRF was associated with 0.4 mL/kg/min higher V̇O_2peak_. In comparison, a 1‐MET increase in CRF, which is considered as the minimal clinically important difference [2], is linked to an estimated 8% reduction in T2D risk [6]. When conventional transformations of 1‐MET are used, this would indicate that approximately 1% of the previously observed 8% risk would be explained by more favorable genetic predisposition [6]. We also observed that each standard deviation increase in PGS CRF was associated with an approximately 4% lower risk of T2D incidence. The association was most systematic across replication analyses, while associations for cardiovascular disease, pulmonary disease, and mortality disappeared in different adjustment sets. This association may indicate complex pathways from PGS CRF to health outcomes, which we were unable to test in this study due to the limited effect sizes.

Overall, studies utilizing genome‐wide genetic instruments for CRF are limited. Existing data support the direction and magnitude of our findings regarding T2D [30], longevity [36], CVD [14], and partially with cancer [37]. Differences in methods (e.g., varying genetic instruments for CRF) and analytical approaches challenge the comparability of studies.

CRF is a complex, polygenic trait. In polygenic traits, single SNPs explain individually only a small proportion of a phenotype [38]. Although computational genetics are constantly evolving, many scoring methods remain dependent on GWASs. Relevant GWASs are preferably based on large sample sizes to capture adequate genetic variation while including a well‐phenotyped trait of interest in a population similar to the one with which the scores are to be used. The current GWASs for CRF may align well with the broader construct of physical fitness (i.e., ability to perform physical activity), but are insufficiently harmonized with respect to the definition of CRF (i.e., the body's ability to deliver and utilize oxygen for work) [9, 10, 30]. Current GWASs for CRF range from performance phenotypes (watts/kg) [9] to resting heart rate‐derived metrics [30] and estimated [30] or directly measured oxygen consumption [10], utilizing various scaling approaches (per body weight, per fat‐free mass) [9, 10, 30]. In the future, a novel GWAS is recommended to be developed following a careful psychometric evaluation; what constitutes CRF, what does not, and which metrics demonstrate strong construct and criterion validity. More genetically informed studies using harmonized CRF testing are needed. However, since their initial releases in early 2020s, GWASs have brought new insights into the genetic basis of CRF [7]. Based on current data, CRF genetics appear to be a mixture of SNPs with both positive and negative associations [9, 10, 30]. Thus, CRF genetics may reflect not only favorable body structure and function but also absence of disease. For example, genes replicated in two UK Biobank‐based studies, *CCDC141* and *KIAA1755*, have shown positive and negative associations with CRF, respectively [9, 30]. Of note, *CCDC141* has a role in the regulation of heart rate and blood pressure, while *KIAA1755* has been associated with an increased risk of atrial fibrillation, cardioembolic stroke, and metabolic syndrome [39].

### 4.1 Strengths and Limitations

The study has several strengths, such as the use and evaluation of a novel genome‐wide genetic score, the availability of gold standard measurement for CRF, and a robust replication design with two independent populations. The study is, however, not without limitations. The current PGS CRF explains a small proportion of the variation in CRF, and the results need to be replicated when methods and available data have improved. We also measured only common variants (MAF > 5%) [23]. This approach was carefully chosen to evaluate the public health relevance of CRF genetics, that is, associations expected to be commonly observed in the population. However, the findings do not diminish the significance of potential rare genetic variants which may have larger effect sizes [40]. While such variants may be highly relevant for individuals and families, their relevance in a public health context needs further study. Also, the PGS was constructed based on submaximal fitness test results, and although other PGSs did not show better validity, they might have had different health associations. FinnGen has limited data on covariates. In particular, smoking information is limited to categorical variables and detailed measures such as smoking intensity are not available. FinnGen is based mainly on individuals having an established contact with healthcare services, which biases the cohort toward a less healthy population [16], and this may limit the generalizability of the survival analyses. In the group comparisons, conditioning on high V̇O_2peak_ may have introduced collider bias, which could obscure associations between genetic liability to CRF and health outcomes. We also note that the association between PGS CRF and outcomes was linear for most endpoints, although not for all, which may limit interpretability. Analyses were restricted to individuals with European ancestry, limiting generalizability. Finally, while competing risk analysis would have been methodologically appropriate, the limited effect sizes suggest that its applicability in this context would have been low.

## 5 Conclusions

The genome‐wide measures of genetic liability to cardiorespiratory fitness require further development, yet currently show some small associations with morbidity and mortality, and particularly for T2D incidence.

## 6 Perspective

We observed small effect sizes for PGS CRF while previous literature indicates high heritability in CRF. These higher estimates (ranging from 44% to 68%) [8] have been based on family [41] or twin designs [42]. The distinction between these study designs and our approach is important. Our study quantified individual‐level genetic liability using a DNA‐based genome‐wide polygenic score [22], whereas family and twin studies infer heritability [43]. The explanatory rate of PGS CRF, however, remains well behind that of the best polygenic scores, which hinders the implementation of robust epidemiological study designs. Improvements in PGS CRF are restricted by limited genetic data of well‐phenotyped CRF. An example of unachieved potential is the progress made with obesity‐related polygenic scores. In a recent study, a new polygenic score more than doubled the predictive power, explaining 17.6% of the variation in body mass index among UK Biobank participants [44]. The study used genetic data from over 5 million people to inform the genetic liabilities. Genome‐wide studies with increased sample sizes and advances in sequencing technology have been acknowledged as the main drivers of new discoveries. Collaborative efforts to collect large‐scale data beyond UKB and HUNT, such done in obesity sciences, are required to move the field forward.

## Author Contributions

L.J. and E.S. conceptualized the research question, and L.J. wrote a statistical analysis plan to which all authors contributed. L.J. preprocessed the publicly available genetic data. V.L. calculated the polygenic scores for FinnGen with the help of P.H., and L.J. calculated the scores for HUNT with the help of N.P.T. V.L. performed statistical modeling in FinnGen under the supervision of L.J., P.H., M.O., and E.S. L.J. performed the analyses in the HUNT in consultation with N.P.T., A.N.N., M.K., K.Ø., and A.B. E.S. secured access to FinnGen data. FinnGen authors and their roles in data management and collection are presented in the Supplement. Additionally U.K. contributed to FinnGen data collection. A.N.N., M.K., K.Ø., A.B., and U.W. contributed to HUNT data collection and management. L.J., V.L., and P.H. drafted the first version of the manuscript, and all authors contributed significantly to the writing, critical interpretation of the findings, and revising the manuscript. E.S. acquired funding for the study. L.J. is the guarantor and attests that all listed authors meet authorship criteria and that no others meeting the criteria have been omitted.

## Funding

The funders did not affect this study in any way. This work was funded by Research Council of Finland (grants 341750, 346509, and 361981), Juho Vainio Foundation; Päivikki and Sakari Sohlberg Foundation, all to ES. V.L. is funded by a University of Helsinki doctoral researcher position in the iCANDOC Doctoral Education Pilot in Precision Cancer Medicine. The FinnGen project is funded by Business Finland and 13 international pharmaceutical industry partners: AbbVie, AstraZeneca, Biogen, Boehringer Ingelheim, Celgene/Bristol‐Myers Scibb, Genentech (a member of the Roche Group), GSK, Janssen, Maze Therapeutics, MSD/Merck, Novartis, Pfizer, and Sanofi. The HUNT study is a collaboration between HUNT Research Center (Faculty of Medicine and Health Sciences, Norwegian University of Science and Technology, NTNU), Trøndelag County Council, Central Norway Regional Health Authority, and the Norwegian Institute of Public Health. The genotyping in HUNT was financed by the National Institutes of Health; University of Michigan; the Research Council of Norway; the Liaison Committee for Education, Research and Innovation in Central Norway; and the Joint Research Committee between St Olavs Hospital and the Faculty of Medicine and Health Sciences, NTNU. The genetic investigations of the HUNT study are a collaboration between researchers from the K.G. Jebsen Center for Genetic Epidemiology, NTNU, and University of Michigan Medical School, and the University of Michigan School of Public Health. The K.G. Jebsen Center for Genetic Epidemiology is financed by Stiftelsen Kristian Gerhard Jebsen; Faculty of Medicine and Health Sciences, NTNU, Norway.

## Disclosure

Transparency statement: L.J. affirms that the manuscript is an honest, accurate, and transparent account of the study being reported; that no important aspects of the study have been omitted; and that any discrepancies from the study as originally planned (and, if relevant, registered) have been explained.

## Ethics Statement

Written informed consent was obtained from all participants, and the studies were approved by ethics committees. Participants in the FinnGen cohort provided informed consent for biobank research on the basis of the Finnish Biobank Act or cohort study‐specific consent. The FinnGen study protocol (number HUS/990/2017) was approved by the Coordinating Ethics Committee of the Hospital District of Helsinki and Uusimaa. The FinnGen study is approved by the Finnish Institute of Health and Welfare (approval number THL/2031/6.02.00/2017), the Digital and Population Data Service Agency, the Social Insurance Institution, and Statistics Finland. The HUNT study was approved by the Regional Committee for Medical and Health Research Ethics (REC; 2019/29771), the Trøndelag Health Study, the Norwegian Data Inspectorate, and the National Directorate of Health. LJ is the guarantor and assumes responsibility for the accuracy and completeness of the protocol, data, and analyses, the fidelity of their reporting, and compliance with the Declaration of Helsinki, national laws, and the guidelines of the Finnish Advisory Board on Research Integrity.

## Conflicts of Interest

The authors declare no conflicts of interest.

## Supporting information


**Figure S1:** Density plots for three different genetic scores in the HUNT3; A) PGS CRF, B) PGS CRF FFM, and C) GRS CRF.
**Figure S2:** Correlation heatmap for PGS CRF, PGS CRF FFM and GRS CRF against selected markers of cardiorespiratory fitness. Values are Pearson correlation coefficients.
**Figure S3:** Associations of polygenic score for cardiorespiratory fitness with common non‐communicable diseases and all‐cause mortality following adjustment for BMI in FinnGen. The model was adjusted for sex (except for breast and prostate cancer), first 10 principal components of ancestry, genotyping batch and BMI at baseline.
**Figure S4:** Associations of polygenic score for cardiorespiratory fitness with common non‐communicable diseases and all‐cause mortality following adjustment for smoking in FinnGen. The model was adjusted for sex (except for breast and prostate cancer), first 10 principal components of ancestry, genotyping batch and smoking at baseline.
**Figure S5:** Associations of polygenic score for cardiorespiratory fitness with common non‐communicable diseases and all‐cause mortality following adjustment for BMI and smoking in FinnGen. The model was adjusted for sex (except for breast and prostate cancer), first 10 principal components of ancestry, genotyping batch, BMI and smoking at baseline.
**Figure S6:** Cumulative incidence of lung cancer, type 2 diabetes, and all‐cause mortality in individuals with high (>90^th^ percentile) and low (<10^th^ percentile) polygenic score for cardiorespiratory fitness. Cumulative incidences are from Cox proportional hazards model and adjusted for sex, the first 10 principal components of ancestry, genotyping batch, BMI, and smoking status.
**Figure S7:** Associations of polygenic score for cardiorespiratory fitness with common non‐communicable diseases and all‐cause mortality among never‐smokers in FinnGen. The model was adjusted for sex (except for breast and prostate cancer), first 10 principal components of ancestry, genotyping batch and BMI at baseline.
**Figure S8:** Hazard ratios by PGS CRF quintiles in FinnGen. The model was adjusted for sex (except for breast and prostate cancer), first 10 principal components of ancestry, and genotyping batch. The population was divided into five equal groups (20% each), ranging from Q1 (lowest genetic score) to Q5 (highest genetic score). Q3 is the middle quintile (40th to 60th percentile), and set as the reference category (HR = 1.00).
**Table S1:** Endpoint data in FinnGen R12.
**Table S2:** Descriptives of individuals in HUNT3 with measured cardiorespiratory fitness.
**Table S3:** Associations between different genetic scores and selected measures of CRF.
**Table S4:** A contingency table of the number of individuals in age‐ and sex‐specific VO2peak and PGS CRF deciles.
**Table S5:** Baseline characteristics of participants in the HUNT replication study.
**Table S6:** Associations between polygenic score for cardiorespiratory fitness and morbidity in the HUNT cohort.
**Table S7:** Exploratory analyses between cardiorespiratory fitness and genetic liability groups with morbidity in the HUNT cohort.

## Data Availability

Access to individual‐level genotypes and register data from FinnGen participants can be applied for via the Fingenious portal (https://site.fingenious.fi/en/) hosted by the Finnish Biobank Cooperative FinBB (https://finbb.fi/en/). Researchers affiliated with a Norwegian research institution can apply for HUNT data access from the HUNT Research Centre (www.ntnu.edu/hunt) if they have obtained project approval from the Regional Committee for Medical and Health Research Ethics (REC). Researchers not affiliated with a Norwegian research institution should collaborate with and apply through a Norwegian principal investigator. Information on the application and conditions for data access is available online (www.ntnu.edu/hunt/data).
