# Supplementary material for: Evaluation of Genetics in the Association Between Cardiorespiratory Fitness and Health: Insights from a Genome‐Wide Polygenic Score for Cardiorespiratory Fitness in the FinnGen and HUNT cohorts
Source: Scand J Med Sci Sports. 2026 May 5;36:e70291. doi: 10.1111/sms.70291 (PMC13145328; doi:10.1111/sms.70291)
Supplement: Supplementary file 1 — Figure S1: Density plots for three different genetic scores in the HUNT3; A) PGS CRF, B) PGS CRF FFM, and C) GRS CRF. Figure S2: Correlation heatmap for PGS CRF, PGS CRF FFM and GRS CRF against selected markers of cardiorespiratory fitness. Values are Pearson correlation coefficients. Figure S3: Associations of polygenic score for cardiorespiratory fitness with common non‐communicable diseases and all‐cause mortality following adjustment for BMI in FinnGen. The model was adjusted for sex (except for breast and prostate cancer), first 10 principal components of ancestry, genotyping batch and BMI at baseline. Figure S4: Associations of polygenic score for cardiorespiratory fitness with common non‐communicable diseases and all‐cause mortality following adjustment for smoking in FinnGen. The model was adjusted for sex (except for breast and prostate cancer), first 10 principal components of ancestry, genotyping batch and smoking at baseline. Figure S5: Associations of polygenic score for cardiorespiratory fitness with common non‐communicable diseases and all‐cause mortality following adjustment for BMI and smoking in FinnGen. The model was adjusted for sex (except for breast and prostate cancer), first 10 principal components of ancestry, genotyping batch, BMI and smoking at baseline. Figure S6: Cumulative incidence of lung cancer, type 2 diabetes, and all‐cause mortality in individuals with high (>90th percentile) and low (<10th percentile) polygenic score for cardiorespiratory fitness. Cumulative incidences are from Cox proportional hazards model and adjusted for sex, the first 10 principal components of ancestry, genotyping batch, BMI, and smoking status. Figure S7: Associations of polygenic score for cardiorespiratory fitness with common non‐communicable diseases and all‐cause mortality among never‐smokers in FinnGen. The model was adjusted for sex (except for breast and prostate cancer), first 10 principal components of ancestry, genotyping batch and BMI at baselin [file SMS-36-e70291-s001.pdf]

Supplemental Material

Evaluation of Genetics in the Association Between Cardiorespiratory Fitness and Health -
Insights from a Genome-Wide Polygenic Score for Cardiorespiratory Fitness in the FinnGen and HUNT cohorts

Joensuu L, Lukander V, Herranen P, Tynkkynen N, Kujala U, Lopéz-Bueno R, Nordeidet AN, Klevjer M, Øvretveit K, Wisløff U, Bye A, Ekelund U, Ollikainen M, FinnGen, Sillanpää E

Table S1. Endpoint data in FinnGen R12

Phenotype	ICD-10 codes	FinnGen label	Cases/N
**Cardiovascular diseases**			
All CVDs	I00-I99	I9_CVD	284,261/ 520,210
Ischemic heart diseases	I20-I25	I9_ISCHHEART	82,013 / 520,210
Hypertension	I10-I15, I67.4	I9_HYPTENS	158,906 / 520,210
Stroke	I60-I64, G45	C_STROKE	55,006 / 520,210
**Cancers**			
All cancers	C00-C97	ANY_CANC	120,922 / 520,210
Breast	C50	C3_BREAST_EXALLC	24,978 / 293,373
Prostate	C61	C3_PROSTATE_EXALLC	20,966 / 226,837
Colorectal	C18-20	C3_COLORECTAL_EXALLC	12187 / 520,210
Lung#	C34	C3_BRONCHUS_LUNG_EXALLC	9,966 / 520,210
**Pulmonary diseases**			
Chronic lower respiratory disease	J40-J47	J10_LOWCHRON	94,173 / 520,210
Asthma	J45-46	J10_ASTHMA	61,122 / 520,210
COPD	J43-44	J10_COPD	24,824 / 520,210
**Metabolic diseases**			
Type 2 diabetes	E11*	E4_DM2	92,194 / 520,210

#, Bronchus and lung, *Additionally medicine purchases for blood glucose lowering drugs, excluding insulin. Endpoint data can be browsed using FinnGen labels in https://risteys.finregistry.fi/.

Table S2. Descriptives of individuals in HUNT3 with measured cardiorespiratory fitness

Characteristics (N=3,655)	Mean (SD) or N (%)
Age (years)	49.0 (13.1)
Sex, female n (%)	1,920 (52.5%)
SES n (%)	
High	1,674 (45.8%)
Medium	1,825 (49.9%)
Low	156 (4.3%)
BMI (kg/m^2)	25.9 (3.5)
Smoking status n (%)	
Never	1,830 (50.1%)
Former	1,199 (32.8%)
Current	626 (17.1%)
Self-reported health n (%)	
Poor	9 (0.2%)
Not so good	358 (9.8%)
Good	2,229 (61.0%)
Very good	1,059 (29.0%)
Physical activity (MET h/week)	14.0 (9.1)
VO2peak (ml/kg/min)	40.3 (9.5)
VO2peak (ml/kg$^{0.75}$/min)	119.3 (28.4)

A complete case sample of HUNT3 with genotype, VO2max and covariate data. Values are presented as means (standard deviation) for continuous variables and N (percentages) for others. SES, socioeconomic status; BMI, body mass index; MET, metabolic equivalent of task; VO2peak, peak oxygen consumption

Table S3. Associations between different genetic scores and selected measures of CRF.

Genetic score	VO2peak ml/kg/min	p	Adj.R^2	VO2peak ml/kg^0.75/min	p	Adj.R^2
**PGS CRF**	0.44 (0.25 to 0.63)	**<0.001**	0.20%	1.34 (0.79 to 1.89)	**<0.001**	0.21%
**PGS CRF FFM**	0.19 (0.01 to 0.38)	**0.040**	0.03%	0.58 (0.03 to 1.13)	**0.040**	0.03%
**GRS CRF**	-0.03 (-0.22 to 0.16)	0.775	0.00%	0.01 (-0.54 to 0.56)	0.975	0.00%

Analyses were conducted using linear regression and interpreted as per one standard deviation increase in the genetic score. Values are unstandardized regression coefficients and 95% confidence intervals; All models were adjusted with first 10 principal components of ancestry, genotyping batch, age, sex, BMI, smoking status, socioeconomic status, self-reported health and self-reported physical activity (MET h/week); Adj.R^2, adjusted R^2 value for the genetic score in the model; PGS CRF, genome-wide polygenic score, constructed based on GWAS by Hanscombe et al. (2021) and the SBayesR method; PGS CRF FFM, genome-wide polygenic score constructed based on GWAS by Cai et al. (2023) and the SBayesR method; GRS CRF, genetic risk score, constructed based on favorable genetic variants reported by Cai et al. (2023) and their summarized effect sizes; Statistically significant associations highlighted with **bold**.

Table S4. A contingency table of the number of individuals in age- and sex-specific VO2peak and PGS CRF deciles.

VO2peak decile	PGS CRF decile 10	9	8	7	6	5	4	3	2	1
10	58	41	37	31	33	26	37	35	28	29
9	42	38	49	34	35	34	33	28	28	34
8	36	45	29	38	37	42	34	29	43	25
7	38	31	31	34	35	33	32	35	42	50
6	39	42	35	37	43	36	30	40	32	29
5	27	32	28	31	38	42	43	47	32	46
4	33	41	44	40	38	34	35	35	33	35
3	34	32	38	42	36	38	41	37	44	31
2	30	35	33	35	38	45	41	42	37	41
1	28	28	41	43	32	36	40	38	47	46

Individuals with high VO2peak and high PGS CRF are highlighted with blue ( ). Individuals with high VO2peak and low PGS CRF are highlighted with green ( ). Individuals with low VO2peak and high PGS CRF are highlighted with light blue ( ). Individuals with low VO2peak and low PGS CRF are highlighted with light green ( ). Group sizes were based on a-priori sample size calculation.

Table S5. Baseline characteristics of participants in the HUNT replication study

**Characteristics**	**All (n = 26,115)**	**Women (n = 13,687)**	**Men (n = 12,438)**
Age (y)	59.0 (14.5)	58.6 (15.2)	59.4 (13.4)
BMI (kg/m²)	27.8 (4.5)	27.7 (5.0)	27.9 (3.9)
Height (cm)	170.1 (9.3)	164.0 (6.3)	177.0 (6.6)
Weight (kg)	80.7 (15.4)	74.4 (14.2)	87.7 (13.6)
Smoking status n (%)			
Never	9,902 (37.9)	5,683 (41.5)	4,219 (33.9)
Former	9,611 (36.8)	4,339 (31.7)	5,272 (42.4)
Current	6,602 (25.3)	3,665 (26.8)	2,937 (23.6)

Values are means and (standard deviations) for continuous variables and n (%) for categorical/binary. BMI, body mass index.

Table S6. Associations between polygenic score for cardiorespiratory fitness and morbidity in the HUNT cohort

**Disease**	**Cases/N**	**Model 1 HR (95% CI)**	**Model 2 HR (95% CI)**	**Model 3 HR (95% CI)**	**Model 4 HR (95% CI)**
**Any CVD**	13,566/16,817	1.01 (0.99 to 1.03)	1.01 (0.99 to 1.03)	1.01 (0.99 to 1.03)	1.01 (0.99 to 1.03)
**Cancers**	2,448/26,115	1.02 (0.99 to 1.07)	1.02 (0.98 to 1.06)	1.02 (0.98 to 1.07)	1.02 (0.98 to 1.06)
**Pulmonary diseases**	3,185/25,084	0.97 (0.94 to 1.00)	0.97 (0.94 to 1.01)	0.98 (0.95 to 1.01)	0.98 (0.95 to 1.01)
**Type 2 diabetes**	2,663/25,410	**0.95 (0.92 to 0.99)**	0.99 (0.95 to 1.03)	**0.96 (0.92 to 0.99)**	0.99 (0.95 to 1.03)

Values are hazard ratios (HR) and 95% confidence intervals (CI) per one standard deviation increase in polygenic score for cardiorespiratory fitness (PGS CRF). We used Cox proportional hazards models and age as the time scale. Model 1: PGS CRF, ten first principal components of ancestry, sex, and genotyping batch; Model 2, additionally body mass index; Model 3, Model 1 and additionally smoking status (never, former, current), Model 4, Model 1 and additionally BMI and smoking;

Statistically significant associations highlighted with **bold**. No PGS CRF x Sex interactions were observed (all $p_{interactions}$ > 0.335) Mean follow-up was for cardiovascular diseases 8.1 years (min 0.1 years, max 18.1 years), for cancers 10.8 y (min 0.1 y, max 18.2 y), for pulmonary diseases 10.7 y (min 0.1, max 18.2 y) and for type 2 diabetes 10.7 y (min 0.1 y, max 18.2 y).

Table S7. Exploratory analyses between cardiorespiratory fitness and genetic liability groups with morbidity in the HUNT cohort.

Disease	Cases/N	HR (95% CI)	p-value
**Cardiovascular diseases**	376/452		
High V̇O2peak, High PGS CRF	92/112	Ref.	
High V̇O2peak, Low PGS CRF	62/75	0.85 (0.61 to 1.17)	0.314
Low V̇O2peak, High PGS CRF	102/120	1.05 (0.79 to 1.39)	0.760
Low V̇O2peak, Low PGS CRF	120/145	1.12 (0.85 to 1.47)	0.432
**Cancers**	64/541		
High V̇O2peak, High PGS CRF	20/133	Ref.	
High V̇O2peak, Low PGS CRF	14/98	0.83 (0.42 to 1.67)	0.606
Low V̇O2peak, High PGS CRF	13/138	0.59 (0.29 to 1.20)	0.144
Low V̇O2peak, Low PGS CRF	17/172	0.59 (0.31 to 1.13)	0.113
**Pulmonary diseases**	37/540		
High V̇O2peak, High PGS CRF	8/134	Ref.	
High V̇O2peak, Low PGS CRF	4/98	0.57 (0.17 to 1.90)	0.359
Low V̇O2peak, High PGS CRF	15/137	1.77 (0.75 to 4.20)	0.196
Low V̇O2peak, Low PGS CRF	10/171	0.96 (0.38 to 2.43)	0.931
**Type 2 diabetes**	29/529		
High V̇O2peak, High PGS CRF	2/134	Ref.	
High V̇O2peak, Low PGS CRF	1/97	0.59 (0.05 to 6.56)	0.670
Low V̇O2peak, High PGS CRF	11/134	**5.59 (1.23 to 25.25)**	**0.025**
Low V̇O2peak, Low PGS CRF	15/164	**6.20 to 1.41 to 27.16)**	**0.015**

Values are hazard ratios (HR) and 95% confidence intervals (CI) for belonging to different CRF and PGS group in comparison to High V̇O2peak, High PGS CRF group. We used Cox proportional hazards models and age as the time scale. Model included the group variable but no covariates due to limited statistical power. Mean follow-up was for cardiovascular diseases 8.4 years (min 0.1 years, max 17.3 years), cancers 10.7 y (min 0.1 y, max 17.4 y), pulmonary diseases 11.1 y (min 0.1, max 17.4 y) and type 2 diabetes 11.0 y (min 0.1 y, max 17.4 y).

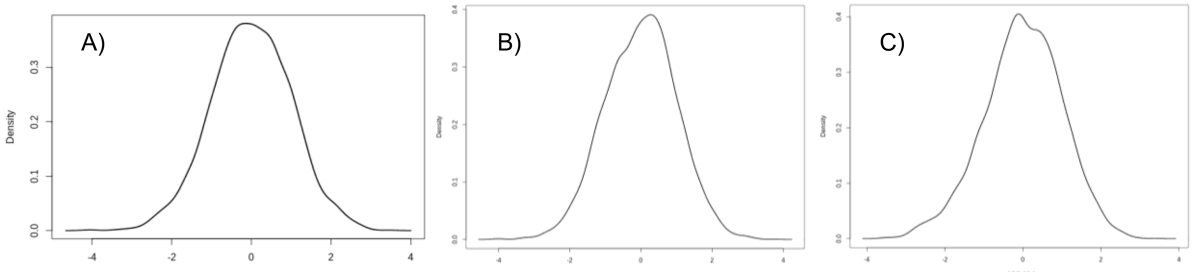

Figure S1. Density plots for three different genetic scores in the HUNT3; A) PGS CRF, B) PGS CRF FFM, and C) GRS CRF.

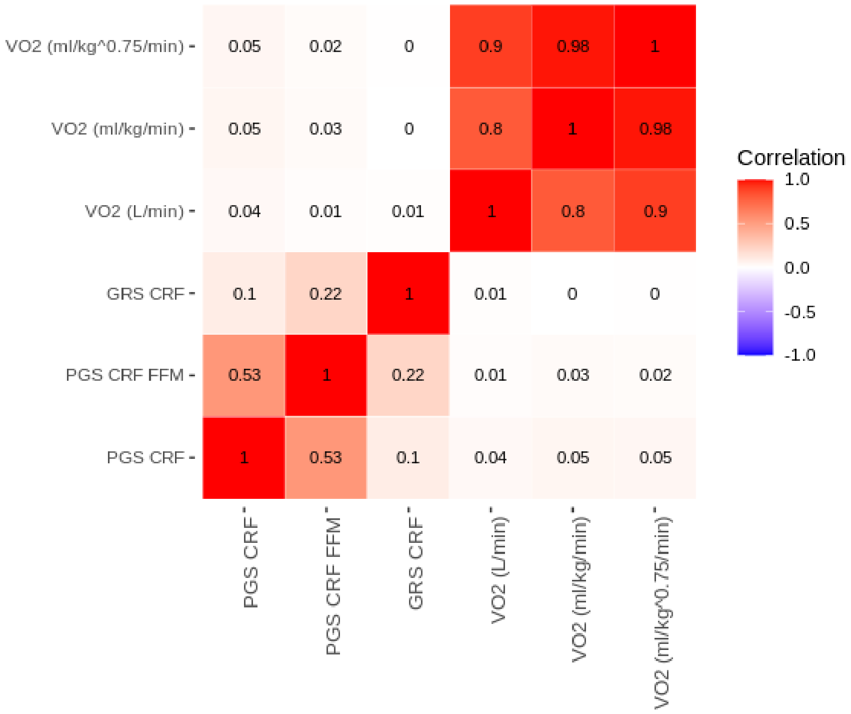

Figure S2. Correlation heatmap for PGS CRF, PGS CRF FFM and GRS CRF against selected markers of cardiorespiratory fitness. Values are Pearson correlation coefficients.

Disease	Cases/N	Incidence/10,000 PY	HR (95% CI)	P-value	FDR P-value
**Cardiovascular diseases**					
Any CVD	45,504 / 156,481	354.86	0.99 (0.98 to 1.00)	0.120	0.210
Ischemic heart diseases	25,861 / 236,274	121.79	0.99 (0.97 to 1.00)	0.029	0.068
Hypertensive diseases	31,533 / 208,759	171.60	1.00 (0.99 to 1.01)	0.660	0.670
Stroke	18,247 / 199,684	79.70	0.99 (0.97 to 1.00)	0.080	0.160
**Cancer**					
Any cancer	29,570 / 227,855	138.59	1.00 (0.98 to 1.01)	0.450	0.525
Breast	3,219 / 110,518	13.39	1.02 (0.98 to 1.05)	0.360	0.458
Prostate	6,432 / 95,238	26.75	1.01 (0.98 to 1.03)	0.670	0.670
Colorectal	3,230 / 199,335	13.23	0.98 (0.95 to 1.02)	0.290	0.406
Lung (Bronchus and lung)	5,427 / 201,532	22.12	0.94 (0.92 to 0.97)	<0.001	<0.001
**Pulmonary diseases**					
Chronic lower respiratory disease	16,871 / 228,716	78.97	0.98 (0.97 to 1.00)	0.017	0.048
Asthma	9,053 / 147,245	39.90	0.99 (0.97 to 1.01)	0.230	0.358
COPD	10,553 / 222,398	44.35	0.97 (0.95 to 0.99)	0.001	0.004
**Metabolic diseases**					
Type 2 diabetes	21,347 / 233,842	99.71	0.97 (0.96 to 0.99)	<0.001	0.001
**Mortality**					
All-cause	45,036 / 262,048	182.50	0.98 (0.97 to 0.99)	<0.001	0.001

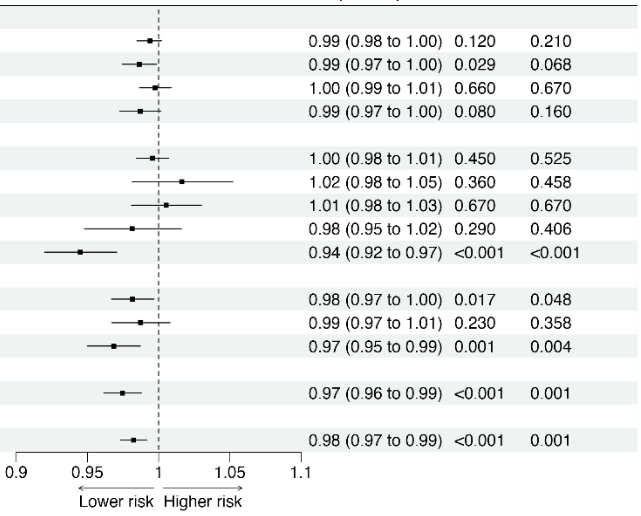

Figure S3. Associations of polygenic score for cardiorespiratory fitness with common non-communicable diseases and all-cause mortality following adjustment for BMI in FinnGen. The model was adjusted for sex (except for breast and prostate cancer), first 10 principal components of ancestry, genotyping batch and BMI at baseline.

Disease	Cases/N	Incidence/10,000 PY	HR (95% CI)	P-value	FDR P-value
**Cardiovascular diseases**					
Any CVD	45,504 / 156,481	354.86	0.99 (0.98 to 1.00)	0.012	0.034
Ischemic heart diseases	25,861 / 236,274	121.79	0.98 (0.97 to 1.00)	0.010	0.034
Hypertensive diseases	31,533 / 208,759	171.60	0.99 (0.98 to 1.00)	0.016	0.037
Stroke	18,247 / 199,684	79.70	0.99 (0.97 to 1.00)	0.050	0.078
**Cancer**					
Any cancer	29,570 / 227,855	138.59	1.00 (0.99 to 1.01)	0.623	0.630
Breast	3,219 / 110,518	13.39	1.02 (0.98 to 1.05)	0.320	0.373
Prostate	6,432 / 95,238	26.75	1.01 (0.98 to 1.03)	0.630	0.630
Colorectal	3,230 / 199,335	13.23	0.98 (0.95 to 1.01)	0.265	0.338
Lung (Bronchus and lung)	5,427 / 201,532	22.12	0.95 (0.93 to 0.98)	<0.001	0.003
**Pulmonary diseases**					
Chronic lower respiratory disease	16,871 / 228,716	78.97	0.98 (0.97 to 1.00)	0.046	0.078
Asthma	9,053 / 147,245	39.90	0.98 (0.96 to 1.00)	0.093	0.130
COPD	10,553 / 222,398	44.35	0.98 (0.96 to 1.00)	0.023	0.047
**Metabolic diseases**					
Type 2 diabetes	21,347 / 233,842	99.71	0.96 (0.95 to 0.97)	<0.001	<0.001
**Mortality**					
All-cause	45,036 / 262,048	182.50	0.98 (0.98 to 0.99)	0.001	0.005

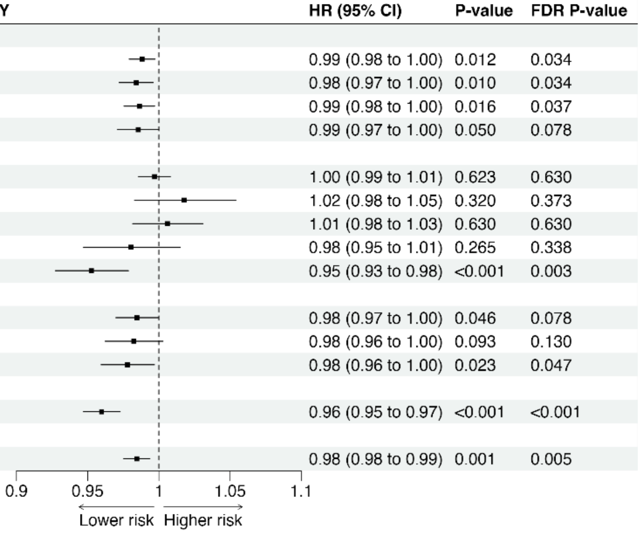

Figure S4. Associations of polygenic score for cardiorespiratory fitness with common non-communicable diseases and all-cause mortality following adjustment for smoking in FinnGen. The model was adjusted for sex (except for breast and prostate cancer), first 10 principal components of ancestry, genotyping batch and smoking at baseline.

Disease	Cases/N	Incidence/10,000 PY	HR (95% CI)	P-value	FDR P-value
**Cardiovascular diseases**					
Any CVD	45,504 / 156,481	354.86	0.99 (0.98 to 1.00)	0.200	0.350
Ischemic heart diseases	25,861 / 236,274	121.79	0.99 (0.98 to 1.00)	0.054	0.151
Hypertensive diseases	31,533 / 208,759	171.60	1.00 (0.99 to 1.01)	0.790	0.790
Stroke	18,247 / 199,684	79.70	0.99 (0.97 to 1.00)	0.140	0.280
**Cancer**					
Any cancer	29,570 / 227,855	138.59	1.00 (0.99 to 1.01)	0.580	0.677
Breast	3,219 / 110,518	13.39	1.02 (0.98 to 1.05)	0.360	0.458
Prostate	6,432 / 95,238	26.75	1.01 (0.98 to 1.03)	0.660	0.711
Colorectal	3,230 / 199,335	13.23	0.98 (0.95 to 1.02)	0.310	0.458
Lung (Bronchus and lung)	5,427 / 201,532	22.12	0.95 (0.93 to 0.98)	<0.001	0.003
**Pulmonary diseases**					
Chronic lower respiratory disease	16,871 / 228,716	78.97	0.99 (0.97 to 1.00)	0.082	0.191
Asthma	9,053 / 147,245	39.90	0.99 (0.97 to 1.01)	0.330	0.458
COPD	10,553 / 222,398	44.35	0.98 (0.96 to 1.00)	0.019	0.067
**Metabolic diseases**					
Type 2 diabetes	21,347 / 233,842	99.71	0.98 (0.96 to 0.99)	<0.001	0.003
**Mortality**					
All-cause	45,036 / 262,048	182.50	0.98 (0.98 to 0.99)	0.001	0.005

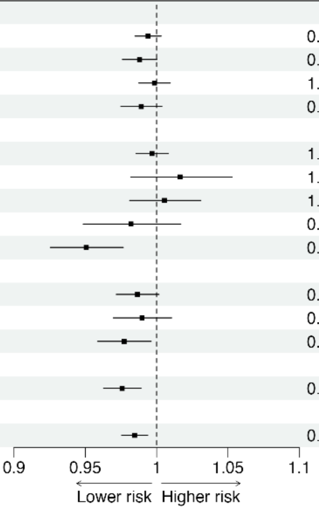

Figure S5. Associations of polygenic score for cardiorespiratory fitness with common non-communicable diseases and all-cause mortality following adjustment for BMI and smoking in FinnGen. The model was adjusted for sex (except for breast and prostate cancer), first 10 principal components of ancestry, genotyping batch, BMI and smoking at baseline.

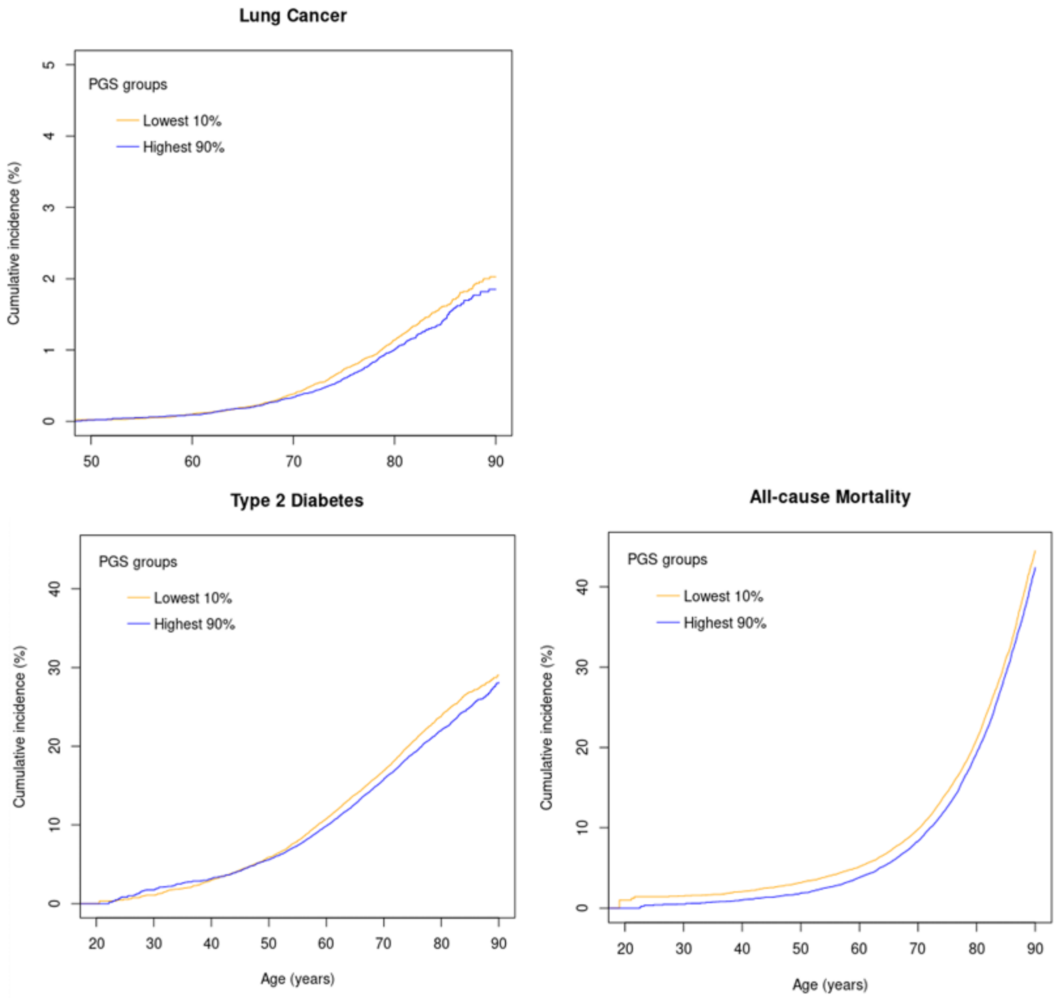

Figure S6. Cumulative incidence of lung cancer, type 2 diabetes, and all-cause mortality in individuals with high (>90th percentile) and low (<10th percentile) polygenic score for cardiorespiratory fitness. Cumulative incidences are from Cox proportional hazards model and adjusted for sex, the first 10 principal components of ancestry, genotyping batch, BMI, and smoking status.

Disease	Cases/N	Incidence/10,000 PY	HR (95% CI)	P-value	FDR P-value
**Cardiovascular diseases**					
Any CVD	18,045 / 78,460	354.86	0.99 (0.98 to 1.00)	0.170	0.862
Ischemic heart diseases	8,662 / 119,262	121.79	0.99 (0.97 to 1.01)	0.440	0.862
Hypertensive diseases	13,170 / 103,988	171.60	1.00 (0.98 to 1.02)	0.980	0.980
Stroke	6,910 / 104,939	79.70	1.00 (0.97 to 1.02)	0.800	0.862
**Cancer**					
Any cancer	10,357 / 111,771	138.59	1.01 (0.99 to 1.03)	0.200	0.862
Breast	2,005 / 66,448	13.39	1.01 (0.97 to 1.06)	0.640	0.862
Prostate	1,726 / 37,588	26.75	1.02 (0.98 to 1.07)	0.350	0.862
Colorectal	1,246 / 101,551	13.23	0.98 (0.92 to 1.03)	0.380	0.862
Lung (Bronchus and lung)	443 / 100,748	22.12	1.01 (0.92 to 1.11)	0.760	0.862
**Pulmonary diseases**					
Chronic lower respiratory disease	4,063 / 115,209	78.97	1.00 (0.96 to 1.03)	0.770	0.862
Asthma	3,471 / 78,970	39.90	1.00 (0.96 to 1.03)	0.790	0.862
COPD	509 / 111,655	44.35	0.96 (0.88 to 1.05)	0.400	0.862
**Metabolic diseases**					
Type 2 diabetes	7,974 / 117,275	99.71	0.98 (0.95 to 1.00)	0.028	0.392
**Mortality**					
All-cause	12,183 / 130,159	182.50	1.00 (0.98 to 1.02)	0.770	0.862

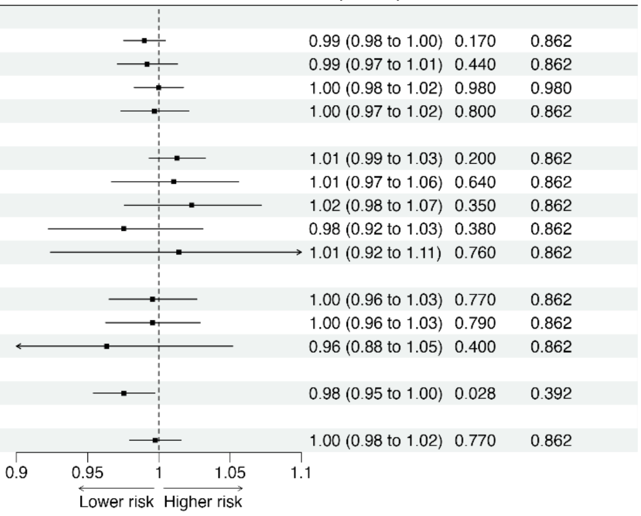

Figure S7. Associations of polygenic score for cardiorespiratory fitness with common non-communicable diseases and all-cause mortality among never-smokers in FinnGen. The model was adjusted for sex (except for breast and prostate cancer), first 10 principal components of ancestry, genotyping batch and BMI at baseline.

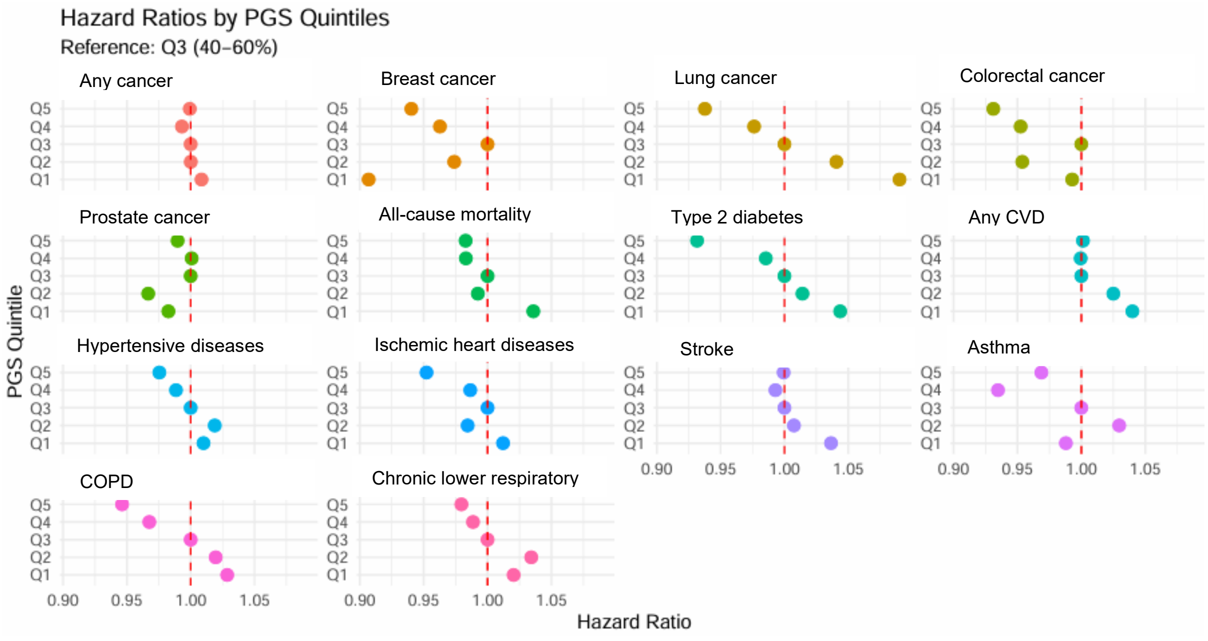

Figure S8. Hazard ratios by PGS CRF quintiles in FinnGen. The model was adjusted for sex (except for breast and prostate cancer), first 10 principal components of ancestry, and genotyping batch. The population was divided into five equal groups (20 % each), ranging from Q1 (lowest genetic score) to Q5 (highest genetic score). Q3 is the middle quintile (40th to 60th percentile), and set as the reference category (HR = 1.00).

FinnGen Authors

Full Name	Affiliation	Role 1	Role 2
Aarno Palotie	Institute for Molecular Medicine Finland (FIMM), HiLIFE, University of Helsinki, Helsinki, Finland; Broad Institute of MIT and Harvard; Massachusetts General Hospital, Boston, MA, United States	Steering Committee	Steering Committee
Mark Daly	Institute for Molecular Medicine Finland (FIMM), HiLIFE, University of Helsinki, Helsinki, Finland; Broad Institute of MIT and Harvard; Massachusetts General Hospital, Boston, MA, United States	Steering Committee	Steering Committee
Bridget Riley-Gills	Abbvie, Chicago, IL, United States	Steering Committee	Pharmaceutical companies
Howard Jacob	Abbvie, Chicago, IL, United States	Steering Committee	Pharmaceutical companies
Coralie Viollet	Astra Zeneca, Cambridge, United Kingdom	Steering Committee	Pharmaceutical companies
Slavé Petrovski	Astra Zeneca, Cambridge, United Kingdom	Steering Committee	Pharmaceutical companies
Alix Berton	Bayer AG, Leverkusen, Germany	Steering Committee	Pharmaceutical companies
Santha Ramakrishnan	Bayer AG, Leverkusen, Germany	Steering Committee	Pharmaceutical companies
Ellen Tsai	Biogen, Cambridge, MA, United States	Steering Committee	Pharmaceutical companies
Zhihao Ding	Boehringer Ingelheim, Ingelheim am Rhein, Germany	Steering Committee	Pharmaceutical companies
Emily Holzinger	Bristol Myers Squibb, New York, NY, United States	Steering Committee	Pharmaceutical companies
Robert Plenge	Bristol Myers Squibb, New York, NY, United States	Steering Committee	Pharmaceutical companies
Joseph Maranville	Bristol Myers Squibb, New York, NY, United States	Steering Committee	Pharmaceutical companies
Mark McCarthy	Genentech, San Francisco, CA, United States	Steering Committee	Pharmaceutical companies
Rion Pendergrass	Genentech, San Francisco, CA, United States	Steering Committee	Pharmaceutical companies
Jonathan Davitte	GlaxoSmithKline, Collegeville, PA, United States	Steering Committee	Pharmaceutical companies
Simonne Longerich	Merck, Kenilworth, NJ, United States	Steering Committee	Pharmaceutical companies
Anders Mälarstig	Pfizer, New York, NY, United States	Steering Committee	Pharmaceutical companies
Anna Vlahiotis	Pfizer, New York, NY, United States	Steering Committee	Pharmaceutical companies
Katherine Klinger	Translational Sciences, Sanofi R&D, Framingham, MA, USA	Steering Committee	Pharmaceutical companies
Clement Chatelain	Translational Sciences, Sanofi R&D, Framingham, MA, USA	Steering Committee	Pharmaceutical companies
Jorg Blankenstein	Translational Sciences, Sanofi R&D, Framingham, MA, USA	Steering Committee	Pharmaceutical companies
Karol Estrada	Maze Therapeutics, San Francisco, CA, United States	Steering Committee	Pharmaceutical companies
Robert Graham	Maze Therapeutics, San Francisco, CA, United States	Steering Committee	Pharmaceutical companies
Dawn Waterworth	Johnson & Johnson Innovative Medicine, Spring House, PA, United States	Steering Committee	Pharmaceutical companies
Chris O´Donnell	Novartis Institutes for BioMedical Research, Cambridge, MA, United States	Steering Committee	Pharmaceutical companies
Nicole Renaud	Novartis Institutes for BioMedical Research, Cambridge, MA, United States	Steering Committee	Pharmaceutical companies
Tomi P. Mäkelä	HiLIFE, University of Helsinki, Finland	Steering Committee	University of Helsinki & Biobanks
Jaakko Kaprio	Institute for Molecular Medicine Finland (FIMM), HiLIFE, University of Helsinki, Helsinki, Finland	Steering Committee	University of Helsinki & Biobanks
Minna Ruddock	Arctic biobank / University of Oulu, Oulu, Finland	Steering Committee	University of Helsinki & Biobanks
Petri Virolainen	Auria Biobank / University of Turku / Wellbeing Services County of Southwest Finland, Turku, Finland	Steering Committee	University of Helsinki & Biobanks
Antti Hakanen	Auria Biobank / University of Turku / Wellbeing Services County of Southwest Finland, Turku, Finland	Steering Committee	University of Helsinki & Biobanks
Terhi Kilpi	THL Biobank / Finnish Institute for Health and Welfare (THL), Helsinki, Finland	Steering Committee	University of Helsinki & Biobanks
Markus Perola	THL Biobank / Finnish Institute for Health and Welfare (THL), Helsinki, Finland	Steering Committee	University of Helsinki & Biobanks

Jukka Partanen	Finnish Red Cross Blood Service / Finnish Hematology Registry and Clinical Biobank, Helsinki, Finland	Steering Committee	**University of Helsinki & Biobanks**
Taneli Raivio	Helsinki Biobank / Helsinki University and Hospital District of Helsinki and Uusimaa, Helsinki	Steering Committee	**University of Helsinki & Biobanks**
Raisa Serpi	Northern Finland Biobank Borealis / University of Oulu / Wellbeing services county of North Ostrobothnia, Oulu, Finland	Steering Committee	**University of Helsinki & Biobanks**
Teija Kekonen	Northern Finland Biobank Borealis / University of Oulu / Wellbeing services county of North Ostrobothnia, Oulu, Finland	Steering Committee	**University of Helsinki & Biobanks**
Kati Kristiansson	Finnish Clinical Biobank Tampere / University of Tampere / Wellbeing Services County of Pirkanmaa, Tampere, Finland	Steering Committee	**University of Helsinki & Biobanks**
Veli-Matti Kosma	Biobank of Eastern Finland / University of Eastern Finland / Wellbeing services county of North Savo, Kuopio, Finland	Steering Committee	**University of Helsinki & Biobanks**
Jari Laukkanen	Central Finland Biobank / University of Jyväskylä / Wellbeing Services County of Central Finland, Jyväskylä, Finland	Steering Committee	**University of Helsinki & Biobanks**
Tom Southerington	Finnish Biobank Cooperative - FINBB	Steering Committee	**University of Helsinki & Biobanks**
Outi Tuovila	Business Finland, Helsinki, Finland	Steering Committee	**Other Experts/ Non-Voting Members**
Jeffrey Waring	Abbvie, Chicago, IL, United States	Scientific Committee	**Pharmaceutical companies**
Bridget Riley-Gillis	Abbvie, Chicago, IL, United States	Scientific Committee	**Pharmaceutical companies**
Fedik Rahimov	Abbvie, Chicago, IL, United States	Scientific Committee	**Pharmaceutical companies**
Ioanna Tachmazidou	Astra Zeneca, Cambridge, United Kingdom	Scientific Committee	**Pharmaceutical companies**
Alix Berton	Bayer AG, Leverkusen, Germany	Scientific Committee	**Pharmaceutical companies**
Santha Ramakrishnan	Bayer AG, Leverkusen, Germany	Scientific Committee	**Pharmaceutical companies**
Ellen Tsai	Biogen, Cambridge, MA, United States	Scientific Committee	**Pharmaceutical companies**
Zhihao Ding	Boehringer Ingelheim, Ingelheim am Rhein, Germany	Scientific Committee	**Pharmaceutical companies**
Marc Jung	Boehringer Ingelheim, Ingelheim am Rhein, Germany	Scientific Committee	**Pharmaceutical companies**
Hanati Tuoken	Boehringer Ingelheim, Ingelheim am Rhein, Germany	Scientific Committee	**Pharmaceutical companies**
Shameek Biswas	Bristol Myers Squibb, New York, NY, United States	Scientific Committee	**Pharmaceutical companies**
Benjamin Sun	Bristol Myers Squibb, New York, NY, United States	Scientific Committee	**Pharmaceutical companies**
Rion Pendergrass	Genentech, San Francisco, CA, United States	Scientific Committee	**Pharmaceutical companies**
Jonathan Davitte	GlaxoSmithKline, Collegeville, PA, United States	Scientific Committee	**Pharmaceutical companies**
Neha Raghavan	Merck, Kenilworth, NJ, United States	Scientific Committee	**Pharmaceutical companies**
Adriana Huertas-Vazquez	Merck, Kenilworth, NJ, United States	Scientific Committee	**Pharmaceutical companies**
Jae-Hoon Sul	Merck, Kenilworth, NJ, United States	Scientific Committee	**Pharmaceutical companies**
Anders Mälarstig	Pfizer, New York, NY, United States	Scientific Committee	**Pharmaceutical companies**
Xinli Hu	Pfizer, New York, NY, United States	Scientific Committee	**Pharmaceutical companies**
Åsa Hedman	Pfizer, New York, NY, United States	Scientific Committee	**Pharmaceutical companies**
Katherine Klinger	Translational Sciences, Sanofi R&D, Framingham, MA, USA	Scientific Committee	**Pharmaceutical companies**
Robert Graham	Maze Therapeutics, San Francisco, CA, United States	Scientific Committee	**Pharmaceutical companies**
Dawn Waterworth	Johnson & Johnson Innovative Medicine, Spring House, PA, United States	Scientific Committee	**Pharmaceutical companies**
Nicole Renaud	Novartis Institutes for BioMedical Research, Cambridge, MA, United States	Scientific Committee	**Pharmaceutical companies**
Ma´en Obeidat	Novartis Institutes for BioMedical Research, Cambridge, MA, United States	Scientific Committee	**Pharmaceutical companies**
Jonathan Chung	Novartis Institutes for BioMedical Research, Cambridge, MA, United States	Scientific Committee	**Pharmaceutical companies**

Jonas Zierer	Novartis Institutes for BioMedical Research, Cambridge, MA, United States	**Scientific Committee**	**Pharmaceutical companies**
Mari Niemi	Novartis Institutes for BioMedical Research, Cambridge, MA, United States	**Scientific Committee**	**Pharmaceutical companies**
Samuli Ripatti	Institute for Molecular Medicine Finland (FIMM), HiLIFE, University of Helsinki, Helsinki, Finland	**Scientific Committee**	**University of Helsinki & Biobanks**
Johanna Schleutker	Auria Biobank / University of Turku / Wellbeing Services County of Southwest Finland, Turku, Finland	**Scientific Committee**	**University of Helsinki & Biobanks**
Markus Perola	THL Biobank / Finnish Institute for Health and Welfare (THL), Helsinki, Finland	**Scientific Committee**	**University of Helsinki & Biobanks**
Mikko Arvas	Finnish Red Cross Blood Service / Finnish Hematology Registry and Clinical Biobank, Helsinki, Finland	**Scientific Committee**	**University of Helsinki & Biobanks**
Olli Carpén	Helsinki Biobank / Helsinki University and Hospital District of Helsinki and Uusimaa, Helsinki	**Scientific Committee**	**University of Helsinki & Biobanks**
Reetta Hinttala	Northern Finland Biobank Borealis / University of Oulu / Wellbeing services county of North Ostrobothnia, Oulu, Finland	**Scientific Committee**	**University of Helsinki & Biobanks**
Johannes Kettunen	Northern Finland Biobank Borealis / University of Oulu / Wellbeing services county of North Ostrobothnia, Oulu, Finland	**Scientific Committee**	**University of Helsinki & Biobanks**
Arto Mannermaa	Biobank of Eastern Finland / University of Eastern Finland / Wellbeing services county of North Savo, Kuopio, Finland	**Scientific Committee**	**University of Helsinki & Biobanks**
Katriina Aalto-Setälä	Faculty of Medicine and Health Technology, Tampere University, Tampere, Finland	**Scientific Committee**	**University of Helsinki & Biobanks**
Mika Kähönen	Finnish Clinical Biobank Tampere / University of Tampere / Wellbeing Services County of Pirkanmaa, Tampere, Finland	**Scientific Committee**	**University of Helsinki & Biobanks**
Jari Laukkanen	Central Finland Biobank / University of Jyväskylä / Wellbeing Services County of Central Finland, Jyväskylä, Finland	**Scientific Committee**	**University of Helsinki & Biobanks**
Johanna Mäkelä	FINBB - Finnish biobank cooperative	**Scientific Committee**	**University of Helsinki & Biobanks**
Lila Kallio	Auria Biobank / University of Turku / Wellbeing Services County of Southwest Finland, Turku, Finland	**Biobank directors**	**Biobank directors**
Tiina Wahlfors	THL Biobank / Finnish Institute for Health and Welfare (THL), Helsinki, Finland	**Biobank directors**	**Biobank directors**
Jukka Partanen	Finnish Red Cross Blood Service / Finnish Hematology Registry and Clinical Biobank, Helsinki, Finland	**Biobank directors**	**Biobank directors**
Eero Punkka	Helsinki Biobank / Helsinki University and Hospital District of Helsinki and Uusimaa, Helsinki	**Biobank directors**	**Biobank directors**
Raisa Serpi	Northern Finland Biobank Borealis / University of Oulu / Wellbeing services county of North Ostrobothnia, Oulu, Finland	**Biobank directors**	**Biobank directors**
Sanna Siltanen	Finnish Clinical Biobank Tampere / University of Tampere / Wellbeing Services County of Pirkanmaa, Tampere, Finland	**Biobank directors**	**Biobank directors**
Veli-Matti Kosma	Biobank of Eastern Finland / University of Eastern Finland / Wellbeing services county of North Savo, Kuopio, Finland	**Biobank directors**	**Biobank directors**
Tiina Jokela	Central Finland Biobank / University of Jyväskylä / Wellbeing Services County of Central Finland, Jyväskylä, Finland	**Biobank directors**	**Biobank directors**
Anu Jalanko	Institute for Molecular Medicine Finland (FIMM), HiLIFE, University of Helsinki, Helsinki, Finland	**FinnGen Teams**	**Administration**
Auli Toivola	Institute for Molecular Medicine Finland (FIMM), HiLIFE, University of Helsinki, Helsinki, Finland	**FinnGen Teams**	**Administration**
Denise Öller	Institute for Molecular Medicine Finland (FIMM), HiLIFE, University of Helsinki, Helsinki, Finland	**FinnGen Teams**	**Administration**
Helen Cooper	Institute for Molecular Medicine Finland (FIMM), HiLIFE, University of Helsinki, Helsinki, Finland	**FinnGen Teams**	**Administration**
Mervi Aavikko	Institute for Molecular Medicine Finland (FIMM), HiLIFE, University of Helsinki, Helsinki, Finland	**FinnGen Teams**	**Administration**
Risto Kajanne	Institute for Molecular Medicine Finland (FIMM), HiLIFE, University of Helsinki, Helsinki, Finland	**FinnGen Teams**	**Administration**
Rodos Rodosthenous	Institute for Molecular Medicine Finland (FIMM), HiLIFE, University of Helsinki, Helsinki, Finland	**FinnGen Teams**	**Administration**
Sofia Kuitunen	University of Helsinki, Helsinki, Finland	**FinnGen Teams**	**Administration**
Tarja Laitinen	Institute for Molecular Medicine Finland (FIMM), HiLIFE, University of Helsinki, Helsinki, Finland	**FinnGen Teams**	**Administration**

Arto Lehisto	Institute for Molecular Medicine Finland (FIMM), HiLIFE, University of Helsinki, Helsinki, Finland	FinnGen Teams	**Analysis**
Hafiz Sikandar	Institute for Molecular Medicine Finland (FIMM), HiLIFE, University of Helsinki, Helsinki, Finland	FinnGen Teams	**Analysis**
Juha Karjalainen	Institute for Molecular Medicine Finland (FIMM), HiLIFE, University of Helsinki, Helsinki, Finland	FinnGen Teams	**Analysis**
Juha Mehtonen	Institute for Molecular Medicine Finland (FIMM), HiLIFE, University of Helsinki, Helsinki, Finland	FinnGen Teams	**Analysis**
Masahiro Kanai	Broad Institute, Cambridge, MA, United States	FinnGen Teams	**Analysis**
Mitja Kurki	Institute for Molecular Medicine Finland (FIMM), HiLIFE, University of Helsinki, Helsinki, Finland; Broad Institute, Cambridge, MA, United States	FinnGen Teams	**Analysis**
Mutaamba Maasha	Broad Institute, Cambridge, MA, United States	FinnGen Teams	**Analysis**
Pietro Della Briotta Parolo	Institute for Molecular Medicine Finland (FIMM), HiLIFE, University of Helsinki, Helsinki, Finland	FinnGen Teams	**Analysis**
Samuel Jones	Institute for Molecular Medicine Finland (FIMM), HiLIFE, University of Helsinki, Helsinki, Finland	FinnGen Teams	**Analysis**
Sanni Ruotsalainen	Institute for Molecular Medicine Finland (FIMM), HiLIFE, University of Helsinki, Helsinki, Finland	FinnGen Teams	**Analysis**
Susanna Lemmelä	Institute for Molecular Medicine Finland (FIMM), HiLIFE, University of Helsinki, Helsinki, Finland	FinnGen Teams	**Analysis**
Wei Zhou	Broad Institute, Cambridge, MA, United States	FinnGen Teams	**Analysis**
Aki Havulinna	Finnish Institute for Health and Welfare (THL), Helsinki, Finland	FinnGen Teams	**Clinical Endpoint Development**
L. Elisa Lahtela	Institute for Molecular Medicine Finland (FIMM), HiLIFE, University of Helsinki, Helsinki, Finland	FinnGen Teams	**Clinical Endpoint Development**
Mari Kaunisto	Institute for Molecular Medicine Finland (FIMM), HiLIFE, University of Helsinki, Helsinki, Finland	FinnGen Teams	**Communication**
Awaisa Ghazal	Institute for Molecular Medicine Finland (FIMM), HiLIFE, University of Helsinki, Helsinki, Finland	FinnGen Teams	**E-Science**
Elina Kilpeläinen	Institute for Molecular Medicine Finland (FIMM), HiLIFE, University of Helsinki, Helsinki, Finland	FinnGen Teams	**E-Science**
Jaska Uimonen	Institute for Molecular Medicine Finland (FIMM), HiLIFE, University of Helsinki, Helsinki, Finland	FinnGen Teams	**E-Science**
Oluwaseun Alexander Dada	Institute for Molecular Medicine Finland (FIMM), HiLIFE, University of Helsinki, Helsinki, Finland	FinnGen Teams	**E-Science**
Rigbe Weldatsadik	Institute for Molecular Medicine Finland (FIMM), HiLIFE, University of Helsinki, Helsinki, Finland	FinnGen Teams	**E-Science**
Sanni Ruotsalainen	Institute for Molecular Medicine Finland (FIMM), HiLIFE, University of Helsinki, Helsinki, Finland	FinnGen Teams	**E-Science**
Tianduanyi Wang	Institute for Molecular Medicine Finland (FIMM), HiLIFE, University of Helsinki, Helsinki, Finland	FinnGen Teams	**E-Science**
Timo P. Sipilä	Institute for Molecular Medicine Finland (FIMM), HiLIFE, University of Helsinki, Helsinki, Finland	FinnGen Teams	**E-Science**
Kati Donner	Institute for Molecular Medicine Finland (FIMM), HiLIFE, University of Helsinki, Helsinki, Finland	FinnGen Teams	**Genotyping**
Anu Loukola	Helsinki Biobank / Helsinki University and Hospital District of Helsinki and Uusimaa, Helsinki	FinnGen Teams	**Sample Collection Coordination**
Päivi Ingalsuo	THL Biobank / Finnish Institute for Health and Welfare (THL), Helsinki, Finland	FinnGen Teams	**Sample Logistics**
Arto Pietilä	THL Biobank / Finnish Institute for Health and Welfare (THL), Helsinki, Finland	FinnGen Teams	**Registry Data Operations**
Sami Koskelainen	THL Biobank / Finnish Institute for Health and Welfare (THL), Helsinki, Finland	FinnGen Teams	**Registry Data Operations**
Susanna Lemmelä	Institute for Molecular Medicine Finland (FIMM), HiLIFE, University of Helsinki, Helsinki, Finland	FinnGen Teams	**Registry Data Operations**
Teemu Paajanen	THL Biobank / Finnish Institute for Health and Welfare (THL), Helsinki, Finland	FinnGen Teams	**Registry Data Operations**
Tero Hiekkalinna	THL Biobank / Finnish Institute for Health and Welfare (THL), Helsinki, Finland	FinnGen Teams	**Registry Data Operations**
Priit Palta	Institute for Molecular Medicine Finland (FIMM), HiLIFE, University of Helsinki, Helsinki, Finland	FinnGen Teams	**Sequencing Informatics**
Dawit A. Yohannes	Institute for Molecular Medicine Finland (FIMM), HiLIFE, University of Helsinki, Helsinki, Finland	FinnGen Teams	**Phenotype team**
Harri Siirtola	University of Tampere, Tampere, Finland	FinnGen Teams	**Phenotype team**
Javier Gracia-Tabuenca	University of Tampere, Tampere, Finland	FinnGen Teams	**Phenotype team**
Marika Kaakinen	Institute for Molecular Medicine Finland (FIMM), HiLIFE, University of Helsinki, Helsinki, Finland	FinnGen Teams	**Phenotype team**
Mary Pat Reeve	Institute for Molecular Medicine Finland (FIMM), HiLIFE, University of Helsinki, Helsinki, Finland	FinnGen Teams	**Phenotype team**
Shanmukha Sampath Padmanabhuni	Institute for Molecular Medicine Finland (FIMM), HiLIFE, University of Helsinki, Helsinki, Finland	FinnGen Teams	**Phenotype team**

Shuang Luo	Institute for Molecular Medicine Finland (FIMM), HiLIFE, University of Helsinki, Helsinki, Finland	**FinnGen Teams**	**Phenotype team**
Vincent Llorens	Institute for Molecular Medicine Finland (FIMM), HiLIFE, University of Helsinki, Helsinki, Finland	**FinnGen Teams**	**Phenotype team**
Iina Laak	Institute for Molecular Medicine Finland (FIMM), HiLIFE, University of Helsinki, Helsinki, Finland	**FinnGen Teams**	**Data protection officer**
Jaakko Tyrmi	University of Oulu, Oulu, Finland / University of Tampere, Tampere, Finland	**FinnGen Teams**	**FinnGen local support person**
Janne Isojärvi	University of Turku, Turku, Finland	**FinnGen Teams**	**FinnGen local support person**
Tero Sievänen	University of Eastern Finland, Kuopio, Finland	**FinnGen Teams**	**FinnGen local support person**
Timo Pohjonen	University of Jyväskylä, Jyväskylä, Finland	**FinnGen Teams**	**FinnGen local support person**
Vidal Fey	University of Tampere, Tampere, Finland	**FinnGen Teams**	**FinnGen local support person**
Johanna Mäkelä	Finnish Biobank Cooperative - FINBB	**FinnGen Teams**	**FINBB - Finnish biobank cooperative**
Pauli Wihuri	Finnish Biobank Cooperative - FINBB	**FinnGen Teams**	**FINBB - Finnish biobank cooperative**
Tom Southerington	Finnish Biobank Cooperative - FINBB	**FinnGen Teams**	**FINBB - Finnish biobank cooperative**
Meri Lähteenmäki	Finnish Biobank Cooperative - FINBB	**FinnGen Teams**	**FINBB - Finnish biobank cooperative**
Reetta Kälviäinen	Wellbeing services county of North Savo, Kuopio, Finland	**Clinical Groups (FinnGen phases 1&2)**	**Neurology Group**
Valtteri Julkunen	Wellbeing services county of North Savo, Kuopio, Finland	**Clinical Groups (FinnGen phases 1&2)**	**Neurology Group**
Hilkka Soininen	Wellbeing services county of North Savo, Kuopio, Finland	**Clinical Groups (FinnGen phases 1&2)**	**Neurology Group**
Anne Remes	Wellbeing services county of North Ostrobothnia, Oulu, Finland	**Clinical Groups (FinnGen phases 1&2)**	**Neurology Group**
Mikko Hiltunen	University of Eastern Finland, Kuopio, Finland	**Clinical Groups (FinnGen phases 1&2)**	**Neurology Group**
Jukka Peltola	Wellbeing Services County of Pirkanmaa, Tampere, Finland	**Clinical Groups (FinnGen phases 1&2)**	**Neurology Group**
Minna Raivio	Hospital District of Helsinki and Uusimaa, Helsinki, Finland	**Clinical Groups (FinnGen phases 1&2)**	**Neurology Group**
Pentti Tienari	Hospital District of Helsinki and Uusimaa, Helsinki, Finland	**Clinical Groups (FinnGen phases 1&2)**	**Neurology Group**
Juha Rinne	Wellbeing Services County of Southwest Finland, Turku, Finland	**Clinical Groups (FinnGen phases 1&2)**	**Neurology Group**
Roosa Kallionpää	Wellbeing Services County of Southwest Finland, Turku, Finland	**Clinical Groups (FinnGen phases 1&2)**	**Neurology Group**
Juulia Partanen	Institute for Molecular Medicine Finland, HiLIFE, University of Helsinki, Finland	**Clinical Groups (FinnGen phases 1&2)**	**Neurology Group**
Adam Ziemann	Abbvie, Chicago, IL, United States	**Clinical Groups (FinnGen phases 1&2)**	**Neurology Group**
Nizar Smaoui	Abbvie, Chicago, IL, United States	**Clinical Groups (FinnGen phases 1&2)**	**Neurology Group**
Anne Lehtonen	Abbvie, Chicago, IL, United States	**Clinical Groups (FinnGen phases 1&2)**	**Neurology Group**
Susan Eaton	Biogen, Cambridge, MA, United States	**Clinical Groups (FinnGen phases 1&2)**	**Neurology Group**

Shameek Biswas	Bristol Myers Squibb, New York, NY, United States	Clinical Groups (FinnGen phases 1&2)	**Neurology Group**
Natalie Bowers	Genentech, San Francisco, CA, United States	Clinical Groups (FinnGen phases 1&2)	**Neurology Group**
Edmond Teng	Genentech, San Francisco, CA, United States	Clinical Groups (FinnGen phases 1&2)	**Neurology Group**
Rion Pendergrass	Genentech, San Francisco, CA, United States	Clinical Groups (FinnGen phases 1&2)	**Neurology Group**
Fanli Xu	GlaxoSmithKline, Brentford, United Kingdom	Clinical Groups (FinnGen phases 1&2)	**Neurology Group**
Laura Addis	GlaxoSmithKline, Brentford, United Kingdom	Clinical Groups (FinnGen phases 1&2)	**Neurology Group**
John Eicher	GlaxoSmithKline, Brentford, United Kingdom	Clinical Groups (FinnGen phases 1&2)	**Neurology Group**
Qingqin S Li	Johnson & Johnson Innovative Medicine, Titusville, NJ 08560, United States	Clinical Groups (FinnGen phases 1&2)	**Neurology Group**
Karen He	Johnson & Johnson Innovative Medicine, Spring House, PA, United States	Clinical Groups (FinnGen phases 1&2)	**Neurology Group**
Ekaterina Khramtsova	Johnson & Johnson Innovative Medicine, Spring House, PA, United States	Clinical Groups (FinnGen phases 1&2)	**Neurology Group**
Neha Raghavan	Merck, Kenilworth, NJ, United States	Clinical Groups (FinnGen phases 1&2)	**Neurology Group**
Martti Färkkilä	Hospital District of Helsinki and Uusimaa, Helsinki, Finland	Clinical Groups (FinnGen phases 1&2)	**Gastroenterology Group**
Jukka Koskela	Hospital District of Helsinki and Uusimaa, Helsinki, Finland	Clinical Groups (FinnGen phases 1&2)	**Gastroenterology Group**
Sampsa Pikkarainen	Hospital District of Helsinki and Uusimaa, Helsinki, Finland	Clinical Groups (FinnGen phases 1&2)	**Gastroenterology Group**
Airi Jussila	Wellbeing Services County of Pirkanmaa, Tampere, Finland	Clinical Groups (FinnGen phases 1&2)	**Gastroenterology Group**
Katri Kaukinen	Wellbeing Services County of Pirkanmaa, Tampere, Finland	Clinical Groups (FinnGen phases 1&2)	**Gastroenterology Group**
Timo Blomster	Wellbeing services county of North Ostrobothnia, Oulu, Finland	Clinical Groups (FinnGen phases 1&2)	**Gastroenterology Group**
Mikko Kiviniemi	Wellbeing services county of North Savo, Kuopio, Finland	Clinical Groups (FinnGen phases 1&2)	**Gastroenterology Group**
Markku Voutilainen	Wellbeing Services County of Southwest Finland, Turku, Finland	Clinical Groups (FinnGen phases 1&2)	**Gastroenterology Group**
Mark Daly	Institute for Molecular Medicine Finland (FIMM), HiLIFE, University of Helsinki, Helsinki, Finland; Broad Institute of MIT and Harvard; Massachusetts General Hospital, Boston, MA, United States	Clinical Groups (FinnGen phases 1&2)	**Gastroenterology Group**
Jeffrey Waring	Abbvie, Chicago, IL, United States	Clinical Groups (FinnGen phases 1&2)	**Gastroenterology Group**
Nizar Smaoui	Abbvie, Chicago, IL, United States	Clinical Groups (FinnGen phases 1&2)	**Gastroenterology Group**
Fedik Rahimov	Abbvie, Chicago, IL, United States	Clinical Groups (FinnGen phases 1&2)	**Gastroenterology Group**
Anne Lehtonen	Abbvie, Chicago, IL, United States	Clinical Groups (FinnGen phases 1&2)	**Gastroenterology Group**

Tim Lu	Genentech, San Francisco, CA, United States	Clinical Groups (FinnGen phases 1&2)	**Gastroenterology Group**
Natalie Bowers	Genentech, San Francisco, CA, United States	Clinical Groups (FinnGen phases 1&2)	**Gastroenterology Group**
Rion Pendergrass	Genentech, San Francisco, CA, United States	Clinical Groups (FinnGen phases 1&2)	**Gastroenterology Group**
Linda McCarthy	GlaxoSmithKline, Brentford, United Kingdom	Clinical Groups (FinnGen phases 1&2)	**Gastroenterology Group**
Amy Hart	Johnson & Johnson Innovative Medicine, Spring House, PA, United States	Clinical Groups (FinnGen phases 1&2)	**Gastroenterology Group**
Meijian Guan	Johnson & Johnson Innovative Medicine, Spring House, PA, United States	Clinical Groups (FinnGen phases 1&2)	**Gastroenterology Group**
Jason Miller	Merck, Kenilworth, NJ, United States	Clinical Groups (FinnGen phases 1&2)	**Gastroenterology Group**
Kirsi Kalpala	Pfizer, New York, NY, United States	Clinical Groups (FinnGen phases 1&2)	**Gastroenterology Group**
Melissa Miller	Pfizer, New York, NY, United States	Clinical Groups (FinnGen phases 1&2)	**Gastroenterology Group**
Xinli Hu	Pfizer, New York, NY, United States	Clinical Groups (FinnGen phases 1&2)	**Gastroenterology Group**
Kari Eklund	Hospital District of Helsinki and Uusimaa, Helsinki, Finland	Clinical Groups (FinnGen phases 1&2)	**Rheumatology Group**
Antti Palomäki	Wellbeing Services County of Southwest Finland, Turku, Finland	Clinical Groups (FinnGen phases 1&2)	**Rheumatology Group**
Pia Isomäki	Wellbeing Services County of Pirkanmaa, Tampere, Finland	Clinical Groups (FinnGen phases 1&2)	**Rheumatology Group**
Laura Pirilä	Wellbeing Services County of Southwest Finland, Turku, Finland	Clinical Groups (FinnGen phases 1&2)	**Rheumatology Group**
Oili Kaipiainen-Seppänen	Wellbeing services county of North Savo, Kuopio, Finland	Clinical Groups (FinnGen phases 1&2)	**Rheumatology Group**
Johanna Huhtakangas	Wellbeing services county of North Ostrobothnia, Oulu, Finland	Clinical Groups (FinnGen phases 1&2)	**Rheumatology Group**
Nina Mars	Institute for Molecular Medicine Finland (FIMM), HiLIFE, University of Helsinki, Helsinki, Finland	Clinical Groups (FinnGen phases 1&2)	**Rheumatology Group**
Jeffrey Waring	Abbvie, Chicago, IL, United States	Clinical Groups (FinnGen phases 1&2)	**Rheumatology Group**
Fedik Rahimov	Abbvie, Chicago, IL, United States	Clinical Groups (FinnGen phases 1&2)	**Rheumatology Group**
Apinya Lertratanakul	Abbvie, Chicago, IL, United States	Clinical Groups (FinnGen phases 1&2)	**Rheumatology Group**
Nizar Smaoui	Abbvie, Chicago, IL, United States	Clinical Groups (FinnGen phases 1&2)	**Rheumatology Group**
Anne Lehtonen	Abbvie, Chicago, IL, United States	Clinical Groups (FinnGen phases 1&2)	**Rheumatology Group**
Coralie Viollet	AstraZeneca, Cambridge, United Kingdom	Clinical Groups (FinnGen phases 1&2)	**Rheumatology Group**
Marla Hochfeld	Bristol Myers Squibb, New York, NY, United States	Clinical Groups (FinnGen phases 1&2)	**Rheumatology Group**

Natalie Bowers	Genentech, San Francisco, CA, United States	Clinical Groups (FinnGen phases 1&2)	**Rheumatology Group**
Rion Pendergrass	Genentech, San Francisco, CA, United States	Clinical Groups (FinnGen phases 1&2)	**Rheumatology Group**
Jorge Esparza Gordillo	GlaxoSmithKline, Brentford, United Kingdom	Clinical Groups (FinnGen phases 1&2)	**Rheumatology Group**
Dawn Waterworth	Johnson & Johnson Innovative Medicine, Spring House, PA, United States	Clinical Groups (FinnGen phases 1&2)	**Rheumatology Group**
Fabiana Farias	Merck, Kenilworth, NJ, United States	Clinical Groups (FinnGen phases 1&2)	**Rheumatology Group**
Kirsi Kalpala	Pfizer, New York, NY, United States	Clinical Groups (FinnGen phases 1&2)	**Rheumatology Group**
Nan Bing	Pfizer, New York, NY, United States	Clinical Groups (FinnGen phases 1&2)	**Rheumatology Group**
Xinli Hu	Pfizer, New York, NY, United States	Clinical Groups (FinnGen phases 1&2)	**Rheumatology Group**
Tarja Laitinen	Wellbeing Services County of Pirkanmaa, Tampere, Finland	Clinical Groups (FinnGen phases 1&2)	**Pulmonology Group**
Margit Pelkonen	Wellbeing services county of North Savo, Kuopio, Finland	Clinical Groups (FinnGen phases 1&2)	**Pulmonology Group**
Paula Kauppi	Hospital District of Helsinki and Uusimaa, Helsinki, Finland	Clinical Groups (FinnGen phases 1&2)	**Pulmonology Group**
Hannu Kankaanranta	University of Gothenburg, Gothenburg, Sweden/ Seinäjoki Central Hospital, Seinäjoki, Finland/ Tampere University, Tampere, Finland	Clinical Groups (FinnGen phases 1&2)	**Pulmonology Group**
Terttu Harju	Wellbeing services county of North Ostrobothnia, Oulu, Finland	Clinical Groups (FinnGen phases 1&2)	**Pulmonology Group**
Riitta Lahesmaa	Wellbeing Services County of Southwest Finland, Turku, Finland	Clinical Groups (FinnGen phases 1&2)	**Pulmonology Group**
Nizar Smaoui	Abbvie, Chicago, IL, United States	Clinical Groups (FinnGen phases 1&2)	**Pulmonology Group**
Coralie Viollet	AstraZeneca, Cambridge, United Kingdom	Clinical Groups (FinnGen phases 1&2)	**Pulmonology Group**
Susan Eaton	Biogen, Cambridge, MA, United States	Clinical Groups (FinnGen phases 1&2)	**Pulmonology Group**
Hubert Chen	Genentech, San Francisco, CA, United States	Clinical Groups (FinnGen phases 1&2)	**Pulmonology Group**
Rion Pendergrass	Genentech, San Francisco, CA, United States	Clinical Groups (FinnGen phases 1&2)	**Pulmonology Group**
Natalie Bowers	Genentech, San Francisco, CA, United States	Clinical Groups (FinnGen phases 1&2)	**Pulmonology Group**
Joanna Betts	GlaxoSmithKline, Brentford, United Kingdom	Clinical Groups (FinnGen phases 1&2)	**Pulmonology Group**
Kirsi Auro	GlaxoSmithKline, Espoo, Finland	Clinical Groups (FinnGen phases 1&2)	**Pulmonology Group**
Rajashree Mishra	GlaxoSmithKline, Brentford, United Kingdom	Clinical Groups (FinnGen phases 1&2)	**Pulmonology Group**
Majd Mouded	Novartis, Basel, Switzerland	Clinical Groups (FinnGen phases 1&2)	**Pulmonology Group**

Debby Ngo	Novartis, Basel, Switzerland	Clinical Groups (FinnGen phases 1&2)	**Pulmonology Group**
Teemu Niiranen	University of Turku, Turku, Finland; Finnish Institute for Health and Welfare (THL), Helsinki, Finland	Clinical Groups (FinnGen phases 1&2)	**Cardiometabolic Diseases Group**
Felix Vaura	Finnish Institute for Health and Welfare (THL), Helsinki, Finland	Clinical Groups (FinnGen phases 1&2)	**Cardiometabolic Diseases Group**
Veikko Salomaa	Finnish Institute for Health and Welfare (THL), Helsinki, Finland	Clinical Groups (FinnGen phases 1&2)	**Cardiometabolic Diseases Group**
Kaj Metsärinne	Wellbeing Services County of Southwest Finland, Turku, Finland	Clinical Groups (FinnGen phases 1&2)	**Cardiometabolic Diseases Group**
Jenni Aittokallio	Wellbeing Services County of Southwest Finland, Turku, Finland	Clinical Groups (FinnGen phases 1&2)	**Cardiometabolic Diseases Group**
Mika Kähönen	Finnish Clinical Biobank Tampere / University of Tampere / Wellbeing Services County of Pirkanmaa, Tampere, Finland	Clinical Groups (FinnGen phases 1&2)	**Cardiometabolic Diseases Group**
Jussi Hernesniemi	Wellbeing Services County of Pirkanmaa, Tampere, Finland	Clinical Groups (FinnGen phases 1&2)	**Cardiometabolic Diseases Group**
Daniel Gordin	Hospital District of Helsinki and Uusimaa, Helsinki, Finland	Clinical Groups (FinnGen phases 1&2)	**Cardiometabolic Diseases Group**
Juha Sinisalo	Hospital District of Helsinki and Uusimaa, Helsinki, Finland	Clinical Groups (FinnGen phases 1&2)	**Cardiometabolic Diseases Group**
Marja-Riitta Taskinen	Hospital District of Helsinki and Uusimaa, Helsinki, Finland	Clinical Groups (FinnGen phases 1&2)	**Cardiometabolic Diseases Group**
Tiinamaija Tuomi	Institute for Molecular Medicine Finland (FIMM), HiLIFE, University of Helsinki, Helsinki, Finland; Hospital District of Helsinki and Uusimaa, Helsinki, Finland	Clinical Groups (FinnGen phases 1&2)	**Cardiometabolic Diseases Group**
Timo Hiltunen	Hospital District of Helsinki and Uusimaa, Helsinki, Finland	Clinical Groups (FinnGen phases 1&2)	**Cardiometabolic Diseases Group**
Jari Laukkanen	Central Finland Biobank / University of Jyväskylä / Wellbeing Services County of Central Finland, Jyväskylä, Finland	Clinical Groups (FinnGen phases 1&2)	**Cardiometabolic Diseases Group**
Amanda Elliott	Institute for Molecular Medicine Finland (FIMM), HiLIFE, University of Helsinki, Helsinki, Finland; Broad Institute, Cambridge, MA, USA and Massachusetts General Hospital, Boston, MA, USA	Clinical Groups (FinnGen phases 1&2)	**Cardiometabolic Diseases Group**
Mary Pat Reeve	Institute for Molecular Medicine Finland (FIMM), HiLIFE, University of Helsinki, Helsinki, Finland	Clinical Groups (FinnGen phases 1&2)	**Cardiometabolic Diseases Group**
Sanni Ruotsalainen	Institute for Molecular Medicine Finland (FIMM), HiLIFE, University of Helsinki, Helsinki, Finland	Clinical Groups (FinnGen phases 1&2)	**Cardiometabolic Diseases Group**
Dirk Paul	Astra Zeneca, Cambridge, United Kingdom	Clinical Groups (FinnGen phases 1&2)	**Cardiometabolic Diseases Group**
Natalie Bowers	Genentech, San Francisco, CA, United States	Clinical Groups (FinnGen phases 1&2)	**Cardiometabolic Diseases Group**
Rion Pendergrass	Genentech, San Francisco, CA, United States	Clinical Groups (FinnGen phases 1&2)	**Cardiometabolic Diseases Group**
Audrey Chu	GlaxoSmithKline, Brentford, United Kingdom	Clinical Groups (FinnGen phases 1&2)	**Cardiometabolic Diseases Group**
Dermot Reilly	Johnson & Johnson Innovative Medicine, Boston, MA, United States	Clinical Groups (FinnGen phases 1&2)	**Cardiometabolic Diseases Group**
Mike Mendelson	Novartis, Boston, MA, United States	Clinical Groups (FinnGen phases 1&2)	**Cardiometabolic Diseases Group**
Jaakko Parkkinen	Pfizer, New York, NY, United States	Clinical Groups (FinnGen phases 1&2)	**Cardiometabolic Diseases Group**

Melissa Miller	Pfizer, New York, NY, United States	Clinical Groups (FinnGen phases 1&2)	**Cardiometabolic Diseases Group**
Tuomo Meretoja	Helsinki University Hospital and University of Helsinki, Helsinki, Finland	Clinical Groups (FinnGen phases 1&2)	**Oncology Group**
Heikki Joensuu	Helsinki University Hospital and University of Helsinki, Helsinki, Finland	Clinical Groups (FinnGen phases 1&2)	**Oncology Group**
Olli Carpén	Hospital District of Helsinki and Uusimaa, Helsinki, Finland	Clinical Groups (FinnGen phases 1&2)	**Oncology Group**
Johanna Mattson	Hospital District of Helsinki and Uusimaa, Helsinki, Finland	Clinical Groups (FinnGen phases 1&2)	**Oncology Group**
Eveliina Salminen	Hospital District of Helsinki and Uusimaa, Helsinki, Finland	Clinical Groups (FinnGen phases 1&2)	**Oncology Group**
Annika Auranen	Wellbeing Services County of Pirkanmaa, Tampere, Finland	Clinical Groups (FinnGen phases 1&2)	**Oncology Group**
Peeter Karihtala	Helsinki University Hospital and University of Helsinki, Helsinki, Finland	Clinical Groups (FinnGen phases 1&2)	**Oncology Group**
Päivi Auvinen	Wellbeing services county of North Savo, Kuopio, Finland	Clinical Groups (FinnGen phases 1&2)	**Oncology Group**
Klaus Elenius	Wellbeing Services County of Southwest Finland, Turku, Finland	Clinical Groups (FinnGen phases 1&2)	**Oncology Group**
Johanna Schleutker	Wellbeing Services County of Southwest Finland, Turku, Finland	Clinical Groups (FinnGen phases 1&2)	**Oncology Group**
Esa Pitkänen	Institute for Molecular Medicine Finland (FIMM), HiLIFE, University of Helsinki, Helsinki, Finland	Clinical Groups (FinnGen phases 1&2)	**Oncology Group**
Nina Mars	Institute for Molecular Medicine Finland (FIMM), HiLIFE, University of Helsinki, Helsinki, Finland	Clinical Groups (FinnGen phases 1&2)	**Oncology Group**
Mark Daly	Institute for Molecular Medicine Finland (FIMM), HiLIFE, University of Helsinki, Helsinki, Finland; Broad Institute of MIT and Harvard; Massachusetts General Hospital, Boston, MA, United States	Clinical Groups (FinnGen phases 1&2)	**Oncology Group**
Relja Popovic	Abbvie, Chicago, IL, United States	Clinical Groups (FinnGen phases 1&2)	**Oncology Group**
Jeffrey Waring	Abbvie, Chicago, IL, United States	Clinical Groups (FinnGen phases 1&2)	**Oncology Group**
Bridget Riley-Gillis	Abbvie, Chicago, IL, United States	Clinical Groups (FinnGen phases 1&2)	**Oncology Group**
Anne Lehtonen	Abbvie, Chicago, IL, United States	Clinical Groups (FinnGen phases 1&2)	**Oncology Group**
Margarete Fabre	AstraZeneca, Cambridge, United Kingdom	Clinical Groups (FinnGen phases 1&2)	**Oncology Group**
Jennifer Schutzman	Genentech, San Francisco, CA, United States	Clinical Groups (FinnGen phases 1&2)	**Oncology Group**
Natalie Bowers	Genentech, San Francisco, CA, United States	Clinical Groups (FinnGen phases 1&2)	**Oncology Group**
Rion Pendergrass	Genentech, San Francisco, CA, United States	Clinical Groups (FinnGen phases 1&2)	**Oncology Group**
Diptee Kulkarni	GlaxoSmithKline, Brentford, United Kingdom	Clinical Groups (FinnGen phases 1&2)	**Oncology Group**
Alessandro Porello	Johnson & Johnson Innovative Medicine, Spring House, PA, United States	Clinical Groups (FinnGen phases 1&2)	**Oncology Group**

Andrey Loboda	Merck, Kenilworth, NJ, United States	Clinical Groups (FinnGen phases 1&2)	**Oncology Group**
Stefan McDonough	Pfizer, New York, NY, United States	Clinical Groups (FinnGen phases 1&2)	**Oncology Group**
Kai Kaarniranta	Wellbeing services county of North Savo, Kuopio, Finland; University of Lodz, Lodz, Poland	Clinical Groups (FinnGen phases 1&2)	**Opthalmology Group**
Joni A Turunen	Helsinki University Hospital and University of Helsinki, Helsinki, Finland; Folkhälsan Research Center, Helsinki, Finland	Clinical Groups (FinnGen phases 1&2)	**Opthalmology Group**
Terhi Ollila	Hospital District of Helsinki and Uusimaa, Helsinki, Finland	Clinical Groups (FinnGen phases 1&2)	**Opthalmology Group**
Hannu Uusitalo	Wellbeing Services County of Pirkanmaa, Tampere, Finland	Clinical Groups (FinnGen phases 1&2)	**Opthalmology Group**
Juha Karjalainen	Institute for Molecular Medicine Finland (FIMM), HiLIFE, University of Helsinki, Helsinki, Finland	Clinical Groups (FinnGen phases 1&2)	**Opthalmology Group**
Esa Pitkänen	Institute for Molecular Medicine Finland (FIMM), HiLIFE, University of Helsinki, Helsinki, Finland	Clinical Groups (FinnGen phases 1&2)	**Opthalmology Group**
Mengzhen Liu	Abbvie, Chicago, IL, United States	Clinical Groups (FinnGen phases 1&2)	**Opthalmology Group**
Erich Strauss	Genentech, San Francisco, CA, United States	Clinical Groups (FinnGen phases 1&2)	**Opthalmology Group**
Natalie Bowers	Genentech, San Francisco, CA, United States	Clinical Groups (FinnGen phases 1&2)	**Opthalmology Group**
Hao Chen	Genentech, San Francisco, CA, United States	Clinical Groups (FinnGen phases 1&2)	**Opthalmology Group**
Rion Pendergrass	Genentech, San Francisco, CA, United States	Clinical Groups (FinnGen phases 1&2)	**Opthalmology Group**
Kaisa Tasanen	Wellbeing services county of North Ostrobothnia, Oulu, Finland	Clinical Groups (FinnGen phases 1&2)	**Dermatology Group**
Laura Huilaja	Wellbeing services county of North Ostrobothnia, Oulu, Finland	Clinical Groups (FinnGen phases 1&2)	**Dermatology Group**
Katariina Hannula-Jouppi	Hospital District of Helsinki and Uusimaa, Helsinki, Finland	Clinical Groups (FinnGen phases 1&2)	**Dermatology Group**
Teea Salmi	Wellbeing Services County of Pirkanmaa, Tampere, Finland	Clinical Groups (FinnGen phases 1&2)	**Dermatology Group**
Sirkku Peltonen	Wellbeing Services County of Southwest Finland, Turku, Finland	Clinical Groups (FinnGen phases 1&2)	**Dermatology Group**
Leena Koulu	Wellbeing Services County of Southwest Finland, Turku, Finland	Clinical Groups (FinnGen phases 1&2)	**Dermatology Group**
Nizar Smaoui	Abbvie, Chicago, IL, United States	Clinical Groups (FinnGen phases 1&2)	**Dermatology Group**
Fedik Rahimov	Abbvie, Chicago, IL, United States	Clinical Groups (FinnGen phases 1&2)	**Dermatology Group**
Anne Lehtonen	Abbvie, Chicago, IL, United States	Clinical Groups (FinnGen phases 1&2)	**Dermatology Group**
David Choy	Genentech, San Francisco, CA, United States	Clinical Groups (FinnGen phases 1&2)	**Dermatology Group**
Rion Pendergrass	Genentech, San Francisco, CA, United States	Clinical Groups (FinnGen phases 1&2)	**Dermatology Group**

Dawn Waterworth	Johnson & Johnson Innovative Medicine, Spring House, PA, United States	Clinical Groups (FinnGen phases 1&2)	**Dermatology Group**
Kirsi Kalpala	Pfizer, New York, NY, United States	Clinical Groups (FinnGen phases 1&2)	**Dermatology Group**
Ying Wu	Pfizer, New York, NY, United States	Clinical Groups (FinnGen phases 1&2)	**Dermatology Group**
Pirkko Pussinen	Hospital District of Helsinki and Uusimaa, Helsinki, Finland	Clinical Groups (FinnGen phases 1&2)	**Odontology Group**
Aino Salminen	Hospital District of Helsinki and Uusimaa, Helsinki, Finland	Clinical Groups (FinnGen phases 1&2)	**Odontology Group**
Tuula Salo	Hospital District of Helsinki and Uusimaa, Helsinki, Finland	Clinical Groups (FinnGen phases 1&2)	**Odontology Group**
David Rice	Hospital District of Helsinki and Uusimaa, Helsinki, Finland	Clinical Groups (FinnGen phases 1&2)	**Odontology Group**
Pekka Nieminen	Hospital District of Helsinki and Uusimaa, Helsinki, Finland	Clinical Groups (FinnGen phases 1&2)	**Odontology Group**
Ulla Palotie	Hospital District of Helsinki and Uusimaa, Helsinki, Finland	Clinical Groups (FinnGen phases 1&2)	**Odontology Group**
Maria Siponen	Wellbeing services county of North Savo, Kuopio, Finland	Clinical Groups (FinnGen phases 1&2)	**Odontology Group**
Liisa Suominen	Wellbeing services county of North Savo, Kuopio, Finland	Clinical Groups (FinnGen phases 1&2)	**Odontology Group**
Päivi Mäntylä	Wellbeing services county of North Savo, Kuopio, Finland	Clinical Groups (FinnGen phases 1&2)	**Odontology Group**
Ulvi Gursoy	Wellbeing Services County of Southwest Finland, Turku, Finland	Clinical Groups (FinnGen phases 1&2)	**Odontology Group**
Vuokko Anttonen	Wellbeing services county of North Ostrobothnia, Oulu, Finland	Clinical Groups (FinnGen phases 1&2)	**Odontology Group**
Kirsi Sipilä	Oulu University Hospital and University of Oulu, Oulu, Finland	Clinical Groups (FinnGen phases 1&2)	**Odontology Group**
Rion Pendergrass	Genentech, San Francisco, CA, United States	Clinical Groups (FinnGen phases 1&2)	**Odontology Group**
Hannele Laivuori	Institute for Molecular Medicine Finland (FIMM), HiLIFE, University of Helsinki, Helsinki, Finland	Clinical Groups (FinnGen phases 1&2)	**Women's Health and Reproduction Group**
Venla Kurra	Wellbeing Services County of Pirkanmaa, Tampere, Finland	Clinical Groups (FinnGen phases 1&2)	**Women's Health and Reproduction Group**
Laura Kotaniemi-Talonen	Wellbeing Services County of Pirkanmaa, Tampere, Finland	Clinical Groups (FinnGen phases 1&2)	**Women's Health and Reproduction Group**
Oskari Heikinheimo	Hospital District of Helsinki and Uusimaa, Helsinki, Finland	Clinical Groups (FinnGen phases 1&2)	**Women's Health and Reproduction Group**
Ilkka Kalliala	Hospital District of Helsinki and Uusimaa, Helsinki, Finland	Clinical Groups (FinnGen phases 1&2)	**Women's Health and Reproduction Group**
Lauri Aaltonen	Hospital District of Helsinki and Uusimaa, Helsinki, Finland	Clinical Groups (FinnGen phases 1&2)	**Women's Health and Reproduction Group**
Varpu Jokimaa	Wellbeing Services County of Southwest Finland, Turku, Finland	Clinical Groups (FinnGen phases 1&2)	**Women's Health and Reproduction Group**
Johannes Kettunen	Northern Finland Biobank Borealis / University of Oulu / Wellbeing services county of North Ostrobothnia, Oulu, Finland	Clinical Groups (FinnGen phases 1&2)	**Women's Health and Reproduction Group**

Marja Vääräsmäki	Wellbeing services county of North Ostrobothnia, Oulu, Finland	Clinical Groups (FinnGen phases 1&2)	Women's Health and Reproduction Group
Outi Uimari	Wellbeing services county of North Ostrobothnia, Oulu, Finland	Clinical Groups (FinnGen phases 1&2)	Women's Health and Reproduction Group
Laure Morin-Papunen	Wellbeing services county of North Ostrobothnia, Oulu, Finland	Clinical Groups (FinnGen phases 1&2)	Women's Health and Reproduction Group
Maarit Niinimäki	Wellbeing services county of North Ostrobothnia, Oulu, Finland	Clinical Groups (FinnGen phases 1&2)	Women's Health and Reproduction Group
Terhi Piltonen	Wellbeing services county of North Ostrobothnia, Oulu, Finland	Clinical Groups (FinnGen phases 1&2)	Women's Health and Reproduction Group
Katja Kivinen	Institute for Molecular Medicine Finland (FIMM), HiLIFE, University of Helsinki, Helsinki, Finland	Clinical Groups (FinnGen phases 1&2)	Women's Health and Reproduction Group
Elisabeth Widen	Institute for Molecular Medicine Finland (FIMM), HiLIFE, University of Helsinki, Helsinki, Finland	Clinical Groups (FinnGen phases 1&2)	Women's Health and Reproduction Group
Taru Tukiainen	Institute for Molecular Medicine Finland (FIMM), HiLIFE, University of Helsinki, Helsinki, Finland	Clinical Groups (FinnGen phases 1&2)	Women's Health and Reproduction Group
Mary Pat Reeve	Institute for Molecular Medicine Finland (FIMM), HiLIFE, University of Helsinki, Helsinki, Finland	Clinical Groups (FinnGen phases 1&2)	Women's Health and Reproduction Group
Mark Daly	Institute for Molecular Medicine Finland (FIMM), HiLIFE, University of Helsinki, Helsinki, Finland; Broad Institute of MIT and Harvard; Massachusetts General Hospital, Boston, MA, United States	Clinical Groups (FinnGen phases 1&2)	Women's Health and Reproduction Group
Niko Välimäki	University of Helsinki, Helsinki, Finland	Clinical Groups (FinnGen phases 1&2)	Women's Health and Reproduction Group
Eija Laakkonen	University of Jyväskylä, Jyväskylä, Finland	Clinical Groups (FinnGen phases 1&2)	Women's Health and Reproduction Group
Jaakko Tyrmi	University of Oulu, Oulu, Finland / University of Tampere, Tampere, Finland	Clinical Groups (FinnGen phases 1&2)	Women's Health and Reproduction Group
Heidi Silven	University of Oulu, Oulu, Finland	Clinical Groups (FinnGen phases 1&2)	Women's Health and Reproduction Group
Eeva Sliz	University of Oulu, Oulu, Finland	Clinical Groups (FinnGen phases 1&2)	Women's Health and Reproduction Group
Riikka Arffman	University of Oulu, Oulu, Finland	Clinical Groups (FinnGen phases 1&2)	Women's Health and Reproduction Group
Susanna Savukoski	University of Oulu, Oulu, Finland	Clinical Groups (FinnGen phases 1&2)	Women's Health and Reproduction Group
Triin Laisk	Estonian biobank, Tartu, Estonia	Clinical Groups (FinnGen phases 1&2)	Women's Health and Reproduction Group
Natalia Pujol	Estonian biobank, Tartu, Estonia	Clinical Groups (FinnGen phases 1&2)	Women's Health and Reproduction Group
Mengzhen Liu	AbbVie, Chicago, IL, United States	Clinical Groups (FinnGen phases 1&2)	Women's Health and Reproduction Group
Bridget Riley-Gillis	AbbVie, Chicago, IL, United States	Clinical Groups (FinnGen phases 1&2)	Women's Health and Reproduction Group
Rion Pendergrass	Genentech, San Francisco, CA, United States	Clinical Groups (FinnGen phases 1&2)	Women's Health and Reproduction Group
Janet Kumar	GlaxoSmithKline, Collegeville, PA, United States	Clinical Groups (FinnGen phases 1&2)	Women's Health and Reproduction Group
Iiris Hovatta	University of Helsinki, Finland	Clinical Groups (FinnGen phases 1&2)	Depression group

Erkki Isometsä	Hospital District of Helsinki and Uusimaa, Helsinki, Finland	Clinical Groups (FinnGen phases 1&2)	**Depression group**
Hanna Ollila	Institute for Molecular Medicine Finland (FIMM), HiLIFE, University of Helsinki, Helsinki, Finland	Clinical Groups (FinnGen phases 1&2)	**Depression group**
Jaana Suvisaari	Finnish Institute for Health and Welfare (THL), Helsinki, Finland	Clinical Groups (FinnGen phases 1&2)	**Depression group**
Antti Mäkitie	University of Helsinki and Helsinki University Hospital, Helsinki, Finland	Clinical Groups (FinnGen phases 1&2)	**ENT (ear, nose and throath) Group**
Argyro Bizaki-Vallaskangas	Wellbeing Services County of Pirkanmaa, Tampere, Finland	Clinical Groups (FinnGen phases 1&2)	**ENT (ear, nose and throath) Group**
Sanna Toppila-Salmi	University of Eastern Finland and Kuopio University Hospital, Kuopio, Finland; Helsinki University Hospital and University of Helsinki, Finland	Clinical Groups (FinnGen phases 1&2)	**ENT (ear, nose and throath) Group**
Tytti Willberg	Wellbeing Services County of Southwest Finland, Turku, Finland	Clinical Groups (FinnGen phases 1&2)	**ENT (ear, nose and throath) Group**
Elmo Saarentaus	Institute for Molecular Medicine Finland (FIMM), HiLIFE, University of Helsinki, Helsinki, Finland	Clinical Groups (FinnGen phases 1&2)	**ENT (ear, nose and throath) Group**
Antti Aarnisalo	Hospital District of Helsinki and Uusimaa, Helsinki, Finland	Clinical Groups (FinnGen phases 1&2)	**ENT (ear, nose and throath) Group**
Eveliina Salminen	Hospital District of Helsinki and Uusimaa, Helsinki, Finland	Clinical Groups (FinnGen phases 1&2)	**ENT (ear, nose and throath) Group**
Elisa Rahikkala	Northern Ostrobothnia Hospital District, Oulu, Finland	Clinical Groups (FinnGen phases 1&2)	**ENT (ear, nose and throath) Group**
Johannes Kettunen	Northern Finland Biobank Borealis / University of Oulu / Wellbeing services county of North Ostrobothnia, Oulu, Finland	Clinical Groups (FinnGen phases 1&2)	**ENT (ear, nose and throath) Group**
Kristiina Aittomäki	Helsinki University Central Hospital, Helsinki, Finland	Clinical Groups (FinnGen phases 1&2)	**POI (premature ovarian failure) Group**
Fredrik Åberg	Helsinki University Hospital and University of Helsinki, Helsinki, Finland	Clinical Groups (FinnGen phases 1&2)	**LiverScore Group**
Joel Rämö	Institute for Molecular Medicine Finland (FIMM), HiLIFE, University of Helsinki, Helsinki, Finland; Broad Institute, Cambridge, MA, United States	Clinical Task Forces (FinnGen phase 3)	**Eye diseases Task Force**
Mark Daly	Institute for Molecular Medicine Finland (FIMM), HiLIFE, University of Helsinki, Helsinki, Finland; Broad Institute of MIT and Harvard; Massachusetts General Hospital, Boston, MA, United States	Clinical Task Forces (FinnGen phase 3)	**Eye diseases Task Force**
Mary Pat Reeve	Institute for Molecular Medicine Finland (FIMM), HiLIFE, University of Helsinki, Helsinki, Finland; Broad Institute, Cambridge, MA, United States	Clinical Task Forces (FinnGen phase 3)	**Eye diseases Task Force**
Muhammad Adnan Khan	Institute for Molecular Medicine Finland (FIMM), HiLIFE, University of Helsinki, Helsinki, Finland	Clinical Task Forces (FinnGen phase 3)	**Eye diseases Task Force**
Johanna Mäkelä	Finnish Biobank Cooperative - FINBB	Clinical Task Forces (FinnGen phase 3)	**Eye diseases Task Force**
Ilkka Immonen	Hospital District of Helsinki and Uusimaa, Helsinki, Finland	Clinical Task Forces (FinnGen phase 3)	**Eye diseases Task Force**
Kai Kaarniranta	Wellbeing services county of North Savo, Kuopio, Finland; University of Lodz, Lodz, Poland	Clinical Task Forces (FinnGen phase 3)	**Eye diseases Task Force**
Joni A Turunen	Helsinki University Hospital and University of Helsinki, Helsinki, Finland; Folkhälsan Research Center, Helsinki, Finland	Clinical Task Forces (FinnGen phase 3)	**Eye diseases Task Force**
Anneke Den Hollander	AbbVie, Chicago, IL, United States	Clinical Task Forces (FinnGen phase 3)	**Eye diseases Task Force**
Bridget Riley-Gillis	AbbVie, Chicago, IL, United States	Clinical Task Forces (FinnGen phase 3)	**Eye diseases Task Force**

Mengzhen Liu	AbbVie, Chicago, IL, United States	Clinical Task Forces (FinnGen phase 3)	**Eye diseases Task Force**
Nizar Smaoui	AbbVie, Chicago, IL, United States	Clinical Task Forces (FinnGen phase 3)	**Eye diseases Task Force**
Fabio Baschiera	Bayer AG, Leverkusen, Germany	Clinical Task Forces (FinnGen phase 3)	**Eye diseases Task Force**
Hans van Leeuwen	Bayer AG, Leverkusen, Germany	Clinical Task Forces (FinnGen phase 3)	**Eye diseases Task Force**
Elke Markert	Boehringer Ingelheim, Ingelheim am Rhein, Germany	Clinical Task Forces (FinnGen phase 3)	**Eye diseases Task Force**
Brian Yaspan	Genentech, San Francisco, CA, United States	Clinical Task Forces (FinnGen phase 3)	**Eye diseases Task Force**
Charli Harlow	GlaxoSmithKline, Collegeville, PA, United States	Clinical Task Forces (FinnGen phase 3)	**Eye diseases Task Force**
Lea Sarow-Blat	GlaxoSmithKline, Collegeville, PA, United States	Clinical Task Forces (FinnGen phase 3)	**Eye diseases Task Force**
Dermont Reilly	Johnson & Johnson Innovative Medicine, Spring House, PA, United States	Clinical Task Forces (FinnGen phase 3)	**Eye diseases Task Force**
P. Dunnmon	Johnson & Johnson Innovative Medicine, Spring House, PA, United States	Clinical Task Forces (FinnGen phase 3)	**Eye diseases Task Force**
Sara Gale	Johnson & Johnson Innovative Medicine, Spring House, PA, United States	Clinical Task Forces (FinnGen phase 3)	**Eye diseases Task Force**
Fabiana Farias	Merck, Kenilworth, NJ, United States	Clinical Task Forces (FinnGen phase 3)	**Eye diseases Task Force**
Jorge Del-aguila	Merck, Kenilworth, NJ, United States	Clinical Task Forces (FinnGen phase 3)	**Eye diseases Task Force**
Catherine O'Riordan	Translational Sciences, Sanofi R&D, Framingham, MA, USA	Clinical Task Forces (FinnGen phase 3)	**Eye diseases Task Force**
Samuel Lessard	Translational Sciences, Sanofi R&D, Framingham, MA, USA	Clinical Task Forces (FinnGen phase 3)	**Eye diseases Task Force**
Suzanne Jacobs	Translational Sciences, Sanofi R&D, Framingham, MA, USA	Clinical Task Forces (FinnGen phase 3)	**Eye diseases Task Force**
Satu Koskela	Finnish Red Cross Blood Service / Finnish Hematology Registry and Clinical Biobank, Helsinki, Finland	Clinical Task Forces (FinnGen phase 3)	**Immune mediated diseases Task Force**
Anne Kerola	Institute for Molecular Medicine Finland (FIMM), HiLIFE, University of Helsinki, Helsinki, Finland	Clinical Task Forces (FinnGen phase 3)	**Immune mediated diseases Task Force**
Elisa Lahtela	Institute for Molecular Medicine Finland (FIMM), HiLIFE, University of Helsinki, Helsinki, Finland	Clinical Task Forces (FinnGen phase 3)	**Immune mediated diseases Task Force**
Helen Cooper	Institute for Molecular Medicine Finland (FIMM), HiLIFE, University of Helsinki, Helsinki, Finland	Clinical Task Forces (FinnGen phase 3)	**Immune mediated diseases Task Force**
Johanna Paltta	Institute for Molecular Medicine Finland (FIMM), HiLIFE, University of Helsinki, Helsinki, Finland; University of Turku, Turku, Finland	Clinical Task Forces (FinnGen phase 3)	**Immune mediated diseases Task Force**
Jukka Koskela	Institute for Molecular Medicine Finland (FIMM), HiLIFE, University of Helsinki, Helsinki, Finland	Clinical Task Forces (FinnGen phase 3)	**Immune mediated diseases Task Force**
Mark Daly	Institute for Molecular Medicine Finland (FIMM), HiLIFE, University of Helsinki, Helsinki, Finland; Broad Institute of MIT and Harvard; Massachusetts General Hospital, Boston, MA, United States	Clinical Task Forces (FinnGen phase 3)	**Immune mediated diseases Task Force**
Mary Pat Reeve	Institute for Molecular Medicine Finland (FIMM), HiLIFE, University of Helsinki, Helsinki, Finland; Broad Institute, Cambridge, MA, United States	Clinical Task Forces (FinnGen phase 3)	**Immune mediated diseases Task Force**

Vincent Llorens	Institute for Molecular Medicine Finland (FIMM), HiLIFE, University of Helsinki, Helsinki, Finland	**Clinical Task Forces (FinnGen phase 3)**	**Immune mediated diseases Task Force**
Martti Färkkilä	Hospital District of Helsinki and Uusimaa, Helsinki, Finland	**Clinical Task Forces (FinnGen phase 3)**	**Immune mediated diseases Task Force**
Johannes Kettunen	Northern Finland Biobank Borealis / University of Oulu / Wellbeing services county of North Ostrobothnia, Oulu, Finland	**Clinical Task Forces (FinnGen phase 3)**	**Immune mediated diseases Task Force**
Kaisa Tasanen-Maatta	Wellbeing services county of North Ostrobothnia, Oulu, Finland	**Clinical Task Forces (FinnGen phase 3)**	**Immune mediated diseases Task Force**
Laura Huilaja	Wellbeing services county of North Ostrobothnia, Oulu, Finland	**Clinical Task Forces (FinnGen phase 3)**	**Immune mediated diseases Task Force**
Minna Ruddock	Arctic biobank / University of Oulu, Oulu, Finland	**Clinical Task Forces (FinnGen phase 3)**	**Immune mediated diseases Task Force**
Aki Havulinna	Finnish Institute for Health and Welfare (THL), Helsinki, Finland	**Clinical Task Forces (FinnGen phase 3)**	**Immune mediated diseases Task Force**
Antti Palomäki	University of Turku, Turku, Finland	**Clinical Task Forces (FinnGen phase 3)**	**Immune mediated diseases Task Force**
Laura Kuusalo	University of Turku, Turku, Finland	**Clinical Task Forces (FinnGen phase 3)**	**Immune mediated diseases Task Force**
Laura Pirilä	University of Turku, Turku, Finland	**Clinical Task Forces (FinnGen phase 3)**	**Immune mediated diseases Task Force**
Fedik Rahimov	AbbVie, Chicago, IL, United States	**Clinical Task Forces (FinnGen phase 3)**	**Immune mediated diseases Task Force**
Jan Freudenberg	AbbVie, Chicago, IL, United States	**Clinical Task Forces (FinnGen phase 3)**	**Immune mediated diseases Task Force**
Nizar Smaoui	AbbVie, Chicago, IL, United States	**Clinical Task Forces (FinnGen phase 3)**	**Immune mediated diseases Task Force**
Bram Prins	Astra Zeneca, Cambridge, United Kingdom	**Clinical Task Forces (FinnGen phase 3)**	**Immune mediated diseases Task Force**
Coralie Viollet	Astra Zeneca, Cambridge, United Kingdom	**Clinical Task Forces (FinnGen phase 3)**	**Immune mediated diseases Task Force**
Eleanor Wheeler	Astra Zeneca, Cambridge, United Kingdom	**Clinical Task Forces (FinnGen phase 3)**	**Immune mediated diseases Task Force**
Kousik Kundu	Astra Zeneca, Cambridge, United Kingdom	**Clinical Task Forces (FinnGen phase 3)**	**Immune mediated diseases Task Force**
Santosh Atanur	Astra Zeneca, Cambridge, United Kingdom	**Clinical Task Forces (FinnGen phase 3)**	**Immune mediated diseases Task Force**
Hans van Leeuwen	Bayer AG, Leverkusen, Germany	**Clinical Task Forces (FinnGen phase 3)**	**Immune mediated diseases Task Force**
Himanshu Manchanda	Bayer AG, Leverkusen, Germany	**Clinical Task Forces (FinnGen phase 3)**	**Immune mediated diseases Task Force**
Karl Heilbron	Bayer AG, Leverkusen, Germany	**Clinical Task Forces (FinnGen phase 3)**	**Immune mediated diseases Task Force**
Martin Rao	Bayer AG, Leverkusen, Germany	**Clinical Task Forces (FinnGen phase 3)**	**Immune mediated diseases Task Force**
Nicole Schmidt	Bayer AG, Leverkusen, Germany	**Clinical Task Forces (FinnGen phase 3)**	**Immune mediated diseases Task Force**
Samu Kurki	Bayer AG, Leverkusen, Germany	**Clinical Task Forces (FinnGen phase 3)**	**Immune mediated diseases Task Force**

Ellen Tsai	Biogen, Cambridge, MA, United States	Clinical Task Forces (FinnGen phase 3)	**Immune mediated diseases Task Force**
Ketian Yu	Biogen, Cambridge, MA, United States	Clinical Task Forces (FinnGen phase 3)	**Immune mediated diseases Task Force**
Stephanie Loomis	Biogen, Cambridge, MA, United States	Clinical Task Forces (FinnGen phase 3)	**Immune mediated diseases Task Force**
Benjamin Sun	Bristol Myers Squibb, New York, NY, United States	Clinical Task Forces (FinnGen phase 3)	**Immune mediated diseases Task Force**
Cara Carty	Bristol Myers Squibb, New York, NY, United States	Clinical Task Forces (FinnGen phase 3)	**Immune mediated diseases Task Force**
Emily Holzinger	Bristol Myers Squibb, New York, NY, United States	Clinical Task Forces (FinnGen phase 3)	**Immune mediated diseases Task Force**
Michael Turchin	Bristol Myers Squibb, New York, NY, United States	Clinical Task Forces (FinnGen phase 3)	**Immune mediated diseases Task Force**
Neelakshi Jog	Bristol Myers Squibb, New York, NY, United States	Clinical Task Forces (FinnGen phase 3)	**Immune mediated diseases Task Force**
Frank Li	Boehringer Ingelheim, Ingelheim am Rhein, Germany	Clinical Task Forces (FinnGen phase 3)	**Immune mediated diseases Task Force**
Zhihao Ding	Boehringer Ingelheim, Ingelheim am Rhein, Germany	Clinical Task Forces (FinnGen phase 3)	**Immune mediated diseases Task Force**
Cameron Adams	Genentech, San Francisco, CA, United States	Clinical Task Forces (FinnGen phase 3)	**Immune mediated diseases Task Force**
Mark McCarthy	Genentech, San Francisco, CA, United States	Clinical Task Forces (FinnGen phase 3)	**Immune mediated diseases Task Force**
Michael Rothenberg	Genentech, San Francisco, CA, United States	Clinical Task Forces (FinnGen phase 3)	**Immune mediated diseases Task Force**
Rion Pendergrass	Genentech, San Francisco, CA, United States	Clinical Task Forces (FinnGen phase 3)	**Immune mediated diseases Task Force**
Diana L.Cousminer	GlaxoSmithKline, Collegeville, PA, United States	Clinical Task Forces (FinnGen phase 3)	**Immune mediated diseases Task Force**
Jagtar Nijjar	GlaxoSmithKline, Collegeville, PA, United States	Clinical Task Forces (FinnGen phase 3)	**Immune mediated diseases Task Force**
Jessica Chao	GlaxoSmithKline, Collegeville, PA, United States	Clinical Task Forces (FinnGen phase 3)	**Immune mediated diseases Task Force**
Joanna C.Betts	GlaxoSmithKline, Collegeville, PA, United States	Clinical Task Forces (FinnGen phase 3)	**Immune mediated diseases Task Force**
Jonathan M.Davitte	GlaxoSmithKline, Collegeville, PA, United States	Clinical Task Forces (FinnGen phase 3)	**Immune mediated diseases Task Force**
Linda McGarthy	GlaxoSmithKline, Collegeville, PA, United States	Clinical Task Forces (FinnGen phase 3)	**Immune mediated diseases Task Force**
Michal Magid	GlaxoSmithKline, Collegeville, PA, United States	Clinical Task Forces (FinnGen phase 3)	**Immune mediated diseases Task Force**
Shashank Jariwala	GlaxoSmithKline, Collegeville, PA, United States	Clinical Task Forces (FinnGen phase 3)	**Immune mediated diseases Task Force**
Dawn Waterworth	Johnson & Johnson Innovative Medicine, Spring House, PA, United States	Clinical Task Forces (FinnGen phase 3)	**Immune mediated diseases Task Force**
Amy Hart	Johnson & Johnson Innovative Medicine, Spring House, PA, United States	Clinical Task Forces (FinnGen phase 3)	**Immune mediated diseases Task Force**

Brice Keyes	Johnson & Johnson Innovative Medicine, Spring House, PA, United States	Clinical Task Forces (FinnGen phase 3)	**Immune mediated diseases Task Force**
John Kwon	Johnson & Johnson Innovative Medicine, Spring House, PA, United States	Clinical Task Forces (FinnGen phase 3)	**Immune mediated diseases Task Force**
Jonathan Sherlock	Johnson & Johnson Innovative Medicine, Spring House, PA, United States	Clinical Task Forces (FinnGen phase 3)	**Immune mediated diseases Task Force**
Matt Loza	Johnson & Johnson Innovative Medicine, Spring House, PA, United States	Clinical Task Forces (FinnGen phase 3)	**Immune mediated diseases Task Force**
Elisabeth Vollmann	Merck, Kenilworth, NJ, United States	Clinical Task Forces (FinnGen phase 3)	**Immune mediated diseases Task Force**
Jozsef Karman	Merck, Kenilworth, NJ, United States	Clinical Task Forces (FinnGen phase 3)	**Immune mediated diseases Task Force**
Julie Fiore	Merck, Kenilworth, NJ, United States	Clinical Task Forces (FinnGen phase 3)	**Immune mediated diseases Task Force**
Rajesh Kamath	Merck, Kenilworth, NJ, United States	Clinical Task Forces (FinnGen phase 3)	**Immune mediated diseases Task Force**
Enrico Ferrero	Novartis Institutes for BioMedical Research, Cambridge, MA, United States	Clinical Task Forces (FinnGen phase 3)	**Immune mediated diseases Task Force**
Jonas Zierer	Novartis Institutes for BioMedical Research, Cambridge, MA, United States	Clinical Task Forces (FinnGen phase 3)	**Immune mediated diseases Task Force**
Nikos Patsopoulos	Novartis Institutes for BioMedical Research, Cambridge, MA, United States	Clinical Task Forces (FinnGen phase 3)	**Immune mediated diseases Task Force**
Erin Macdonald-Dunlop	Pfizer, New York, NY, United States	Clinical Task Forces (FinnGen phase 3)	**Immune mediated diseases Task Force**
Jessica Chung	Pfizer, New York, NY, United States	Clinical Task Forces (FinnGen phase 3)	**Immune mediated diseases Task Force**
Michael McLean	Pfizer, New York, NY, United States	Clinical Task Forces (FinnGen phase 3)	**Immune mediated diseases Task Force**
Hamid Mattoo	Translational Sciences, Sanofi R&D, Framingham, MA, USA	Clinical Task Forces (FinnGen phase 3)	**Immune mediated diseases Task Force**
Aarno Palotie	Institute for Molecular Medicine Finland (FIMM), HiLIFE, University of Helsinki, Helsinki, Finland; Broad Institute of MIT and Harvard; Massachusetts General Hospital, Boston, MA, United States	Clinical Task Forces (FinnGen phase 3)	**Kidney diseases Task Force**
Elisa Lahtela	Institute for Molecular Medicine Finland (FIMM), HiLIFE, University of Helsinki, Helsinki, Finland	Clinical Task Forces (FinnGen phase 3)	**Kidney diseases Task Force**
Helen Cooper	Institute for Molecular Medicine Finland (FIMM), HiLIFE, University of Helsinki, Helsinki, Finland	Clinical Task Forces (FinnGen phase 3)	**Kidney diseases Task Force**
Jukka Koskela	Institute for Molecular Medicine Finland (FIMM), HiLIFE, University of Helsinki, Helsinki, Finland	Clinical Task Forces (FinnGen phase 3)	**Kidney diseases Task Force**
Mark Daly	Institute for Molecular Medicine Finland (FIMM), HiLIFE, University of Helsinki, Helsinki, Finland; Broad Institute of MIT and Harvard; Massachusetts General Hospital, Boston, MA, United States	Clinical Task Forces (FinnGen phase 3)	**Kidney diseases Task Force**
Mary Pat Reeve	Institute for Molecular Medicine Finland (FIMM), HiLIFE, University of Helsinki, Helsinki, Finland; Broad Institute, Cambridge, MA, United States	Clinical Task Forces (FinnGen phase 3)	**Kidney diseases Task Force**
Raymond Walters	Institute for Molecular Medicine Finland (FIMM), HiLIFE, University of Helsinki, Helsinki, Finland; Broad Institute, Cambridge, MA, United States	Clinical Task Forces (FinnGen phase 3)	**Kidney diseases Task Force**
Rodos Rodosthenous	Institute for Molecular Medicine Finland (FIMM), HiLIFE, University of Helsinki, Helsinki, Finland	Clinical Task Forces (FinnGen phase 3)	**Kidney diseases Task Force**
Jouni Lauronen	Finnish Red Cross Blood Service / Finnish Hematology Registry and Clinical Biobank, Helsinki, Finland	Clinical Task Forces (FinnGen phase 3)	**Kidney diseases Task Force**

Adrian Banerji	Institute for Molecular Medicine Finland (FIMM), HiLIFE, University of Helsinki, Helsinki, Finland; Broad Institute & Harvard Medical School, Cambridge, United States	**Clinical Task Forces (FinnGen phase 3)**	**Kidney diseases Task Force**
Matthew Sampson	Broad Institute, Cambridge, MA, United States; Harvard Medical School, Cambridge, United States	**Clinical Task Forces (FinnGen phase 3)**	**Kidney diseases Task Force**
Michelle McNulty	Institute for Molecular Medicine Finland (FIMM), HiLIFE, University of Helsinki, Helsinki, Finland; Broad Institute & Harvard Medical School, Cambridge, United States	**Clinical Task Forces (FinnGen phase 3)**	**Kidney diseases Task Force**
Daniel Gordin	Helsinki University Hospital and University of Helsinki, Helsinki, Finland	**Clinical Task Forces (FinnGen phase 3)**	**Kidney diseases Task Force**
Patrik Finne	Helsinki University Hospital and University of Helsinki, Helsinki, Finland	**Clinical Task Forces (FinnGen phase 3)**	**Kidney diseases Task Force**
Mika Kähönen	Finnish Clinical Biobank Tampere / University of Tampere / Wellbeing Services County of Pirkanmaa, Tampere, Finland	**Clinical Task Forces (FinnGen phase 3)**	**Kidney diseases Task Force**
Tapio Hellman	University of Turku, Turku, Finland	**Clinical Task Forces (FinnGen phase 3)**	**Kidney diseases Task Force**
Teemu Niiranen	University of Turku, Turku, Finland; Finnish Institute for Health and Welfare (THL), Helsinki, Finland	**Clinical Task Forces (FinnGen phase 3)**	**Kidney diseases Task Force**
Dirk Paul	Astra Zeneca, Cambridge, United Kingdom	**Clinical Task Forces (FinnGen phase 3)**	**Kidney diseases Task Force**
Ioanna Tachmazidou	Astra Zeneca, Cambridge, United Kingdom	**Clinical Task Forces (FinnGen phase 3)**	**Kidney diseases Task Force**
Hans van Leeuwen	Bayer AG, Leverkusen, Germany	**Clinical Task Forces (FinnGen phase 3)**	**Kidney diseases Task Force**
Johanna Mielke	Bayer AG, Leverkusen, Germany	**Clinical Task Forces (FinnGen phase 3)**	**Kidney diseases Task Force**
Juho Immonen	Bayer AG, Leverkusen, Germany	**Clinical Task Forces (FinnGen phase 3)**	**Kidney diseases Task Force**
Thomas Battram	Bayer AG, Leverkusen, Germany	**Clinical Task Forces (FinnGen phase 3)**	**Kidney diseases Task Force**
Tobias Hogrebe	Bayer AG, Leverkusen, Germany	**Clinical Task Forces (FinnGen phase 3)**	**Kidney diseases Task Force**
Ketian Yu	Biogen, Cambridge, MA, United States	**Clinical Task Forces (FinnGen phase 3)**	**Kidney diseases Task Force**
Benjamin Sun	Bristol Myers Squibb, New York, NY, United States	**Clinical Task Forces (FinnGen phase 3)**	**Kidney diseases Task Force**
Janie Shelton	Bristol Myers Squibb, New York, NY, United States	**Clinical Task Forces (FinnGen phase 3)**	**Kidney diseases Task Force**
Yao Hu	Boehringer Ingelheim, Ingelheim am Rhein, Germany	**Clinical Task Forces (FinnGen phase 3)**	**Kidney diseases Task Force**
Zhihao Ding	Boehringer Ingelheim, Ingelheim am Rhein, Germany	**Clinical Task Forces (FinnGen phase 3)**	**Kidney diseases Task Force**
Rion Pendergrass	Genentech, San Francisco, CA, United States	**Clinical Task Forces (FinnGen phase 3)**	**Kidney diseases Task Force**
Sergio Dellepiane	Genentech, San Francisco, CA, United States	**Clinical Task Forces (FinnGen phase 3)**	**Kidney diseases Task Force**
Audrey Chu	GlaxoSmithKline, Collegeville, PA, United States	**Clinical Task Forces (FinnGen phase 3)**	**Kidney diseases Task Force**
Chris Floyd	GlaxoSmithKline, Collegeville, PA, United States	**Clinical Task Forces (FinnGen phase 3)**	**Kidney diseases Task Force**

Dan Swerdlow	GlaxoSmithKline, Collegeville, PA, United States	Clinical Task Forces (FinnGen phase 3)	**Kidney diseases Task Force**
Erding Hu	GlaxoSmithKline, Collegeville, PA, United States	Clinical Task Forces (FinnGen phase 3)	**Kidney diseases Task Force**
Jonathan Davitte	GlaxoSmithKline, Collegeville, PA, United States	Clinical Task Forces (FinnGen phase 3)	**Kidney diseases Task Force**
Prerak Desai	GlaxoSmithKline, Collegeville, PA, United States	Clinical Task Forces (FinnGen phase 3)	**Kidney diseases Task Force**
Stephen Haddad	GlaxoSmithKline, Collegeville, PA, United States	Clinical Task Forces (FinnGen phase 3)	**Kidney diseases Task Force**
Dermot Reilly	Johnson & Johnson Innovative Medicine, Spring House, PA, United States	Clinical Task Forces (FinnGen phase 3)	**Kidney diseases Task Force**
P. Dunnmon	Johnson & Johnson Innovative Medicine, Spring House, PA, United States	Clinical Task Forces (FinnGen phase 3)	**Kidney diseases Task Force**
Karol Estrada	Maze Therapeutics, San Francisco, CA, United States	Clinical Task Forces (FinnGen phase 3)	**Kidney diseases Task Force**
Rob Graham	Maze Therapeutics, San Francisco, CA, United States	Clinical Task Forces (FinnGen phase 3)	**Kidney diseases Task Force**
Sahar Mozzafari	Maze Therapeutics, San Francisco, CA, United States	Clinical Task Forces (FinnGen phase 3)	**Kidney diseases Task Force**
Nancy Finkel	Novartis Institutes for BioMedical Research, Cambridge, MA, United States	Clinical Task Forces (FinnGen phase 3)	**Kidney diseases Task Force**
Sabina Pfister	Novartis Institutes for BioMedical Research, Cambridge, MA, United States	Clinical Task Forces (FinnGen phase 3)	**Kidney diseases Task Force**
Shola Richards	Novartis Institutes for BioMedical Research, Cambridge, MA, United States	Clinical Task Forces (FinnGen phase 3)	**Kidney diseases Task Force**
Joshua Chiou	Pfizer, New York, NY, United States	Clinical Task Forces (FinnGen phase 3)	**Kidney diseases Task Force**
Ying Wu	Pfizer, New York, NY, United States	Clinical Task Forces (FinnGen phase 3)	**Kidney diseases Task Force**
Katherine Klinger	Translational Sciences, Sanofi R&D, Framingham, MA, USA	Clinical Task Forces (FinnGen phase 3)	**Kidney diseases Task Force**
Matti Vuori	University of Turku, Turku, Finland	Clinical Task Forces (FinnGen phase 3)	**Metabolic diseases Task Force**
Teemu Niiranen	University of Turku, Turku, Finland; Finnish Institute for Health and Welfare (THL), Helsinki, Finland	Clinical Task Forces (FinnGen phase 3)	**Metabolic diseases Task Force**
Bridget Riley-Gillis	AbbVie, Chicago, IL, United States	Clinical Task Forces (FinnGen phase 3)	**Metabolic diseases Task Force**
Nizar Smaoui	AbbVie, Chicago, IL, United States	Clinical Task Forces (FinnGen phase 3)	**Metabolic diseases Task Force**
Alix Berton	Bayer AG, Leverkusen, Germany	Clinical Task Forces (FinnGen phase 3)	**Metabolic diseases Task Force**
Hans van Leeuwen	Bayer AG, Leverkusen, Germany	Clinical Task Forces (FinnGen phase 3)	**Metabolic diseases Task Force**
Chen Li	Bristol Myers Squibb, New York, NY, United States	Clinical Task Forces (FinnGen phase 3)	**Metabolic diseases Task Force**
Emily Holzinger	Bristol Myers Squibb, New York, NY, United States	Clinical Task Forces (FinnGen phase 3)	**Metabolic diseases Task Force**

Anubha Mahajan	Genentech, San Francisco, CA, United States	**Clinical Task Forces (FinnGen phase 3)**	**Metabolic diseases Task Force**
Mark Mccarthy	Genentech, San Francisco, CA, United States	**Clinical Task Forces (FinnGen phase 3)**	**Metabolic diseases Task Force**
Christopher Deboever	Maze Therapeutics, San Francisco, CA, United States	**Clinical Task Forces (FinnGen phase 3)**	**Metabolic diseases Task Force**
Karol Estrada	Maze Therapeutics, San Francisco, CA, United States	**Clinical Task Forces (FinnGen phase 3)**	**Metabolic diseases Task Force**
Robert Graham	Maze Therapeutics, San Francisco, CA, United States	**Clinical Task Forces (FinnGen phase 3)**	**Metabolic diseases Task Force**
Hye In Kim	Pfizer, New York, NY, United States	**Clinical Task Forces (FinnGen phase 3)**	**Metabolic diseases Task Force**
Sivakumar Pitchumani	Pfizer, New York, NY, United States	**Clinical Task Forces (FinnGen phase 3)**	**Metabolic diseases Task Force**
Sumedha Jassal	Pfizer, New York, NY, United States	**Clinical Task Forces (FinnGen phase 3)**	**Metabolic diseases Task Force**
Åsa Hedman	Pfizer, New York, NY, United States	**Clinical Task Forces (FinnGen phase 3)**	**Metabolic diseases Task Force**
Aarno Palotie	Institute for Molecular Medicine Finland (FIMM), HiLIFE, University of Helsinki, Helsinki, Finland; Broad Institute of MIT and Harvard; Massachusetts General Hospital, Boston, MA, United States	**Clinical Task Forces (FinnGen phase 3)**	**Neurodegenerative diseases Task Force**
Austin Argentieri	Institute for Molecular Medicine Finland (FIMM), HiLIFE, University of Helsinki, Helsinki, Finland; Broad Institute, Cambridge, MA, United States	**Clinical Task Forces (FinnGen phase 3)**	**Neurodegenerative diseases Task Force**
Aoxing Liu	Institute for Molecular Medicine Finland (FIMM), HiLIFE, University of Helsinki, Helsinki, Finland	**Clinical Task Forces (FinnGen phase 3)**	**Neurodegenerative diseases Task Force**
Eero Vuoksimaa	Institute for Molecular Medicine Finland (FIMM), HiLIFE, University of Helsinki, Helsinki, Finland	**Clinical Task Forces (FinnGen phase 3)**	**Neurodegenerative diseases Task Force**
Elisa Lahtela	Institute for Molecular Medicine Finland (FIMM), HiLIFE, University of Helsinki, Helsinki, Finland	**Clinical Task Forces (FinnGen phase 3)**	**Neurodegenerative diseases Task Force**
Joni Lindbohm	Institute for Molecular Medicine Finland (FIMM), HiLIFE, University of Helsinki, Helsinki, Finland	**Clinical Task Forces (FinnGen phase 3)**	**Neurodegenerative diseases Task Force**
Mark Daly	Institute for Molecular Medicine Finland (FIMM), HiLIFE, University of Helsinki, Helsinki, Finland; Broad Institute of MIT and Harvard; Massachusetts General Hospital, Boston, MA, United States	**Clinical Task Forces (FinnGen phase 3)**	**Neurodegenerative diseases Task Force**
Mary Pat Reeve	Institute for Molecular Medicine Finland (FIMM), HiLIFE, University of Helsinki, Helsinki, Finland; Broad Institute, Cambridge, MA, United States	**Clinical Task Forces (FinnGen phase 3)**	**Neurodegenerative diseases Task Force**
Paavo Häppölä	Institute for Molecular Medicine Finland (FIMM), HiLIFE, University of Helsinki, Helsinki, Finland	**Clinical Task Forces (FinnGen phase 3)**	**Neurodegenerative diseases Task Force**
Zhiyu Yang	Institute for Molecular Medicine Finland (FIMM), HiLIFE, University of Helsinki, Helsinki, Finland	**Clinical Task Forces (FinnGen phase 3)**	**Neurodegenerative diseases Task Force**
Eino Solje	University of Eastern Finland, Kuopio, Finland	**Clinical Task Forces (FinnGen phase 3)**	**Neurodegenerative diseases Task Force**
Mikko Hiltunen	University of Eastern Finland, Kuopio, Finland	**Clinical Task Forces (FinnGen phase 3)**	**Neurodegenerative diseases Task Force**
Valtteri Julkunen	University of Eastern Finland and Kuopio University Hospital, Kuopio, Finland	**Clinical Task Forces (FinnGen phase 3)**	**Neurodegenerative diseases Task Force**
Ville Leinonen	University of Eastern Finland and Kuopio University Hospital, Kuopio, Finland	**Clinical Task Forces (FinnGen phase 3)**	**Neurodegenerative diseases Task Force**
Hanna Kujala	Biobank of Eastern Finland / University of Eastern Finland / Wellbeing services county of North Savo, Kuopio, Finland	**Clinical Task Forces (FinnGen phase 3)**	**Neurodegenerative diseases Task Force**

Aki Havulinna	Finnish Institute for Health and Welfare (THL), Helsinki, Finland	Clinical Task Forces (FinnGen phase 3)	**Neurodegenerative diseases Task Force**
Roosa Kallionpää	University of Turku, Turku, Finland	Clinical Task Forces (FinnGen phase 3)	**Neurodegenerative diseases Task Force**
Minttu Marttila	University of Helsinki, Helsinki, Finland	Clinical Task Forces (FinnGen phase 3)	**Neurodegenerative diseases Task Force**
Britney Milkovich	AbbVie, Chicago, IL, United States	Clinical Task Forces (FinnGen phase 3)	**Neurodegenerative diseases Task Force**
Jan Freudenberg	AbbVie, Chicago, IL, United States	Clinical Task Forces (FinnGen phase 3)	**Neurodegenerative diseases Task Force**
Andrew Lowe	Astra Zeneca, Cambridge, United Kingdom	Clinical Task Forces (FinnGen phase 3)	**Neurodegenerative diseases Task Force**
Ioanna Tachmazidou	Astra Zeneca, Cambridge, United Kingdom	Clinical Task Forces (FinnGen phase 3)	**Neurodegenerative diseases Task Force**
Thomas Spargo	Astra Zeneca, Cambridge, United Kingdom	Clinical Task Forces (FinnGen phase 3)	**Neurodegenerative diseases Task Force**
Kritika Singh	Bristol Myers Squibb, New York, NY, United States	Clinical Task Forces (FinnGen phase 3)	**Neurodegenerative diseases Task Force**
Peng Jiang	Bristol Myers Squibb, New York, NY, United States	Clinical Task Forces (FinnGen phase 3)	**Neurodegenerative diseases Task Force**
Stephanie Loomis	Bristol Myers Squibb, New York, NY, United States	Clinical Task Forces (FinnGen phase 3)	**Neurodegenerative diseases Task Force**
Anubha Mahajan	Genentech, San Francisco, CA, United States	Clinical Task Forces (FinnGen phase 3)	**Neurodegenerative diseases Task Force**
Rion Pendergrass	Genentech, San Francisco, CA, United States	Clinical Task Forces (FinnGen phase 3)	**Neurodegenerative diseases Task Force**
Damien Croteau-Chonka	GlaxoSmithKline, Collegeville, PA, United States	Clinical Task Forces (FinnGen phase 3)	**Neurodegenerative diseases Task Force**
John Eicher	GlaxoSmithKline, Collegeville, PA, United States	Clinical Task Forces (FinnGen phase 3)	**Neurodegenerative diseases Task Force**
Prerak Desai	GlaxoSmithKline, Collegeville, PA, United States	Clinical Task Forces (FinnGen phase 3)	**Neurodegenerative diseases Task Force**
Chris Whelan	Johnson & Johnson Innovative Medicine, Spring House, PA, United States	Clinical Task Forces (FinnGen phase 3)	**Neurodegenerative diseases Task Force**
Karen He	Johnson & Johnson Innovative Medicine, Spring House, PA, United States	Clinical Task Forces (FinnGen phase 3)	**Neurodegenerative diseases Task Force**
Qingqin Li	Johnson & Johnson Innovative Medicine, Spring House, PA, United States	Clinical Task Forces (FinnGen phase 3)	**Neurodegenerative diseases Task Force**
W Galpern	Johnson & Johnson Innovative Medicine, Spring House, PA, United States	Clinical Task Forces (FinnGen phase 3)	**Neurodegenerative diseases Task Force**
Yanfei Zhang	Johnson & Johnson Innovative Medicine, Spring House, PA, United States	Clinical Task Forces (FinnGen phase 3)	**Neurodegenerative diseases Task Force**
Andrei Popescu	Merck, Kenilworth, NJ, United States	Clinical Task Forces (FinnGen phase 3)	**Neurodegenerative diseases Task Force**
Delphine Fagegaltier	Merck, Kenilworth, NJ, United States	Clinical Task Forces (FinnGen phase 3)	**Neurodegenerative diseases Task Force**
Mari Niemi	Novartis Institutes for BioMedical Research, Cambridge, MA, United States	Clinical Task Forces (FinnGen phase 3)	**Neurodegenerative diseases Task Force**

Nikos Patsopoulos	Novartis Institutes for BioMedical Research, Cambridge, MA, United States	Clinical Task Forces (FinnGen phase 3)	**Neurodegenerative diseases Task Force**
Katherine Klinger	Translational Sciences, Sanofi R&D, Framingham, MA, USA	Clinical Task Forces (FinnGen phase 3)	**Neurodegenerative diseases Task Force**
Aarno Palotie	Institute for Molecular Medicine Finland (FIMM), HiLIFE, University of Helsinki, Helsinki, Finland; Broad Institute of MIT and Harvard; Massachusetts General Hospital, Boston, MA, United States	Clinical Task Forces (FinnGen phase 3)	**Pulmonology Task Force and fibrotic diseases interest group**
Elisa Lahtela	Institute for Molecular Medicine Finland (FIMM), HiLIFE, University of Helsinki, Helsinki, Finland	Clinical Task Forces (FinnGen phase 3)	**Pulmonology Task Force and fibrotic diseases interest group**
Jukka Koskela	Institute for Molecular Medicine Finland (FIMM), HiLIFE, University of Helsinki, Helsinki, Finland	Clinical Task Forces (FinnGen phase 3)	**Pulmonology Task Force and fibrotic diseases interest group**
Mark Daly	Institute for Molecular Medicine Finland (FIMM), HiLIFE, University of Helsinki, Helsinki, Finland; Broad Institute of MIT and Harvard; Massachusetts General Hospital, Boston, MA, United States	Clinical Task Forces (FinnGen phase 3)	**Pulmonology Task Force and fibrotic diseases interest group**
Sanni Ruotsalainen	Institute for Molecular Medicine Finland (FIMM), HiLIFE, University of Helsinki, Helsinki, Finland	Clinical Task Forces (FinnGen phase 3)	**Pulmonology Task Force and fibrotic diseases interest group**
Susanna Lemmelä	Institute for Molecular Medicine Finland (FIMM), HiLIFE, University of Helsinki, Helsinki, Finland	Clinical Task Forces (FinnGen phase 3)	**Pulmonology Task Force and fibrotic diseases interest group**
Tarja Laitinen	Institute for Molecular Medicine Finland (FIMM), HiLIFE, University of Helsinki, Helsinki, Finland	Clinical Task Forces (FinnGen phase 3)	**Pulmonology Task Force and fibrotic diseases interest group**
Salla Ranta	Hospital District of Helsinki and Uusimaa, Helsinki, Finland	Clinical Task Forces (FinnGen phase 3)	**Pulmonology Task Force and fibrotic diseases interest group**
Paavo Häppölä	Institute for Molecular Medicine Finland (FIMM), HiLIFE, University of Helsinki, Helsinki, Finland	Clinical Task Forces (FinnGen phase 3)	**Pulmonology Task Force and fibrotic diseases interest group**
Paula Kauppi	Hospital District of Helsinki and Uusimaa, Helsinki, Finland	Clinical Task Forces (FinnGen phase 3)	**Pulmonology Task Force and fibrotic diseases interest group**
Tiinamaija Tuomi	Institute for Molecular Medicine Finland (FIMM), HiLIFE, University of Helsinki, Helsinki, Finland; Hospital District of Helsinki and Uusimaa, Helsinki, Finland	Clinical Task Forces (FinnGen phase 3)	**Pulmonology Task Force and fibrotic diseases interest group**
Raisa Serpi	Northern Finland Biobank Borealis / University of Oulu / Wellbeing services county of North Ostrobothnia, Oulu, Finland	Clinical Task Forces (FinnGen phase 3)	**Pulmonology Task Force and fibrotic diseases interest group**
Riitta Kaarteenaho	University of Oulu, Oulu, Finland	Clinical Task Forces (FinnGen phase 3)	**Pulmonology Task Force and fibrotic diseases interest group**
Hannu Kankaanranta	University of Gothenburg, Gothenburg, Sweden/ Seinäjoki Central Hospital, Seinäjoki, Finland/ Tampere University, Tampere, Finland	Clinical Task Forces (FinnGen phase 3)	**Pulmonology Task Force and fibrotic diseases interest group**
Coralie Viollet	Astra Zeneca, Cambridge, United Kingdom	Clinical Task Forces (FinnGen phase 3)	**Pulmonology Task Force and fibrotic diseases interest group**
Eleanor Wheeler	Astra Zeneca, Cambridge, United Kingdom	Clinical Task Forces (FinnGen phase 3)	**Pulmonology Task Force and fibrotic diseases interest group**
Oliver Burren	Astra Zeneca, Cambridge, United Kingdom	Clinical Task Forces (FinnGen phase 3)	**Pulmonology Task Force and fibrotic diseases interest group**
Christoph Ogris	Boehringer Ingelheim, Ingelheim am Rhein, Germany	Clinical Task Forces (FinnGen phase 3)	**Pulmonology Task Force and fibrotic diseases interest group**
Eric Simon	Boehringer Ingelheim, Ingelheim am Rhein, Germany	Clinical Task Forces (FinnGen phase 3)	**Pulmonology Task Force and fibrotic diseases interest group**
Frank LI	Boehringer Ingelheim, Ingelheim am Rhein, Germany	Clinical Task Forces (FinnGen phase 3)	**Pulmonology Task Force and fibrotic diseases interest group**
Julio Cesar Bolivar Lopez	Boehringer Ingelheim, Ingelheim am Rhein, Germany	Clinical Task Forces (FinnGen phase 3)	**Pulmonology Task Force and fibrotic diseases interest group**
Yao Hu	Boehringer Ingelheim, Ingelheim am Rhein, Germany	Clinical Task Forces (FinnGen phase 3)	**Pulmonology Task Force and fibrotic diseases interest group**

Zhihao Ding	Boehringer Ingelheim, Ingelheim am Rhein, Germany	**Clinical Task Forces (FinnGen phase 3)**	**Pulmonology Task Force and fibrotic diseases interest group**
Elena Sanchez	Bristol Myers Squibb, New York, NY, United States	**Clinical Task Forces (FinnGen phase 3)**	**Pulmonology Task Force and fibrotic diseases interest group**
Emily Holzinger	Bristol Myers Squibb, New York, NY, United States	**Clinical Task Forces (FinnGen phase 3)**	**Pulmonology Task Force and fibrotic diseases interest group**
Joe Maranville	Bristol Myers Squibb, New York, NY, United States	**Clinical Task Forces (FinnGen phase 3)**	**Pulmonology Task Force and fibrotic diseases interest group**
Lilith Moss	Bristol Myers Squibb, New York, NY, United States	**Clinical Task Forces (FinnGen phase 3)**	**Pulmonology Task Force and fibrotic diseases interest group**
Michael Turchin	Bristol Myers Squibb, New York, NY, United States	**Clinical Task Forces (FinnGen phase 3)**	**Pulmonology Task Force and fibrotic diseases interest group**
Zijie Zhao	Bristol Myers Squibb, New York, NY, United States	**Clinical Task Forces (FinnGen phase 3)**	**Pulmonology Task Force and fibrotic diseases interest group**
Diana Chang	Genentech, San Francisco, CA, United States	**Clinical Task Forces (FinnGen phase 3)**	**Pulmonology Task Force and fibrotic diseases interest group**
Audrey Chu	GlaxoSmithKline, Collegeville, PA, United States	**Clinical Task Forces (FinnGen phase 3)**	**Pulmonology Task Force and fibrotic diseases interest group**
Billy Fahy	GlaxoSmithKline, Collegeville, PA, United States	**Clinical Task Forces (FinnGen phase 3)**	**Pulmonology Task Force and fibrotic diseases interest group**
Jessica Chao	GlaxoSmithKline, Collegeville, PA, United States	**Clinical Task Forces (FinnGen phase 3)**	**Pulmonology Task Force and fibrotic diseases interest group**
Joanna Betts	GlaxoSmithKline, Collegeville, PA, United States	**Clinical Task Forces (FinnGen phase 3)**	**Pulmonology Task Force and fibrotic diseases interest group**
Jonathan Davitte	GlaxoSmithKline, Collegeville, PA, United States	**Clinical Task Forces (FinnGen phase 3)**	**Pulmonology Task Force and fibrotic diseases interest group**
Paola Bronson	GlaxoSmithKline, Collegeville, PA, United States	**Clinical Task Forces (FinnGen phase 3)**	**Pulmonology Task Force and fibrotic diseases interest group**
Prerak Desai	GlaxoSmithKline, Collegeville, PA, United States	**Clinical Task Forces (FinnGen phase 3)**	**Pulmonology Task Force and fibrotic diseases interest group**
Dermot Reilly	Johnson & Johnson Innovative Medicine, Spring House, PA, United States	**Clinical Task Forces (FinnGen phase 3)**	**Pulmonology Task Force and fibrotic diseases interest group**
Mona Selej	Johnson & Johnson Innovative Medicine, Spring House, PA, United States	**Clinical Task Forces (FinnGen phase 3)**	**Pulmonology Task Force and fibrotic diseases interest group**
P Dunnmon	Johnson & Johnson Innovative Medicine, Spring House, PA, United States	**Clinical Task Forces (FinnGen phase 3)**	**Pulmonology Task Force and fibrotic diseases interest group**
Jorge Del-aguila	Merck, Kenilworth, NJ, United States	**Clinical Task Forces (FinnGen phase 3)**	**Pulmonology Task Force and fibrotic diseases interest group**
Jozsef Karman	Merck, Kenilworth, NJ, United States	**Clinical Task Forces (FinnGen phase 3)**	**Pulmonology Task Force and fibrotic diseases interest group**
Travis Barr	Merck, Kenilworth, NJ, United States	**Clinical Task Forces (FinnGen phase 3)**	**Pulmonology Task Force and fibrotic diseases interest group**
Katherine Mccauley	Novartis Institutes for BioMedical Research, Cambridge, MA, United States	**Clinical Task Forces (FinnGen phase 3)**	**Pulmonology Task Force and fibrotic diseases interest group**
Xiaobo Xia	Novartis Institutes for BioMedical Research, Cambridge, MA, United States	**Clinical Task Forces (FinnGen phase 3)**	**Pulmonology Task Force and fibrotic diseases interest group**
Madhurima Saxena	Pfizer, New York, NY, United States	**Clinical Task Forces (FinnGen phase 3)**	**Pulmonology Task Force and fibrotic diseases interest group**

Pitchumani Sivakumar	Pfizer, New York, NY, United States	Clinical Task Forces (FinnGen phase 3)	**Pulmonology Task Force and fibrotic diseases interest group**
Sumedha Jassal	Pfizer, New York, NY, United States	Clinical Task Forces (FinnGen phase 3)	**Pulmonology Task Force and fibrotic diseases interest group**
David Habiel	Translational Sciences, Sanofi R&D, Framingham, MA, USA	Clinical Task Forces (FinnGen phase 3)	**Pulmonology Task Force and fibrotic diseases interest group**
Guanling Huan	Translational Sciences, Sanofi R&D, Framingham, MA, USA	Clinical Task Forces (FinnGen phase 3)	**Pulmonology Task Force and fibrotic diseases interest group**
Marika Kaakinen	Institute for Molecular Medicine Finland (FIMM), HiLIFE, University of Helsinki, Helsinki, Finland	Clinical Task Forces (FinnGen phase 3)	**Parkinson´s disease Task Force**
Mary Pat Reeve	Institute for Molecular Medicine Finland (FIMM), HiLIFE, University of Helsinki, Helsinki, Finland; Broad Institute, Cambridge, MA, United States	Clinical Task Forces (FinnGen phase 3)	**Parkinson´s disease Task Force**
Filip Scheperjans	Hospital District of Helsinki and Uusimaa, Helsinki, Finland	Clinical Task Forces (FinnGen phase 3)	**Parkinson´s disease Task Force**
Andrew Blumenfeld	AbbVie, Chicago, IL, United States	Clinical Task Forces (FinnGen phase 3)	**Parkinson´s disease Task Force**
Britney Milkovich	AbbVie, Chicago, IL, United States	Clinical Task Forces (FinnGen phase 3)	**Parkinson´s disease Task Force**
Jan Freudenberg	AbbVie, Chicago, IL, United States	Clinical Task Forces (FinnGen phase 3)	**Parkinson´s disease Task Force**
Tushar Kumar	AbbVie, Chicago, IL, United States	Clinical Task Forces (FinnGen phase 3)	**Parkinson´s disease Task Force**
Hans van Leeuwen	Bayer AG, Leverkusen, Germany	Clinical Task Forces (FinnGen phase 3)	**Parkinson´s disease Task Force**
Juho Immonen	Bayer AG, Leverkusen, Germany	Clinical Task Forces (FinnGen phase 3)	**Parkinson´s disease Task Force**
Samu Kurki	Bayer AG, Leverkusen, Germany	Clinical Task Forces (FinnGen phase 3)	**Parkinson´s disease Task Force**
Coro Paisan-Ruiz	Biogen, Cambridge, MA, United States	Clinical Task Forces (FinnGen phase 3)	**Parkinson´s disease Task Force**
Anna Podgornaia	Bristol Myers Squibb, New York, NY, United States	Clinical Task Forces (FinnGen phase 3)	**Parkinson´s disease Task Force**
Benjamin Sun	Bristol Myers Squibb, New York, NY, United States	Clinical Task Forces (FinnGen phase 3)	**Parkinson´s disease Task Force**
Janie Shelton	Bristol Myers Squibb, New York, NY, United States	Clinical Task Forces (FinnGen phase 3)	**Parkinson´s disease Task Force**
Peng Jiang	Bristol Myers Squibb, New York, NY, United States	Clinical Task Forces (FinnGen phase 3)	**Parkinson´s disease Task Force**
Stephanie Loomis	Bristol Myers Squibb, New York, NY, United States	Clinical Task Forces (FinnGen phase 3)	**Parkinson´s disease Task Force**
Tushar Bhangale	Genentech, San Francisco, CA, United States	Clinical Task Forces (FinnGen phase 3)	**Parkinson´s disease Task Force**
John Eicher	GlaxoSmithKline, Collegeville, PA, United States	Clinical Task Forces (FinnGen phase 3)	**Parkinson´s disease Task Force**
Abolfazl Doostparast Torshizi	Johnson & Johnson Innovative Medicine, Spring House, PA, United States	Clinical Task Forces (FinnGen phase 3)	**Parkinson´s disease Task Force**
Aristide Merola	Merck, Kenilworth, NJ, United States	Clinical Task Forces (FinnGen phase 3)	**Parkinson´s disease Task Force**

Oliver Freeman	Merck, Kenilworth, NJ, United States	**Clinical Task Forces (FinnGen phase 3)**	**Parkinson´s disease Task Force**
Simonne Longerich	Merck, Kenilworth, NJ, United States	**Clinical Task Forces (FinnGen phase 3)**	**Parkinson´s disease Task Force**
Mari Niemi	Novartis Institutes for BioMedical Research, Cambridge, MA, United States	**Clinical Task Forces (FinnGen phase 3)**	**Parkinson´s disease Task Force**
Katherine Klinger	Translational Sciences, Sanofi R&D, Framingham, MA, USA	**Clinical Task Forces (FinnGen phase 3)**	**Parkinson´s disease Task Force**
